# Identification of a Specific Biomarker of Acinetobacter baumannii Global Clone 1 by Machine Learning and PCR Related to Metabolic Fitness of ESKAPE Pathogens

**DOI:** 10.1128/msystems.00734-22

**Published:** 2023-05-15

**Authors:** Verónica Elizabeth Álvarez, María Paula Quiroga, Daniela Centrón

**Affiliations:** a Laboratorio de Investigaciones en Mecanismos de Resistencia a Antibióticos (LIMRA), Instituto de Investigaciones en Microbiología y Parasitología Médica, Facultad de Medicina, Universidad de Buenos Aires—Consejo Nacional de Investigaciones Científicas y Tecnológicas (IMPaM, UBA-CONICET), Ciudad Autónoma de Buenos Aires, Argentina; b Nodo de Bioinformática. Instituto de Investigaciones en Microbiología y Parasitología Médica, Facultad de Medicina, Universidad de Buenos Aires—Consejo Nacional de Investigaciones Científicas y Técnicas (IMPaM, UBA-CONICET), Ciudad Autónoma de Buenos Aires, Argentina; Drexel University

**Keywords:** *Acinetobacter baumannii*, ESKAPE pathogens, GC1, machine learning, PCR, biomarkers, high-risk clones, metabolic fitness

## Abstract

Since the emergence of high-risk clones worldwide, constant investigations have been undertaken to comprehend the molecular basis that led to their prevalent dissemination in nosocomial settings over time. So far, the complex and multifactorial genetic traits of this type of epidemic clones have allowed only the identification of biomarkers with low specificity. A machine learning algorithm was able to recognize unequivocally a biomarker for early and accurate detection of Acinetobacter baumannii global clone 1 (GC1), one of the most disseminated high-risk clones. A support vector machine model identified the U1 sequence with a length of 367 nucleotides that matched a fragment of the *moaCB* gene, which encodes the molybdenum cofactor biosynthesis C and B proteins. U1 differentiates specifically between A. baumannii GC1 and non-GC1 strains, becoming a suitable biomarker capable of being translated into clinical settings as a molecular typing method for early diagnosis based on PCR as shown here. Since the metabolic pathways of Mo enzymes have been recognized as putative therapeutic targets for ESKAPE (Enterococcus faecium, Staphylococcus aureus, Klebsiella pneumoniae, Acinetobacter baumannii, Pseudomonas aeruginosa, and Enterobacter species) pathogens, our findings highlight that machine learning can also be useful in knowledge gaps of high-risk clones and provides noteworthy support to the literature to identify relevant nosocomial biomarkers for other multidrug-resistant high-risk clones.

**IMPORTANCE** A. baumannii GC1 is an important high-risk clone that rapidly develops extreme drug resistance in the nosocomial niche. Furthermore, several strains have been identified worldwide in environmental samples, exacerbating the risk of human interactions. Early diagnosis is mandatory to limit its dissemination and to outline appropriate antibiotic stewardship schedules. A region with a length of 367 bp (U1) within the *moaCB* gene that is not subjected to lateral genetic transfer or to antibiotic pressures was successfully found by a support vector machine model that predicts A. baumannii GC1 strains. At the same time, research on the group of Mo enzymes proposed this metabolic pathway related to the superbug's metabolism as a potential future drug target site for ESKAPE pathogens due to its central role in bacterial fitness during infection. These findings confirm that machine learning used for the identification of biomarkers of high-risk lineages can also serve to identify putative novel therapeutic target sites.

## INTRODUCTION

Acinetobacter baumannii is an opportunistic and nosocomial Gram-negative pathogen that causes a wide range of nosocomial infections. It is included in the group of ESKAPE (Enterococcus faecium, Staphylococcus aureus, Klebsiella pneumoniae, Acinetobacter baumannii, Pseudomonas aeruginosa, and Enterobacter species) pathogens (1, 2). Nosocomial infections by A. baumannii have increased in recent years, adding to its ability to acquire and spread antibiotic resistance genes to all families of antibiotics, and are considered a serious global threat worldwide (3, 4). The majority of A. baumannii isolates that are broadly resistant to antibiotics belong to two pandemic clones, known as global clones 1 (GC1) and 2 (GC2) (3, 5, 6). Recent epidemiological studies of carbapenem-resistant A. baumannii (CRAB) isolates revealed that A. baumannii GC1 is the prevalent CRAB clone in several countries (7–10). In addition, A. baumannii GC1 strains have been found worldwide in environmental samples from water, soil, and animals (11) (http://www.acinetobacterbaumannii.no/).

Over time, molecular methods with different degrees of resolution have been used to type A. baumannii strains, including amplified fragment length polymorphism analysis, ribotyping, macrorestriction analysis by pulsed-field gel electrophoresis, multiplex PCRs, multilocus sequence typing (MLST), and more recently whole-genome sequencing (WGS) (12–18). A typing scheme based on two multiplex PCRs targeting three genes (*ompA*, *csuE*, and *bla*_OXA-51-like_) has been used for the assignment of A. baumannii isolates to two major PCR-based groups corresponding to A. baumannii GC1 and GC2 (19). Also, since correlation between particular *bla*_OXA-51-like_ alleles and some epidemic lineages has been detected, sequence analysis of the *bla*_OXA-51-like_ gene has been proposed as a useful typing method for A. baumannii isolates (19–21). In agreement with this, a study conducted on 60 A. baumannii isolates collected worldwide demonstrated that isolates belonging to A. baumannii GC1 encoded enzymes from the OXA-69 cluster, which included OXA-69, OXA-92, OXA-107, OXA-110, and OXA-112 enzymes (20). However, not all the isolates encoding the OXA-69 cluster belong to A. baumannii GC1, such as A. baumannii strain A92, which belongs to GC2 (20). An additional typing method of A. baumannii GC1 strains consists of the detection of a 108-bp deletion in the 5′-end-conserved segment (5′-CS) of the class 1 integron located in the AbaR3 genomic island (7). Nevertheless, since AbaR3 is not present in all A. baumannii GC1 strains, this approach serves as a marker for some diverged lineages within A. baumannii GC1 (7). All the previously described methods based on PCR include target genes that are subjected to lateral genetic transfer and/or antibiotic pressure, which represents a limitation for the specificity of the technique.

With the increasing throughput and decreasing cost of DNA sequencing, large numbers of bacterial genomes have been submitted to public databases (22, 23). Genome-wide studies of DNA variation related to antibiotic resistance phenotypes have garnered high public interest, especially since several multidrug-resistant strains have emerged worldwide (24). New candidate biomarkers leading to the identification of resistant pathogens require the study and development of fast, easily applicable, and accurate tools. Furthermore, with the help of computational algorithms, such studies can be conducted at a much larger scale producing more significant results (25–28). Machine learning (ML) algorithms and statistics have been used increasingly to build models that correlate genomic variations with phenotypes that may help to predict bacterial phenotypes and genotypes (29–35). In supervised ML, each learning sample includes the outcome (class label) of interest, and it is used to build a prediction model (36). The model takes an outcome measurement (e.g., a bacterium having a resistant phenotype or genotype) and tries to learn from the available data (e.g., information on genomic mutations) to predict the outcome measure. The developed model is then applied to new and unseen data. The goal of the algorithm is to train a model that accurately foresees the correct outcome for any input (37).

Support vector machine (SVM) is a supervised learning algorithm formally characterized by a separating hyperplane that divides binary data to solve both classification and regression problems. The SVM algorithm aims to correctly classify samples based on examples in the training data set (38–40). On the other hand, the set covering machine (SCM) is a supervised learning algorithm that uses a greedy approach to produce uncharacteristically sparse rule-based models from the input data (41). Both algorithms have been applied to several biological knowledge gaps and proved to be accurate in predicting novel antibacterial agents (42), antibiotic resistance genes (31, 32, 43–45), identification of microorganisms (46–48), and cancer diagnosis (49–52), among others (29, 46, 53–56).

ML can deal with large and diverse data sets to extract relevant information (57). Given the wide use of ML in biology, and the absence of accurate identifiers for early detection of A. baumannii GC1 strains, our study aimed to assess whether ML could be applied to process thousands of genomes to identify a suitable A. baumannii GC1 biomarker. We also wanted to analyze whether ML could be combined with other techniques such as PCR and/or quantitative PCR with high-resolution melting (HRM) assays to provide a molecular typing method capable of being translated into clinical settings. For these reasons, we built predictive models for typing A. baumannii GC1 genomes by training SVM and SCM classifiers. From these classifiers, we identified a new and specific genomic biomarker for the early detection of A. baumannii GC1 strains by a PCR technique not subjected to the selective pressure of antibiotics nor to lateral genetic transfer.

## RESULTS

**FIG 1:**
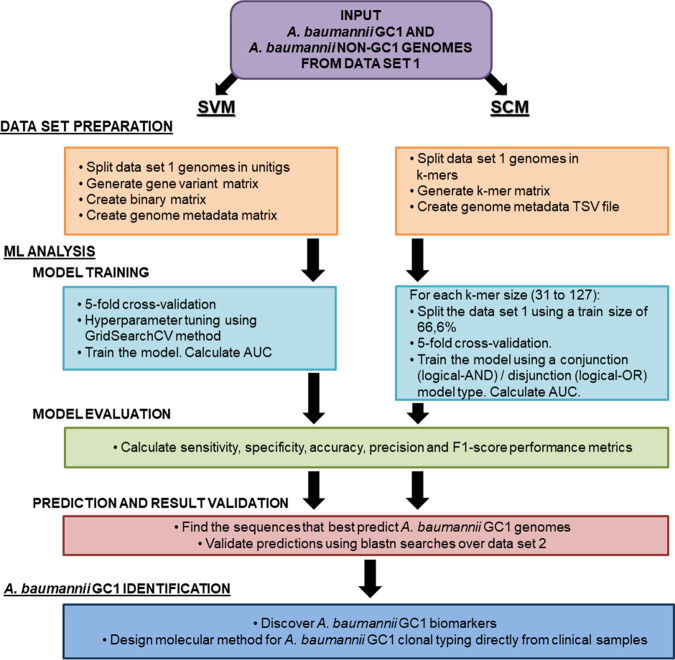
Diagram of the workflow for A. baumannii GC1 biomarker discovery using machine learning. The diagram indicates the steps we followed during the present work related to the genome collections used, data set preparation, ML analysis, and the design of a method for A. baumannii GC1 strain identification.

To identify new genomic biomarkers that uniquely identify strains belonging to A. baumannii GC1, we applied the SVM and SCM algorithms to data set 1 (500 genomes) and data set 2 (4,799 genomes) (see Tables S1 and S2 in the supplemental material). First, we applied both algorithms to data set 1, which was composed of 200 A. baumannii GC1 genomes and 300 A. baumannii non-GC1 genomes. We aimed to predict whether a particular genome in data set 1 belonged to A. baumannii GC1 or not by using SVM and SCM algorithms. Data set 1 was used as input for both algorithms during the training and testing of the models. Once we obtained accurate models that predicted putative biomarker sequences, we used the second A. baumannii genome collection, data set 2 (Table S2). Data set 2 was composed of 312 A. baumannii GC1 genomes and 4,487 A. baumannii non-GC1 genomes. By using blastn searches, we analyzed whether the predicted putative biomarker sequences were also found in data set 2 genomes, maintaining the same pattern found in data set 1. This analysis was done to perform an external validation of the predictions made by both algorithms. The study workflow is summarized in Fig. 1.

10.1128/msystems.00734-22.3 TABLE S1 A. baumannii genomes used as an input data set for SVM algorithm within the data set 1 genome collection. GenBank accession numbers and classification of the 500 genomes in ML analysis are shown. Genomes were classified as A. baumannii GC1 and non-GC1 according to the MLST Pasteur scheme. Download Table S1, PDF file, 0.2 MB. Copyright © 2023 Álvarez et al. 2023 Álvarez et al. https://creativecommons.org/licenses/by/4.0/ This content is distributed under the terms of the Creative Commons Attribution 4.0 International license.

10.1128/msystems.00734-22.4 TABLE S2 A. baumannii genomes used as an input data set for the SVM algorithm within the data set 2 genome collection. Genomes were classified as A. baumannii GC1 and non-GC1 according to the MLST Pasteur scheme. An “–” value in the “ST Pasteur” column indicates that the genome does not yet have an ST assigned in the PubMLST database. Download Table S2, PDF file, 1.8 MB. Copyright © 2023 Álvarez et al. 2023 Álvarez et al. https://creativecommons.org/licenses/by/4.0/ This content is distributed under the terms of the Creative Commons Attribution 4.0 International license.

### Obtaining unique A. baumannii GC1 predictive sequences with SVM.

The Pasteur scheme for MLST has the potential to identify isolates belonging to A. baumannii GC1, providing a neat demarcation of sequence types (STs) composing A. baumannii GC1 and non-GC1 clonal complexes (5, 58–61). Considering this fact, we annotated the ST of each genome in data sets 1 and 2 and categorized them as “GC1” or “non-GC1” (see “MLST classification” in Materials and Methods). We then ran the DBGWAS program using data set 1 genome sequences as input; we obtained a total of 1,622,573 distinct unitigs that represented sequences of diverse length of data set 1, taking into account the genomic variation (62). The length of the unitigs obtained was between 31 and 32,759 bp. We used the variant matrix built by DBGWAS to create the binary matrix used as input for the SVM algorithm. Unitigs that were found in fewer than 50 genomes from data set 1 were discarded; we kept only unitigs that were 31 to 385 bp in size in the input binary matrix.

Parameter tuning and model validations were performed using a 5-fold cross-validation and a grid search over a range of given values to determine the SVM kernel and hyperparameters that generated the best area under the curve (AUC). Once we obtained the SVM model that best fitted the data set 1 genomes, we extracted the first 100 unitigs that contributed most to A. baumannii GC1 strain prediction (Table 1; Table S3) according to the values of the features weight vector. By using blastn, we corroborated if the unitigs predicted by SVM were specific for A. baumannii GC1 detection. For this purpose, we searched for the unitig sequences in the genomes of data set 2 to assess which unitigs had the greatest number of matches within each genome class (A. baumannii GC1 or non-GC1) (Table 2; Table S4). We also annotated the genome location and gene product related to the 100 unitigs (Tables 1 and 3; Tables S3 and S5).

**TABLE 1:** Putative A. baumannii GC1 biomarkers obtained by SVM^*a*^

Unitig ID	Sequence	Length (nucleotides)	Match location	Gene name	Gene product
U1	TTATTCATAGCCTCCTGGATGCGGTAAATCACGGCAACATCAAAAATATGGACTAATGCGGGTAAAATAGCGGCCATTGATTCGCTTGCTCCACCACGGCTTCCCGGTAACGTCACTACTAACGAACGGTCAATAAACCCTGCTACTCCGCGTGACATAGCAGCATATGGCGTGCGTTTTTGTCCAAATGAGCGAGCAGCTTCCATTAAGCCATCTAATTTACGTTCTAAAAGAGGCTCTAGCGTATCAACTGTAATGTCACGCTTACCAATTCCTGTCCCACCCACGGTCTAATGCAAGCATATGATTTGCTCAACTCAAGCACTAAGTTTTTCAAAGTATCCGCTTCATCTGGCAAAATTTGG	367	ABAYE1552:558.0.924	*moaCB*	Bifunctional protein (includes molybdenum cofactor biosynthesis protein C and molybdenum cofactor biosynthesis protein B)
U2	ACCGGCAAAAATTGCAACCCCAATAAAACCGTTCACCCAACCGTTCATAAACTTTTGCATA	61	ABAYE1557:1.0.61	ND^*b*^	Putative permease (drug/metabolite transporter)
U3	ACGTAAGCCATATTGAGAGGCTGCAAGTTCGACACGAGCAACATCACCCAATCGGGTAACC	61	ABAYE1173:777.0.837	ND	Putative multidrug/solvent efflux pump membrane transporter
U4	AAACACTGGGAAATATACTTGAAAAATATCAACAACCCCGAACGTATGCGGGTTGCTCAGT	61	ABAYE3468:844.0.904	ND	Conserved hypothetical protein; putative exported protein
U5	GTATATCTGGCACAAAGCAAAAGGCTTTCATGCCTTACAACATCAACAAACCCGTAAA	58	ABAYE1549:957.0.1014	*moaA*	Molybdopterin biosynthesis, protein A
U6	CATAACCCCTAAAGTACGATGGCTTGATACGGCATCCCACATTGTTAAGCTA	52	ABAYE1636:975.0.1026	*cydB*	Cytochrome *d* terminal oxidase polypeptide subunit II
U7	GAACTATGGCAATACCGATCAGTAAAGATAGAAGTACCTGCCAG	44	ABAYE2207:192.0.235	ND	Putative permease
U8	TCCTTTAGAAAATTTGGTGGGACAAGAAGGCGAAGGTTATA	41	ABAYE1412:651.0.691	ND	Putative acyl-CoA dehydrogenase protein (*acdB*-like)
U9	TCATGTCGAGCTAAGCCAGTACCAGCAAAAGCTGAAG	37	ABAYE1194:1095.0.1131	ND	Putative two-component system sensor histidine kinase
U10	CCCCCCGAACTACATCAATATGATCATATAAGGCC	35	ABAYE1494:441.0.475	ND	Putative outer membrane porin, receptor for Fe(III)-coprogen, Fe(III)-ferrioxamine B, and Fe(III)-rhodotorulic acid uptake (FhuE)

a This table details the unitig identifier (ID), unitig sequence, unitig length (nucleotides), the location where the unitig sequence matched the A. baumannii AYE genome (AN CU459141.1) or A. baumannii AB0057 genome (AN CP001182.2), the gene name corresponding to the genome region matched and the gene product of the first 10 unitigs that contributed most to A. baumannii GC1 genome prediction according to the values of the features weight vector. The A. baumannii AYE strain genome was used as the A. baumannii GC1 reference to locate the unitig sequences. However, when the unitig was not found in the A. baumannii AYE genome, the A. baumannii strain AB0057 genome was used instead. The prefix “REGION” was used when the match occurred either in an intergenic region or in a combination of an intergenic region and a gene. The complete list of the 100 unitigs predicted by SVM is detailed in Table S3 in the supplemental material.

b ND, no data.

**TABLE 2:** Analysis of putative A. baumannii GC1 biomarkers obtained by SVM matches within data sets 1 and 2^*a*^

Unitig ID	Data set 1		Data set 2		*P* value for proportion of GC1 genome matches in data set 1	*P* value for proportion of non-GC1 genome matches in data set 1
	No. of GC1 genomes matching the unitig/total no.	No. of non-GC1 genomes matching the unitig/total no.	No. of GC1 genomes matching the unitig/total no.	No. of non-GC1 genomes matching the unitig/total no.	not differing from proportion of GC1 matches in data set 2	not differing from proportion of non-GC1 matches in data set 2
U1	200/200	0/300	312/312	0/4,487	1	1
U2	200/200	0/300	311/312	0/4,487	1	1
U3	200/200	0/300	311/312	3/4,487	1	1
U4	200/200	0/300	310/312	43/4,487	0.523	0.1106
U5	200/200	0/300	311/312	0/4,487	1	1
U6	200/200	0/300	306/312	1/4,487	0.0862	1
U7	200/200	0/300	309/312	2/4,487	0.2845	1
U8	200/200	0/300	312/312	1/4,487	1	1
U9	200/200	0/300	309/312	5/4,487	0.2845	1
U10	200/200	0/300	311/312	0/4,487	1	1

a This table details the unitig IDs of the first 10 unitigs that contributed most to A. baumannii GC1 genome prediction according to the values of the features weight vector, the number of A. baumannii GC1 and non-GC1 genomes typed by MLST that matched the unitig within data sets 1 and 2 using blastn, and the *P* values of Fisher's exact test using a significance level of 0.05. Fisher’s exact test was calculated in R by considering the nominal variables “data set source” (data set 1 or 2) and “matched” (yes or no). The total number of A. baumannii GC1 and non-GC1 genomes that matched/did not match the unitigs in each data set was used for calculation. The total data of the 100 unitigs predicted by SVM are detailed in Table S4 in the supplemental material.

**TABLE 3:** Rules obtained by the SCM models^*a*^

Rule ID	Rule	Length (nucleotides)	Match location	Gene name	Gene product
R127	Presence (AAAAAAGCATGTTTGAAACATGCTTTTTTATTTTATGGCGTTAAACCAACAGGATTGCGATACCAGCTCTGAATTAGCAAAGCCGCGGCAAAACTATCGGCTGACAACTTCTTGGCACGGCCTTGTT)	127	AYE:REGION:3509175.0.3509301	ND	Putative Holliday junction resolvase
R125	Absence (AATGATTAACAGTACAGGGAAACTAGCAATGAGAAGTTGCATCAAAATGCCTTGACGTTGTGGTGCTGTGCCCTCTACAACAACATTTTGCTTGTTTAAGCTTGGCATAAGTTCAGTGTCTTCAA)	125	ACICU_02924:125.0.249	ND	ATP-dependent Zn protease
R123	Absence (AATGATTAACAGTACAGGGAAACTAGCAATGAGAAGTTGCATCAAAATGCCTTGACGTTGTGGTGCTGTGCCCTCTACAACAACATTTTGCTTGTTTAAGCTTGGCATAAGTTCAGTGTCTTC)	123	ACICU_02924:127.0.249	ND	ATP-dependent Zn protease
R121	Absence (AATGATTAACAGTACAGGGAAACTAGCAATGAGAAGTTGCATCAAAATGCCTTGACGTTGTGGTGCTGTGCCCTCTACAACAACATTTTGCTTGTTTAAGCTTGGCATAAGTTCAGTGTCT)	121	ACICU_02924: 129.0.249	ND	ATP-dependent Zn protease
R119	Absence (AATGATTAACAGTACAGGGAAACTAGCAATGAGAAGTTGCATCAAAATGCCTTGACGTTGTGGTGCTGTGCCCTCTACAACAACATTTTGCTTGTTTAAGCTTGGCATAAGTTCAGTGT)	119	ACICU_02924:131.0.249	ND	ATP-dependent Zn protease
R117	Absence (AACAGTACAGGGAAACTAGCAATGAGAAGTTGCATCAAAATGCCTTGACGTTGTGGTGCTGTGCCCTCTACAACAACATTTTGCTTGTTTAAGCTTGGCATAAGTTCAGTGTCTTCA)	117	ACICU_02924:126.0.242	ND	ATP-dependent Zn protease
R115	Absence (AACAGTACAGGGAAACTAGCAATGAGAAGTTGCATCAAAATGCCTTGACGTTGTGGTGCTGTGCCCTCTACAACAACATTTTGCTTGTTTAAGCTTGGCATAAGTTCAGTGTCTT)	115	ACICU_02924:128.0.242	ND	ATP-dependent Zn protease
R113	Absence (AACAGTACAGGGAAACTAGCAATGAGAAGTTGCATCAAAATGCCTTGACGTTGTGTGCTGTGCCCTCTACAACAACATTTTGCTTGTTTAAGCTTGGCATAAGTTCAGTGTC)	113	ACICU_02924:130.0.242	ND	ATP-dependent Zn protease
R111	Absence (AAGACACTGAACTTATGCCAAGCTTAAACAAGCAAAATGTTGTTGTAGAGGGCACAGCACCACAACGTCAAGGCATTTTGATGCAACTTCTCATTGCTAGTTTCCCTGTAC)	111	ACICU_02924:128.0.238	ND	ATP-dependent Zn protease
R109	Absence (AAGACACTGAACTTATGCCAAGCTTAAACAAGCAAAATGTTGTTGTAGAGGGCACAGCACCACAACGTCAAGGCATTTTGATGCAACTTCTCATTGCTAGTTTCCCTGT)	109	ACICU_02924:128.0.236	ND	ATP-dependent Zn protease

a This table details the rule ID, the rule output from the SCM model, the k-mer length (nucleotides), and the location where the rule sequence matched the A. baumannii AYE genome (AN CU459141.1) or the A. baumannii ACICU genome (AN CP000863.1), and the gene name corresponding to the genome region matched and the gene product of the 10 larger k-mer sequences targeted by the SCM rules. A. baumannii AYE and ACICU genomes were used as A. baumannii GC1 and non-GC1 references, respectively, to locate the k-mer sequences. The prefix “REGION” was used when the match occurred either in an intergenic region or in a combination of an intergenic region and a gene. The complete list of the 49 rules obtained by the SCM models is detailed in Table S5 in the supplemental material. ND, no data.

10.1128/msystems.00734-22.5 TABLE S3 Putative GC1 biomarkers obtained by SVM. This table details the unitig ID, unitig sequence, unitig length (nucleotides), the location where the unitig sequence matched the A. baumannii AYE genome (AN CU459141.1) or A. baumannii AB0057 genome (AN CP001182.2), and the gene name corresponding to the genome region matched and the gene product. The A. baumannii AYE strain genome was used as the A. baumannii GC1 reference to locate the unitig sequences. However, when the unitig was not found in the AYE genome, the A. baumannii AB0057 strain genome was used instead. The prefix “REGION” was used when the match occurred either in an intergenic region or in a combination of an intergenic region and a gene. Download Table S3, PDF file, 0.1 MB. Copyright © 2023 Álvarez et al. 2023 Álvarez et al. https://creativecommons.org/licenses/by/4.0/ This content is distributed under the terms of the Creative Commons Attribution 4.0 International license.

10.1128/msystems.00734-22.6 TABLE S4 Analysis of putative GC1 biomarkers obtained by SVM matches within data sets 1 and 2. This table details the unitig IDs of the first 100 unitigs that contributed most to A. baumannii GC1 genome prediction according to the values of the features weight vector, the number of A. baumannii GC1 and non-GC1 genomes typed by MLST that matched the unitig within data sets 1 and 2 using blastn, and the *P* values of Fisher's exact test using a significance level of 0.05. Fisher’s exact test was calculated in R by considering the nominal variables “data set source” (data set 1 or 2) and “matched” (yes or no). The total number of A. baumannii GC1 and non-GC1 genomes that matched/did not match the unitigs in each data set was used for calculation. Download Table S4, PDF file, 0.09 MB. Copyright © 2023 Álvarez et al. 2023 Álvarez et al. https://creativecommons.org/licenses/by/4.0/ This content is distributed under the terms of the Creative Commons Attribution 4.0 International license.

10.1128/msystems.00734-22.7 TABLE S5 Rules obtained by the SCM models. This table details the rule ID, the rule output from the SCM models, the k-mer length (nucleotides), the location where the rule sequence matched the A. baumannii AYE genome (AN CU459141.1) or the A. baumannii ACICU genome (AN CP000863.1), the gene name corresponding to the genome region matched and the gene product. A. baumannii AYE and ACICU genomes were used as A. baumannii GC1 and non-GC1 references, respectively, to locate the k-mer sequences. The prefix “REGION” was used when the match occurred either in an intergenic region or in a combination of an intergenic region and a gene. Download Table S5, PDF file, 0.06 MB. Copyright © 2023 Álvarez et al. 2023 Álvarez et al. https://creativecommons.org/licenses/by/4.0/ This content is distributed under the terms of the Creative Commons Attribution 4.0 International license.

### Building SCM models for A. baumannii GC1 identification.

In an additional approach, we applied the SCM algorithm implemented in Kover (29). We used as input the k-mers of the 500 A. baumannii genomes contained in data set 1, considering the class label for each genome (A. baumannii GC1 or non-GC1). We ran Kover using k-mer sizes ranging from 31 to 127 nucleotides. Although output rules could be conjunctions (logical-AND) or disjunctions (logical-OR), we obtained 49 simple rules without conjunctions or disjunctions in the models of our study (Table 3; Table S5). Rules depicted the presence (*n* = 10) or absence (*n* = 39) of k-mers in data set 1 genomes. In addition, we registered the number of matches of the k-mer sequences against A. baumannii GC1 and non-GC1 genomes contained in data set 1 (Table 4; Table S6); we also annotated the k-mer sequences (Table 4; Table S6).

**TABLE 4:** Analysis of the SCM rules matches within data sets 1 and 2^*a*^

Rule ID	Data set 1		Data set 2		*P* value for proportion of A. baumannii GC1 genome matches in	*P* value for proportion of A. baumannii non-GC1 genome matches
	No. of A. baumannii GC1 genomes matching the rule/total no.	No. of A. baumannii non-GC1 genomes matching the rule/total no.	No. of A. baumannii GC1 genomes matching the rule/total no.	No. of A. baumannii non-GC1 genomes matching the rule/total no.	data set 1 not differing from proportion of GC1 matches in data set 2	in data set 1 not differing from proportion of non-GC1 matches in data set 2
R127	200/200	4/300	310/312	166/4,487	0.523	0.03405
R125	200/200	1/300	310/312	13/4,487	0.523	0.5964
R123	200/200	1/300	310/312	13/4,487	0.523	0.5964
R121	200/200	1/300	310/312	13/4,487	0.523	0.5964
R119	200/200	1/300	310/312	13/4,487	0.523	0.5964
R117	200/200	1/300	310/312	12/4,487	0.523	0.5693
R115	200/200	1/300	310/312	12/4,487	0.523	0.5693
R113	200/200	1/300	310/312	12/4,487	0.523	0.5693
R111	200/200	1/300	310/312	12/4,487	0.523	0.5693
R109	200/200	1/300	310/312	12/4,487	0.523	0.5693

a This table details the rule IDs of the 10 larger k-mer sequences targeted by the SCM rules, the number of A. baumannii GC1 and non-GC1 genomes typed by MLST that matched the rule within data sets 1 and 2 by using blastn, and the *P* values of Fisher's exact test using a significance level of 0.05. Fisher’s exact test was calculated in R by considering the nominal variables “data set source” (data set 1 or 2) and “matched” (yes or no). The total number of A. baumannii GC1 and non-GC1 genomes that matched/did not match the rules in each data set was used for calculation. The total data of the 49 SCM rules are detailed in Table S6 in the supplemental material.

10.1128/msystems.00734-22.8 TABLE S6 Analysis of the SCM rule matches within data sets 1 and 2. This table details the rule IDs of the 100 larger k-mer sequences targeted by the SCM rules, the number of A. baumannii GC1 and non-GC1 genomes typed by MLST that matched the rule within data sets 1 and 2 using blastn, and the *P* values of Fisher's exact test using a significance level of 0.05. Fisher’s exact test was calculated in R by considering the nominal variables “data set source” (data set 1 or 2) and “matched” (yes or no). The total number of A. baumannii GC1 and non-GC1 genomes that matched/did not match the rules in each data set was used for calculation. Download Table S6, PDF file, 0.05 MB. Copyright © 2023 Álvarez et al. 2023 Álvarez et al. https://creativecommons.org/licenses/by/4.0/ This content is distributed under the terms of the Creative Commons Attribution 4.0 International license.

We observed that the rules obtained by the SCM models selected fragments of different lengths that matched the loci ACICU_02924 (*n* = 23), ACICU_02095 (*n* = 5), ABAYE3455 (*n* = 5), ACICU_01506 (*n* = 2), and ABAYE2468 (*n* = 2) (Table 3; Table S5). This fact could be caused by point mutations contained in the loci that the SCM models associated with A. baumannii GC1 prediction as previously reported by Kover developers (29).

### Selection of candidate biomarkers for rapid detection of A. baumannii GC1.

We were interested in obtaining the longest DNA sequences shared by all A. baumannii GC1 genomes and, at the same time, without matches among A. baumannii non-GC1 genomes. For this purpose, unitigs and k-mer sequences obtained as candidate biomarkers for A. baumannii GC1 using the SVM and SCM algorithms were sorted in descending order according to sequence length. Then, we considered the number of A. baumannii GC1 genomes that matched the unitig sequence or the SCM rule and sorted the candidate sequences in ascending order according to the number of A. baumannii non-GC1 genome matches.

**FIG 2:**
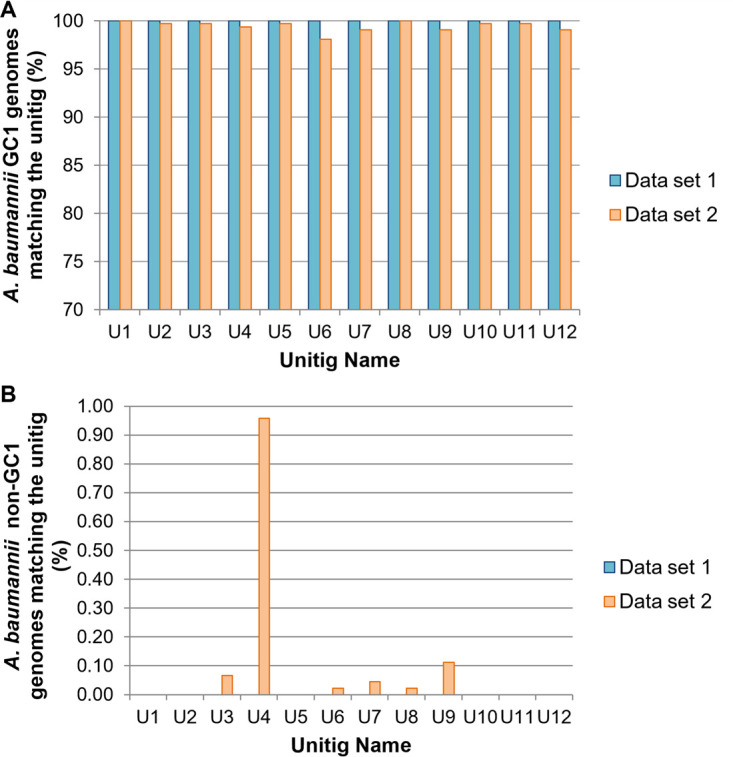
Percentages of genomes in data set 1 and data set 2 matching unitig sequences U1 to U12. (A) A. baumannii GC1 genomes; (B) A. baumannii non-GC1 genomes.

First, we analyzed the SVM results. Data related to the unitigs obtained are listed in Tables 1 and 2 and Fig. 2 and also in Tables S3 and S4 in the supplemental material. We named unitigs from U1 to U100. We observed that the unitigs named U1 to U12 were found in 100% of A. baumannii GC1 genomes while they were not found in A. baumannii non-GC1 genomes from data set 1. However, U1 was the only unitig found in 100% of the A. baumannii GC1 genomes within data sets 1 and 2 and absent in A. baumannii non-GC1 genomes of both data sets, being a specific biomarker for A. baumannii GC1 identification. Despite the fact that the U8 sequence was found in 100% of A. baumannii GC1 genomes within data sets 1 and 2, it was also found in 1/4,487 of A. baumannii non-GC1 genomes from data set 2. The U8 sequence matched the A. baumannii AYE genome in locus tag ABAYE1412 between coordinates 651 and 691 (Table 1; Table S3). The fragment is part of a gene that encodes a putative acyl coenzyme A (acyl-CoA) dehydrogenase protein (*acdB*-like). As the U8 sequence was not found exclusively in A. baumannii GC1 genomes, we discarded it as a possible biomarker of A. baumannii GC1. Among these 12 unitigs, the U4 sequence was the one that had a higher number of matches (43/4,487) with A. baumannii non-GC1 genomes from data set 2.

**FIG 3:**
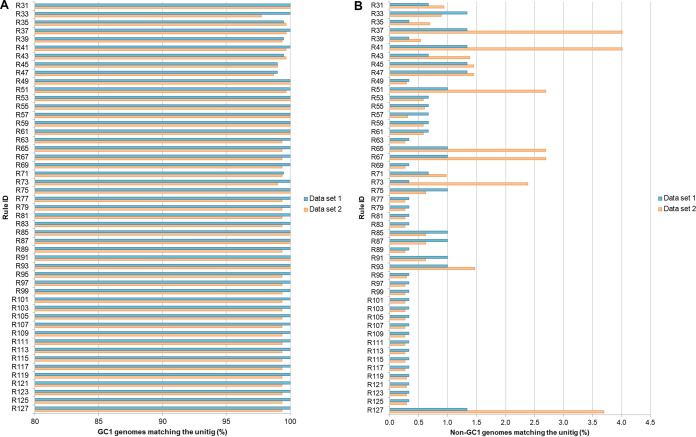
Percentages of genomes in data set 1 and data set 2 matching the SCM rules. (A) A. baumannii GC1 genomes; (B) A. baumannii non-GC1 genomes. We observed that within the 43 rules which targeted 100% of A. baumannii GC1 genomes from data set 1, two set of rules targeted only one non-GC1 genome from the same data set. Rules R125, R123, R121, R119, R117, R115, R113, R111, R109, R107, R105, R103, R101, R99, R97, R95, R89, R83, R81, R79, R77, R73, R69, R63, R39 and, R35 targeted the genome with AN GCF_000248195.1 (ST69) and R49 targeted the genome with AN GCF_000453745.1 (ST2). Regarding data set 2, rules R93, R91, R87, R85, R75, R61, R59, R57, R55, R53, R49, and R31 targeted 100% of A. baumannii GC1 genomes but also targeted several A. baumannii non-GC1 genomes from data set 2 (66/4,487, 28/4,487, 28/4,487, 28/4,487, 28/4,487, 26/44,87, 26/4,487, 14/4,487, 27/4,487, 26/4,487, 13/4,487, and 42/4,487 genomes, respectively).

The SCM results are shown in Tables 3 and 4 and Fig. 3 as well as in Tables S5 and S6. We observed that 43/49 rules targeted 100% of A. baumannii GC1 genomes from data set 1 but also targeted between 1 and 4 A. baumannii non-GC1 genomes from data set 1. Within these 43 rules, two sets of rules targeted only 1 A. baumannii non-GC1 genome from data set 1; as an example of this, 26/49 rules targeted a genome with accession no. (AN) GCF_000248195.1 (ST 69) and 1/49 rules (R49) targeted a genome with AN GCF_000453745.1 (ST 2) (Fig. 3). Concerning data set 2, 12/49 rules targeted 100% of A. baumannii GC1 genomes but also targeted several A. baumannii non-GC1 genomes from data set 2. Since no rule obtained can uniquely identify A. baumannii GC1 genomes contained in our data sets, we were unable to obtain a putative biomarker from the results of the SCM models.

When using data set 2 to validate the results obtained by the SVM and SCM models from data set 1, we observed that in the case of the SVM classifier, there was no statistically significant difference (*P* > 0.05) in the number of matches of the unitigs regardless of the genome data set (data set 1 or 2) except for unitig U69 (Table 2; Table S4). In the case of U69, we found a significant difference between the numbers of matches with A. baumannii non-GC1 genomes from data sets 1 and 2 (*P* = 0.01077). Concerning the SCM classifier, we observed that there was no statistically significant difference in the number of A. baumannii GC1 and non-GC1 genomes from data sets 1 and 2 that matched the rules (*P* > 0.05) (Table 4; Table S6). As we mentioned, the SVM model predicted the U1 sequence as a specific biomarker for A. baumannii GC1 genomes. The U1 sequence had 367 nucleotides and matched the A. baumannii AYE genome in locus tag ABAYE1552 between coordinates 558 and 924 (Table 1; Table S3). This region corresponds to a fragment of the *moaCB* gene, which encodes a bifunctional protein that includes molybdenum cofactor biosynthesis protein C and protein B (63). In particular, the U1 sequence matched the region of the *moaCB* gene, which encodes MoaB protein. The molybdenum cofactor (Moco) is an essential component of a large family of enzymes involved in carbon, nitrogen, and sulfur metabolism whose biosynthetic pathway is evolutionarily conserved. The MoaC protein, together with the MoaA protein, is involved in the first step of Moco biosynthesis (63). Interestingly, various studies have linked in-host survival of prevalent pathogenic bacteria such as Mycobacterium tuberculosis, Escherichia coli, and Salmonella enterica to the presence of functional molybdoenzymes (64–67). In our study, we found that the U1 sequence was 100% conserved in A. baumannii GC1 genomes (no single nucleotide polymorphisms [SNPs]), while it was a variable region with 1 to 74 SNPs in a total of 4,987 A. baumannii non-GC1 genomes from data sets 1 and 2, rendering 94 allelic variants in A. baumannii non-GC1 genomes. According to these results, the sequence of the *moaCB* gene comprising between 558 and 924 nucleotides allowed accurate discrimination for A. baumannii GC1 and non-GC1 genomes, becoming a biomarker that differentiates the two groups. Moreover, we observed that the U1 sequence is conserved in all the A. baumannii GC1 genomes, but this is not the case for A. baumannii GC2. For this reason, the U1 sequence could not be used as a biomarker for accurate detection of A. baumannii GC2 strains.

Since 95 variants within 5,299 A. baumannii strains were identified with multiple SNPs along the entire sequence of U1, we proposed that a strategy based on PCR amplification would allow us to accurately differentiate A. baumannii GC1 from non-GC1 strains. For this reason, we designed the primer pair BioM_GC1_ABA F (5′-TATTCATAGCCTCCTGGATGC-3′) and BioM_GC1_ABA R (5′-CCAGATGAAGCGGATACTTTG-3′), with coordinates 559 to 914 from the ABAYE1552 locus tag representing 356 bp of the U1 sequence (positions 2 to 357). By a blastn search, we identified that this primer pair amplified only the U1 sequence recognized in A. baumannii GC1 strains among all the variants detected so far in non-GC1 genomes. The closest variants showed a mismatch of 1 nucleotide at the 3′ end of the reverse primer in A. baumannii ST163, ST411, and ST976 (AN GCF_015537765, GCF_000453725, and GCF_010500415, respectively).

Experimental analysis, with a total of 35 A. baumannii strains, i.e., 10 A. baumannii GC1 strains and 25 A. baumannii non-GC1 strains, including 4 ST2 (GC2), 15 ST79, 5 ST119, and 1 ST404, was performed to test the primer pair BioM_GC1_ABA F and BioM_GC1_ABA F (Fig. S1). A. baumannii GC1 strains that had been isolated more than 25 years apart, such as A144 (1997), A155 (1994), and HAX25Aba (2021), were tested (68, 69). The pair of primers designed in this study amplified by PCR only the A. baumannii GC1 strains, and as expected, the subsequent DNA sequence analysis of the amplicon showed 100% identity and query coverage with 356 bp of U1, as confirmed by Clustal alignment (Fig. S2). Interestingly, A. baumannii Ab103 ST119, which has two mismatches in each primer, i.e., at positions 15 and 21 of the 21 nucleotides of BioM_GC1_ABA F and at positions 9 and 21 of the 21 nucleotides of BioM_GC1_ABA R, also rendered a negative result in this PCR’s conditions (see below). This result confirmed the specificity of this pair of primers.

10.1128/msystems.00734-22.1 FIG S1 A. baumannii GC1 biomarker identification by PCR. PCR amplification of several A. baumannii strains is shown. The sample order at the upper wells corresponds to ladder, A144 ST1, A155 ST1, HAX19AbA ST1, HAX26Aba ST2, HAX28Aba ST2, A118 ST404, A110 ST1, A185 ST1, A325 ST119, A376 ST119, A103 ST119, and ladder. The sample order at the lower wells corresponds to ladder, HAX22Aba ST1, HAX25Aba ST1, HAX27Aba ST1, Ab33405 ST79, Ab66 ST79, 186 ST79, F33943 ST79, A171 ST79, A177 ST79, A182 ST79, A384 ST79, negative control, and ladder. Agarose gel 1.5%, electrophoresis in Tris-acetate-EDTA (TAE) buffer at 80 V for 45 min. One-kilobase-pair DNA ladder (catalog no. K0178; Inbio Highway, Argentina). Download FIG S1, TIF file, 1.5 MB. Copyright © 2023 Álvarez et al. 2023 Álvarez et al. https://creativecommons.org/licenses/by/4.0/ This content is distributed under the terms of the Creative Commons Attribution 4.0 International license.

10.1128/msystems.00734-22.2 FIG S2 Alignment of the DNA sequence of U1 identified in A. baumannii strains tested by PCR. Shown are the primer pairs used in the PCR which target coordinates 559 to 914 from locus tag ABAYE1552 representing positions 2 to 357 of the 367-bp-long U1 sequence identified in A. baumannii GC1 amplified by PCR. Sequence types (ST) according to Pasteur’s MLST schemes and global clones (GCs) are indicated in brackets. An asterisk indicates that the ST does not belong to a GC. Download FIG S2, TIF file, 1.0 MB. Copyright © 2023 Álvarez et al. 2023 Álvarez et al. https://creativecommons.org/licenses/by/4.0/ This content is distributed under the terms of the Creative Commons Attribution 4.0 International license.

The optimum PCR amplification was established in a final volume of 25 μL containing 0.625 U of GoTaq DNA polymerase (catalog no. M3005; Promega, USA), 5 μL of 5× Green GoTaq buffer with MgCl_2_ at a final concentration of 1.5 mM in the 1× reaction mixture, 0.4 pM each deoxynucleotide triphosphate (dNTP), 1 μM each primer, and 5 μL of DNA from boiling 3 or 4 A. baumannii colonies in 100 μL of sterile H_2_O. DNA of A. baumannii GC1 strain A144 and water were used as positive and negative controls, respectively. The PCR cycling conditions consisted of an initial denaturation at 94°C for 5 min, followed by 30 cycles of denaturation at 94°C for 45 s, annealing at 58°C for 45 s, and extension at 72°C for 30 s, and then a final extension at 72°C for 5 min. Alternatively to this PCR to identify A. baumannii GC1, TaqMan assays could also provide a molecular typing method capable of being translated into clinical settings to differentiate A. baumannii GC1 and other relevant GCs or STs.

### Evaluation of the performance of the SVM and SCM models.

To avoid overfitting, a 5-fold cross-validation was performed with the training data sets used as input in both the SVM and SCM algorithms to select the best hyperparameter values with the highest AUC. Performance during testing was evaluated using the best hyperparameters obtained in terms of sensitivity, specificity, accuracy, precision, and F1 score (Tables 5 and 6; Table S7). The sensitivity of the SVM model was 1 ± 0.00, indicating that 100% of A. baumannii GC1 genomes were correctly identified within the testing data set. Also, the SVM model achieved a high specificity (1 ± 0.00) when predicting A. baumannii non-GC1 genomes from the testing data sets. The precision value was 1 ± 0.00 when the model predicted that a genome was within A. baumannii GC1, being correct 100% of the time. Accuracy was 1 ± 0.00, indicating that 100% of A. baumannii GC1 and non-GC1 genomes were correctly predicted. No false positives and no false negatives were predicted by the SVM model.

**TABLE 5:** Prediction metrics on test data set partitions from data set 1 using the best-performing SVM model^*a*^

Metric	Value
ACC	1.00 ± 0.00
SENS	1.00 ± 0.00
SPE	1.00 ± 0.00
PRE	1.00 ± 0.00
F1	1.00 ± 0.00
No. of TP	52
No. of TN	48
No. of FP	0
No. of FN	0
No. of isolates typed by MLST	
GC1	52
Non-GC1	48
Correlation of MLST typing to the model prediction	*P* < 2.2e^−16^

a The correlation between MLST typing and model prediction was calculated using Fisher’s exact test in R using a significance level of 0.05. The nominal variables “MLST typing” and “prediction” were considered during Fisher’s exact test calculation. The variable “MLST typing” represented the genomes typed as A. baumannii GC1 (positive label) or non-GC1 (negative label) by MLST technique (true class). On the other hand, the variable “prediction” represented the genomes predicted to be A. baumannii GC1 or non-GC1 by the SVM model (predicted class). We used the number of true positives (TP), false positives (FP), false negatives (FN), and true negatives (TN) in a 2 × 2 contingency table. The null hypothesis used to evaluate the correlation between MLST typing and model prediction was as follows: true class (MLST typing) and predicted class are independent, knowing that the value of one variable does not help to predict the value of the other variable. ACC, accuracy; SENS, sensitivity; SPE, specificity; PRE, precision; F1, F1 score; TP, true positives; TN, true negatives; FP, false positives; FN, false negatives.

**TABLE 6:** Prediction metrics on test data set partitions from data set 1 using the best-performing SCM models^*a*^

Rule ID	SENS	SPE	PRE	ACC	F1	No. of TP	No. of TN	No. of FP	No. of FN	No. of isolates typed by MLST		Correlation of MLST typing to the model prediction (*P* value)
										GC1	non-GC1	
R127	1.00	0.96	0.95	0.98	0.97	72	91	4	0	72	95	2.2e^−16^
R125	1.00	0.99	0.99	0.99	0.99	72	94	1	0	72	95	2.2e^−16^
R123	1.00	0.99	0.99	0.99	0.99	72	94	1	0	72	95	2.2e^−16^
R121	1.00	0.99	0.99	0.99	0.99	72	94	1	0	72	95	2.2e^−16^
R119	1.00	0.99	0.99	0.99	0.99	72	94	1	0	72	95	2.2e^−16^
R117	1.00	0.99	0.99	0.99	0.99	72	94	1	0	72	95	2.2e^−16^
R115	1.00	0.99	0.99	0.99	0.99	72	94	1	0	72	95	2.2e^−16^
R113	1.00	0.99	0.99	0.99	0.99	72	94	1	0	72	95	2.2e^−16^
R111	1.00	0.99	0.99	0.99	0.99	72	94	1	0	72	95	2.2e^−16^
R109	1.00	0.99	0.99	0.99	0.99	72	94	1	0	72	95	2.2e^−16^

a Correlation between MLST typing and model prediction was calculated using Fisher’s exact test in R using a significance level of 0.05. The nominal variables “MLST typing” and “prediction” were considered during Fisher’s exact test calculation. The variable “MLST typing” represented the genomes typed as A. baumannii GC1 (positive label) or A. baumannii non-GC1 (negative label) by the MLST technique (true class). On the other hand, the variable “prediction” represented the genomes predicted to be A. baumannii GC1 or non-GC1 by the SCM model (predicted class). We used the number of true positives (TP), false positives (FP), false negatives (FN), and true negatives (TN) in a 2 × 2 contingency table. The null hypothesis used to evaluate the correlation between MLST typing and model prediction was as follows: true class (MLST typing) and predicted class are independent, knowing that the value of one variable does not help to predict the value of the other variable. The total metrics of the 49 rules obtained by the SCM models are detailed in Table S7 in the supplemental material. ACC, accuracy; SENS, sensitivity; SPE, specificity; PRE, precision; F1, F1 score; TP, true positives, TN, true negatives; FP, false positives; FN, false negatives.

10.1128/msystems.00734-22.9 TABLE S7 Prediction metrics on test data set partitions from data set 1 using the best-performing SCM models. The correlation between MLST typing and model prediction was calculated using Fisher’s exact test in R using a significance level of 0.05. The nominal variables “MLST typing” and “prediction” were considered during Fisher’s exact test calculation. The variable “MLST typing” represented the genomes typed as A. baumannii GC1 (positive label) or A. baumannii non-GC1 (negative label) by the MLST technique (true class). On the other hand, the variable “prediction” represented the genomes predicted to be A. baumannii GC1 or non-GC1 by the SCM model (predicted class). We used the number of true positives (TP), false positives (FP), false negatives (FN), and true negatives (TN) in a 2 × 2 contingency table. The null hypothesis used to evaluate the correlation between MLST typing and model prediction was as follows: true class (MLST typing) and predicted class are independent, knowing that the value of one variable does not help to predict the value of the other variable. Download Table S7, PDF file, 0.1 MB. Copyright © 2023 Álvarez et al. 2023 Álvarez et al. https://creativecommons.org/licenses/by/4.0/ This content is distributed under the terms of the Creative Commons Attribution 4.0 International license.

Regarding the SCM models, the mean values of sensitivity, specificity, precision, and accuracy were 1 ± 0.01, 0.98 ± 0.01, 0.97 ± 0.01, and 0.99 ± 0.01, respectively. The mean rate of false positives was 1.11%, while the mean rate of false negatives was 0.097%. All the rules obtained by the SCM models predicted false positives within the testing data set (Table 6).

**FIG 4:**
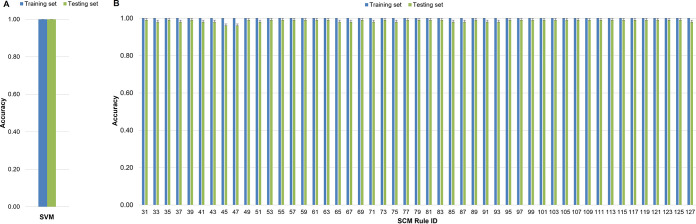
Mean accuracies of the SVM and SCM models. Blue bars represent the mean accuracy of the models for the training data set. Green bars represent the accuracy of the models for the test data set. The models were run with the best hyperparameters selected from a 5-fold cross-validation. The error bars indicate standard deviations. (A) SVM model; (B) SCM models.

The training and the testing accuracies of the SVM and SCM models were above 0.99 (Fig. 4), indicating that the SVM and SCM models were not overfitted. The F1 score, which is the harmonic mean of precision and recall that is commonly used to compare different classification algorithms, was similarly high in the SVM and SCM models (1.00 and 0.99 ± 0.01, respectively). We used Fisher's exact test to evaluate the performance of the SVM and SCM predictions, comparing the actual genome classes (A. baumannii GC1 and non-GC1) typed by MLST and the predicted classes obtained by the models. In both cases, we obtained a *P* of <2.2e^−16^ (Tables 5 and 6; Table S7), indicating that the SVM and SCM models could significantly classify A. baumannii GC1 and non-GC1 strains. Despite these results, since the aim of this work was to find a biomarker that uniquely identifies A. baumannii GC1 genomes, the SVM model performed better than the SCM models since it did not predict false positives or false negatives.

## DISCUSSION

High-risk clones, also called “superbugs,” are dangerous clonal complexes with epidemic behavior equipped with exceptional resources both to infect the host and to evolve to extreme drug resistance phenotypes over time in the nosocomial niche (1, 70–76). A molecular understanding of the genetic and/or transcriptomic traits that lead to these capabilities is still unknown (77, 78). Our study showed that ML applied to the study of high-risk clones can not only help in the identification of thoroughly accurate biomarkers but also contribute to disentangling molecular pathways that lead to epidemic lineages in the nosocomial niche which have not yet been completely deciphered. Accordingly, these findings could be used as therapeutic targets to reduce the dissemination of lineages with epidemic behavior. This is the case of the U1 sequence identified in the present study, which corresponded to 367 bp of the *moaCB* gene encoding a bifunctional protein that includes molybdenum cofactor biosynthesis protein C and protein B (63). Mononuclear molybdoenzymes (Mo enzymes) occur in organisms in all domains of life, where they mediate essential cellular functions such as energy generation and detoxification reactions (79). It has been shown that in bacterial pathogens, several processes such as molybdate uptake, cofactor biosynthesis, and the activities of Mo enzymes affect fitness in the host as well as virulence (79). In addition to many studies on Mo enzymes that identified their crucial role in pathogenic species such as E. coli, S. enterica, Campylobacter jejuni, and Mycobacterium tuberculosis, some reports recently identified that these enzymes also contribute to the survival of ESKAPE pathogens (65, 80–84). Experimental studies must be undertaken to investigate the role of the *moaCB* gene in the virulence and fitness of A. baumannii, which is also included in the ESKAPE group. Based on our results and previous experimental data in other pathogenic species (79), we can hypothesize that the U1 fragment of the *moaCB* gene in A. baumannii GC1 may be related to an essential metabolic pathway that plays a vital role in the maintenance of epidemic clones in the hospital environment. Since it has been found that most of the Mo enzymes belong to groups that are unique to prokaryotes, these have been proposed as promising targets for the development of new antibiotic agents (79).

Given the variability observed in biomarker U1 between A. baumannii GC1 and the 94 variants in A. baumannii non-GC1 strains, we propose a simple molecular biology strategy of one-step PCR amplification to accurately differentiate A. baumannii GC1 from non-GC1 strains without performing MLST, or WGS plus comparative genomics. The strategy proposed here, which was experimentally tested in this study, is accessible to a wide range of clinical and/or research laboratories. In addition, as the methodology consists of a single PCR, the detection of A. baumannii GC1 strains can be performed from the colony or directly from the clinical sample, giving the possibility of an early and simple diagnosis of this lineage.

Due to the increasing availability of bacterial WGS data, very active research has emerged on the use of this tool for genotype-phenotype prediction of antibiotic susceptibility (29, 32, 33, 43, 53, 85–87); however, there are no data available concerning the identification of high-risk clones based on WGS data excluding MLST. Previous PCR-based studies used *bla*_OXA-51_ as one of the three targeted genes to discriminate strains of A. baumannii GC1, GC2, and GC3 (18, 19), and the detection of a deletion of 108 bp in the 5′ conserved segment (5′-CS) of the class 1 integron has also been proposed for identification of A. baumannii GC1 strains (19). Since *bla*_OXA-51_ has been found in other species (88–90) and the deletion of 108 bp as a biomarker partially differentiates two lineages within A. baumannii GC1 (7) and considering that both can be subjected to lateral genetic transfer events, the analysis provided by ML in the present study supports a more accurate and solid tool to evaluate the presence of A. baumannii GC1 strains. Accordingly, U1 is part of an essential gene in the A. baumannii GC1 genome (*moaCB*). This suggests that the optimization of metabolic genes from the core genome may be related to the exceptional abilities of high-risk clones. Interestingly, our data suggest that the accessory genome such as genomic islands or transposons involved in pathogenicity or antibiotic resistance, at least in A. baumannii GC1 strains, would not have played a causal role in the adaptation of this lineage to the hospital niche over time (1).

In our work, we applied two ML algorithms that differ substantially in methodology. The SVM algorithm used the number of unitigs occurring in A. baumannii GC1 and A. baumannii non-GC1 genomes to learn and predict clonal membership of the strains, while the SCM algorithm used a greedy approach to construct conjunction or disjunction rules to find the most concise set of k-mers that allows for accurate A. baumannii GC1 or A. baumannii non-GC1 genome prediction. Previously, methods that combined the use of the SVM and SCM algorithms and the representation of genomic data as k-mers were used to find genomic biomarkers to identify antibiotic resistance (29) or to predict antibiotic resistance from WGS data (53, 85). We ran the SCM algorithm through the Kover program with k-mer lengths between 31 and 127 nucleotides to be able to analyze all the possible rules obtained from these k-mer lengths. Also, we ran the SVM algorithm using unitigs that usually corresponded to a longer sequence than that of the individual equivalent k-mers. Unitigs are defined as the longest sequences that can be obtained when k-mers overlap by exactly k-1 nucleotides (62, 87, 91). In k-mer-based genome representations, the main downside is that the representation contains a lot of redundancy, since many k-mers are always present or absent simultaneously (e.g., gene deletion/insertion). In this sense, it has been proposed that k-mers be replaced with unitigs (62, 87, 91).

Although the best model that fitted our data was a linear SVM model, we evaluated linear, polynomial, radial basis function, and sigmoid SVM kernels. Other genome-wide association studies (GWAS) tools that apply ML techniques to the prediction of phenotypes from genotype data, such as Pyseer (92) or PhenotypeSeeker (93), use different approaches to find the best models to solve classification problems. Pyseer uses generalized linear models (GLiM), a linear regression model, and PhenotypeSeeker uses a logistic regression (LR) model. While GLiM performs a more simplistic linear regression using a set of observed values to predict its response variable, SVM deploys much more sophisticated techniques (57, 94, 95). SVM can perform kernel tricks that can handle nonlinear data, thus making the nonlinear data appear to be linear. These tricks cannot be done by GLiM or LR (39, 48, 96). Moreover, it has been shown that when it is of interest to predict the group to which a new observation belongs, based on a single variable, SVM models are a feasible alternative to LR since SVM models require fewer variables to perform better than or as well as LR (97, 98). In addition, the risk of overfitting is less in SVM, while LR is vulnerable to overfitting (99). In comparison with Pyseer and PhenotypeSeeker approaches, our methodology considered the testing of different SVM kernels and allowed the possibility of using linear or complex nonlinear functions to find the best model. On the other hand, Pyseer and PhenotypeSeeker perform weighting of strains by using a distance matrix of the strains to account for population structure (92, 93). Although the implementation of our algorithm did not include prior knowledge of population structure, we could successfully find a specific biomarker for A. baumannii GC1 strains.

We also proved by using statistical methods that the SVM and SCM models could significantly classify A. baumannii GC1 isolates (*P* < 0.05). While the SVM classifier predicted the U1 sequence as a specific biomarker for A. baumannii GC1 genomes, none of the rules obtained with the SCM models was able to uniquely identify A. baumannii GC1 genomes. All the rules obtained by the SCM models matched in A. baumannii GC1 and A. baumannii non-GC1 genomes from both data sets 1 and 2. Due to this result, it was not possible to obtain a sequence that could be used as a specific biomarker for A. baumannii GC1 strains from the rules obtained by the SCM models. A key step for the successful implementation of ML algorithms is the preparation of the input data sets (57, 100–102). In our study, we faced two issues related to the preparation of data sets 1 and 2. On the one hand, a limitation in the program DBGWAS during the preparation of the matrix used in the SVM algorithm meant that only a total of 500 genomes could be included in the training data set. Since our goal was to use the same training data set for the SVM and SCM models, the 500 genomes were also used as input in the SCM algorithm. On the other hand, the scarcity of A. baumannii GC1 genomes available in GenBank in comparison to A. baumannii non-GC1 genomes caused data set 2 to have visibly more genomes representing the A. baumannii non-GC1 class. Despite this fact, the results obtained from data sets 1 and 2 remained consistent in 100% (*P* > 0.05) of the cases for the SVM model and more than 91.83% (*P* > 0.05) of the cases for the SCM models. Perhaps the limitation in the number of genomes in the training data set is one of the reasons why the SCM models did not have enough samples to learn from A. baumannii GC1 genomes and therefore could not find a rule that uniquely identifies them. Conversely, the numbers of isolates predicted to be A. baumannii GC1 or non-GC1 by the SVM model using unitig U1 were the same as the result obtained by the MLST technique. This fact indicated that the SVM model obtained was excellent in the classification of A. baumannii GC1 and non-GC1 genomes. It will be interesting to study in future works how the data set 1 splitting strategy (unitigs or k-mers), the number of genomes of each class (A. baumannii GC1 and non-GC1) in data set 1, and the total number of A. baumannii genomes in data set 1 impact the SVM and SCM model predictions and performances. One possible approach could be using as input the binary matrix obtained from the unitigs representing data set 1 genomes generated by the program DBGWAS in the program Kover and then to analyze the SCM model results. In the same way, we could obtain k-mer profiles (k-mer sizes ranging from 31 to 127 nucleotides) from data set 1 genomes and the k-mer matrixes associated with each profile using the DSK k-mer counter (103). DSK is used by Kover to internally compute k-mer profiles from the input genomes (104). Then, we could use the k-mer matrixes as input in the SVM algorithm. As result, we would obtain new putative sequence biomarkers from both approaches, and we could compare them with the ones obtained in the current work.

In conclusion, these results suggest that the application of ML to identify biomarkers for high-risk clones or superbugs can also be used at an exploratory level of great precision since it can be useful for novel understandings related to bacterial adaptation within the nosocomial niche. In turn, these data can contribute to experimental work with the possibility of further translation to clinical settings. The SVM algorithm made genetic predictions based on the presence or absence of short genomic sequences in both A. baumannii GC1 and non-GC1 genomes. It detected a biomarker, U1, which is unrelated to lateral genetic transfer, accessory genome, or antibiotic pressures, and can uniquely identify the CG1 strains. The identification of this biomarker by the SVM algorithm, in agreement with previous experimental works on the group of Mo enzymes, showed that the application of ML could be a powerful tool to discover new therapeutic targets for the development of new antibiotic agents.

## MATERIALS AND METHODS

### MLST classification.

Multilocus sequence typing (MLST) of all genomes in the data collection was performed *in silico* using the mlst software developed by T. Seemann (https://github.com/tseemann/mlst) and Pasteur’s MLST database and schema for A. baumannii (https://pubmlst.org/organisms/acinetobacter-baumannii). A. baumannii sequence type (ST) numbers ST1, ST19, ST20, ST81, ST94, ST328, ST460, ST623, ST315, ST717, and ST1106 were classified into A. baumannii GC1 and other STs into A. baumannii non-GC1 genomes (see Tables S1 and S2 in the supplemental material).

### Data collection.

To perform an accurate ML analysis to identify an A. baumannii GC1 biomarker, we defined two data sets to do our studies.

Data set 1 was composed of 200 A. baumannii GC1 genomes and 300 A. baumannii non-GC1 genomes obtained from GenBank and typed by MLST as previously described. These genomes were retrieved from the GenBank assembly database by filtering with “Acinetobacter baumannii” in the search-by-organism option (https://www.ncbi.nlm.nih.gov/assembly/organism/; last accessed July 2021). A. baumannii GC1 genomes included 18 genomes used in a previous study (1) and 182 A. baumannii genomes as scaffolds and contigs (Table S1). A. baumannii non-GC1 genomes included five genomes belonging to other high-risk epidemic clones such as ACICU (CP031380.1) as representative of GC2, Naval-13 (AMDR01000001.1) as representative of GC3, AB33405 (NZ_JPXZ00000000.1) as representative of local epidemic clone CC113, and both ATCC 17978 (CP018664.1) and A118 (AEOW01000000) as sporadic clones. We also included 205 A. baumannii non-GC1 genomes as scaffolds and contigs (Table S1). Data set 2 was composed of 312 A. baumannii GC1 genomes and 4,487 A. baumannii non-GC1 genomes (Table S2) retrieved from the GenBank assembly database (last accessed July 2021). The number of genomes in these data sets was limited by the A. baumannii GC1 and non-GC1 genomes available in the GenBank database at the time of the query. We used data set 2 to validate using blastn searches the results obtained by the SVM model (Table S2). STs were numbered according to the Pasteur scheme for MLST (Tables S1 and S2).

### Machine learning analyses.

The SVM classifier is based on the maximization of the margin around the hyperplane (*w^T^x + b*) separating samples or instances of the different classes (56, 105). Each instance *i *= 1, …, *m* consists of an N-dimensional feature vector *x_i_* and a class label, $y_{i}\in \left\{+\text{1},\;-\text{1}\right\}$. The maximization of the margin corresponds to the following minimization:

$$w^{*},b^{*},\;\xi^{\text{*}}\;=\;\arg\;\underset{w,b,\xi }{\min}\frac{1}{2}\;\|w{\|}^{2}\;+C\overset{m}{\underset{i=1}{\sum }}\xi_{i}$$

$$\text{s.t.}\;{\text{ y}}_{i}\left(w^{T}x_{i}\;+\;b\right)\;\geq \;1\;-\;\xi_{i};\;\xi_{i}\;\geq \;0;\;i=1,\;\ldots ,m$$

In this soft-margin SVM equation, ξ*_i_* is a penalty for misclassification or classification within the margin. Parameter *C* sets the weight of this penalty. The resulting weight vector w* encodes the contributions of all features to the classifier (56). b* refers the resulting bias term. The bias term shifts the hyperplane away from the origin and allows the SVM to fit a hyperplane that is not necessarily passing through the origin. ξ* refers to the resulting slack variable.

We created a Python script using scikit-learn (106) to run the SVM algorithm. The script evaluated the classifier through a 5-fold cross-validation. In detail, the data were split into five consecutive folds (without shuffling) using the Python scikit-learn (sklearn) KFold function (https://scikit-learn.org/stable/modules/cross_validation.html#k-fold), and five models were built. Each fold was used once as a test set, while the four remaining folds formed the training set. During each of the five iterations, hyperparameter tuning was done using a 5-fold cross-validated grid search using the GridSearchCV function implemented in the sklearn.model_selection package (https://scikit-learn.org/stable/modules/generated/sklearn.model_selection.GridSearchCV.html#sklearn.model_selection.GridSearchCV) to find the best hyperparameters. We evaluated linear, polynomial (with a default degree of 3), radial basis function (RBF), and sigmoid kernels. We considered values between 0.01 and 100 for the penalty parameter of the error term (*C*) and values between 0.000001 and 10 for the gamma parameter. The predictions of all five iterations were compared using the AUC score (https://scikit-learn.org/stable/modules/model_evaluation.html#roc-metrics). Finally, we built up the classifier from the entire training set using the best hyperparameters (with the highest AUC) identified through cross-validation and applied the best model to the test set. The highest AUC values were obtained using kernel = linear, gamma = 0.000001, and *C* = 0.01 (code available on GitHub at https://github.com/vealvarez/SVM_GC1).

The SCM (41) is a learning algorithm that produces models that are conjunctions (logical-AND) or disjunctions (logical-OR) of boolean-valued rules *r*: ℝ^*d*^ → {0,1}. Let us use *h*(*x*) to denote the output of model *h* on genome *x*. When *h* consists of a conjunction (i.e., a logical-AND) of a set R^*^ of rules r^*^, we have

$$h\left(x\right)=\underset{r^{\text{*}}\in R^{\text{*}}}{⋀}r^{\text{*}}(x)$$

whereas, for a disjunction (i.e., a logical-OR) of rules, we have:

$$h\left(x\right)=\underset{r^{\text{*}}\in R^{\text{*}}}{⋁}r^{\text{*}}(x)$$

given a set *R* of candidate rules, the SCM algorithm attempts to find a model that minimizes the empirical error rate

$$R_{S\;}\overset{\text{def}}{=}\;\frac{1}{m}\overset{m}{\underset{i=1}{\sum }}I\left[h(x_{i})\neq \;y_{i}\right]$$

The function *I* is defined as I[condition] = 1, if the condition is true; *I*[condition] = 0, if the condition is false, when using the smallest number of rules in *R* (29).

We used the program Kover, which implements the SCM algorithm (29, 104). Kover combines the SCM algorithm with the k-mer representation of genomes, which reveals uncharacteristically sparse models that explicitly highlight the relationship between genomic variations and the phenotype of interest (29). We ran the program using data set 1 (see “Input data set preparation for ML models” below) for k-mer sizes from 31 to 127 nucleotides (taking only the odd numbers between them). The smallest value of k was set to 31 since extensive testing has shown that this size is optimal for bacterial genome assembly and has been employed for studies based on reference-free bacterial genome comparisons (87, 107). The greatest k-mer size was set to 127 since it is the maximum value accepted as a parameter in Kover. We chose only odd values of k to avoid the formation of palindromes (108). For each k-mer size, we split data set 1 into a training data set (two-thirds of the genomes) and a testing data set (one-third of the genomes). Then, we trained the 49 models corresponding to each k-mer by using a conjunction/disjunction model type. The best conjunctive and/or disjunctive model for each k-mer was selected using 5-fold cross-validation to determine the optimal rule scoring function with default parameters.

### Input data set preparation for ML models.

We split the 500 genomes included in data set 1 into unitigs by using the program DBGWAS (62). Unitigs are stretches of DNA shared by the strains in a data set. The DBGWAS method proposes connecting the overlaps of k-mers in a compressed de Bruijn graph (DBG) so that k-mers are extended using the adjacent sequence information in the population, forming unitigs present in the same set of samples as their constituent k-mers. During the first step of the DBGWAS process, the program built a variant matrix, where each variant is a pattern of the presence/absence of unitigs in each genome present in data set 1 (62). We wrote a Python script (code available on GitHub at https://github.com/vealvarez/SVM_GC1) to format the variant matrix and create a presence/absence (coded with the values 1 and 0, respectively) binary matrix with unitigs as columns (features) and the accession numbers of the genomes as rows (instances). We used the binary matrix as input for the SVM algorithm. In this matrix, we discarded the data about low-frequency unitigs (unitigs found in fewer than 50 A. baumannii GC1 and non-GC1 genomes). We also integrated the MLST data corresponding to data set 1 genomes and created a two-column matrix used as input for the SVM algorithm. The first column of the matrix contained the accession number of the genomes, and the second column contained a binary variable that indicated whether each genome was typed or not typed as A. baumannii GC1 (−1 = A. baumannii non-GC1 genome and 1 = A. baumannii GC1 genome) according to MLST typing.

For the SCM approach, we first packaged data set 1 sequences stored in FASTA files into a Kover data set using the create from contigs command. This command also received a tab-separated value (TSV) with the data set 1 genome classification according to MLST typing (A. baumannii GC1 or non-GC1). The first column of the TSV file described the genome accession number, and the second column had the value 1 to indicate that the genome belonged to A. baumannii GC1 or the value 0 to indicate otherwise. We ran Kover for k-mer sizes from 31 to 127 nucleotides. For each k-mer size, Kover constructed a reference-free input matrix based on k-mer profiles generated with the DSK k-mer counting software. A k-mer presence/absence binary matrix based on data set 1 genomes was then created and used as input for the SCM models of each k-mer size.

### Unitig selection using SVM for putative biomarker analysis.

After obtaining the SVM model with the best hyperparameters, the values of the features weight vector (referred to as the hyperplane normal vector w* in the first equation in “Machine learning analyses” above) were accessed through the attribute sklearn.model_selection.GridSearchCV.best_estimator.coef_. The values were sorted from highest to lowest and used to decide the relevance of each unitig sequence (associated with each weight value) during the model prediction (109, 110). It is worth mentioning that in our study, sequence unitigs were used as features in the models. The positive sign of a feature weight value indicates that the feature contributes to A. baumannii GC1 class prediction (represented by the value 1) and the negative sign indicates that the feature contributes to A. baumannii non-GC1 class prediction (represented by the value −1) (111). Considering the above-mentioned, 100 unitig sequences with the highest weight values were selected to be analyzed as putative biomarkers of A. baumannii GC1 genomes.

### Machine learning performance metrics.

The performances of the SVM and SCM models were evaluated in terms of sensitivity, specificity, accuracy, precision, and F1 score. They were defined as follows: sensitivity = TP/(TP + FN), specificity = TN/(TN + FP), accuracy = (TP + TN)/(TP + FP + TN + FN), precision = TP/(TP + FP), and F1 score = 2 × ((precision × sensitivity)/(precision + sensitivity)), where TP (true positives) was the number of A. baumannii GC1 strains predicted to be A. baumannii GC1, TN (true negatives) was the number of A. baumannii non-GC1 strains predicted to be A. baumannii non-GC1, FP (false positives) was the number of A. baumannii non-GC1 strains predicted to be A. baumannii GC1, and FN (false negatives) was the number of A. baumannii GC1 strains predicted to be A. baumannii non-GC1.

### BLASTN searches.

BLASTN searches (112) were done using data set 1 and data set 2 as subjects, and the unitigs/k-mers that contributed most to A. baumannii GC1 genome prediction according to the SVM/SCM models were used as queries. We identified whether unitigs/k-mers matched known genes or intergenic regions and provide their putative function when possible. AYE (AN CU459141.1) and ACICU (AN CP000863.1) genomes were used as references to annotate the genome location and gene product related to A. baumannii GC1 and non-GC1 genomes, respectively. In cases where the unitig was not found in the AYE genome, the AB0057 (CP001182.2) genome was used instead. Also, we counted the number of A. baumannii GC1 and non-GC1 genomes matched by each unitig. We considered a cutoff E value of E^−10^, 100% of identity, and 100% of query cover. To analyze the target of the primers designed below, BLASTN searches (112) were done by using the primer sequence as the query and the nucleotide collection (nr/nt) database of GenBank or data set 1 and data set 2 as the subject. Finally, BLASTN searches (112) were also done by using the fragment of the U1 genomic biomarker of A. baumannii GC1 amplified with those primers (excluding the sequence of the primers) as the query and data set 1 and data set 2 as the subject.

### Primer design.

The primers to amplify the U1 genomic biomarker of A. baumannii GC1 were designed by using Oligo Primer Analysis software version 6.22 (113, 114).

## References

[1] Álvarez VE, Quiroga MP, Galán AV, Vilacoba E, Quiroga C, Ramírez MS, Centrón D. 2020. Crucial role of the accessory genome in the evolutionary trajectory of Acinetobacter baumannii global clone 1. Front Microbiol 11:342. doi:10.3389/fmicb.2020.00342. 32256462 PMC7093585

[2] Douraghi M, Kenyon JJ, Aris P, Asadian M, Ghourchian S, Hamidian M. 2020. Antibiotic resistance genes in carbapenem-resistant Acinetobacter baumannii isolates belonging to lineage 2, global clone 1, from outbreaks in 2012-2013 at a Tehran burns hospital. mSphere 5::e00164-20. doi:10.1128/mSphere.00164-20. 32269158 PMC7142300

[3] Hamidian M, Nigro SJ. 2019. Emergence, molecular mechanisms and global spread of carbapenem-resistant Acinetobacter baumannii. Microb Genom 5:e000306. doi:10.1099/mgen.0.000306. 31599224 PMC6861865

[4] Yakkala H, Samantarrai D, Gribskov M, Siddavattam D. 2019. Comparative genome analysis reveals niche-specific genome expansion in Acinetobacter baumannii strains. PLoS One 14:e0218204. doi:10.1371/journal.pone.0218204. 31194814 PMC6563999

[5] Holt K, Kenyon JJ, Hamidian M, Schultz MB, Pickard DJ, Dougan G, Hall R. 2016. Five decades of genome evolution in the globally distributed, extensively antibiotic-resistant Acinetobacter baumannii global clone 1. Microb Genom 2:e000052. doi:10.1099/mgen.0.000052. 28348844 PMC5320584

[6] Zarrilli R, Pournaras S, Giannouli M, Tsakris A. 2013. Global evolution of multidrug-resistant Acinetobacter baumannii clonal lineages. Int J Antimicrob Agents 41:11–19. doi:10.1016/j.ijantimicag.2012.09.008. 23127486

[7] Hamidian M, Wynn M, Holt KE, Pickard D, Dougan G, Hall RM. 2014. Identification of a marker for two lineages within the GC1 clone of Acinetobacter baumannii. J Antimicrob Chemother 69:557–558. doi:10.1093/jac/dkt379. 24080502 PMC3886933

[8] Provasi CJ, Cayô R, Girardello R, Gales AC. 2016. Diversity of mechanisms conferring resistance to β-lactams among OXA-23-producing Acinetobacter baumannii clones. Diagn Microbiol Infect Dis 85:90–97. doi:10.1016/j.diagmicrobio.2016.01.018. 26971181

[9] Rodríguez C, Nastro M, Flores S, Rodriguez M, Spinozzi M, Bruni G, López A, David V, Aiassa M, Marqués I, Navarro O, Paniccia L, Famiglietti A, Grupo Colaborativo Acinetobacter Argentina. 2018. Epidemiología molecular de aislados de Acinetobacter baumannii resistentes a carbapenems en Argentina. Rev Argent Microbiol 51:247–250. doi:10.1016/j.ram.2017.12.004. 30551810

[10] Hamed SM, Hussein AFA, Al-Agamy MH, Radwan HH, Zafer MM. 2022. Genetic configuration of genomic resistance islands in Acinetobacter baumannii clinical isolates from Egypt. Front Microbiol 13:878912. doi:10.3389/fmicb.2022.878912. 35935207 PMC9353178

[11] Koong J, Johnson C, Rafei R, Hamze M, Myers GSA, Kenyon JJ, Lopatkin AJ, Hamidian M. 2021. Phylogenomics of two ST1 antibiotic-susceptible non-clinical Acinetobacter baumannii strains reveals multiple lineages and complex evolutionary history in global clone 1. Microb Genom 7:000705. doi:10.1099/mgen.0.000705. 34874246 PMC8767349

[12] Dijkshoorn L, Nemec A, Seifert H. 2007. An increasing threat in hospitals: multidrug-resistant Acinetobacter baumannii. Nat Rev Microbiol 5:939–951. doi:10.1038/nrmicro1789. 18007677

[13] Bouvet PJM, Grimont PAD. 1986. Taxonomy of the genus Acinetobacter with the recognition of Acinetobacter baumannii sp. nov., Acinetobacter haemolyticus sp. nov., Acinetobacter johnsonii sp. nov., and Acinetobacter junii sp. nov. and emended descriptions of Acinetobacter calcoaceticus and Acinetobacter lwoffii. Int J Syst Bacteriol 36:228–240. doi:10.1099/00207713-36-2-228.

[14] Gerner-Smidt P, Tjernberg I, Ursing J. 1991. Reliability of phenotypic tests for identification of Acinetobacter species. J Clin Microbiol 29:277–282. doi:10.1128/jcm.29.2.277-282.1991. 2007635 PMC269753

[15] Bouvet PJM, Grimont PAD. 1987. Identification and biotyping of clinical isolates of Acinetobacter. Ann Inst Pasteur Microbiol 138:569–578. doi:10.1016/0769-2609(87)90042-1. 3440090

[16] Bartual SG, Seifert H, Hippler C, Luzon MAD, Wisplinghoff H, Rodríguez-Valera F. 2005. Development of a multilocus sequence typing scheme for characterization of clinical isolates of Acinetobacter baumannii. J Clin Microbiol 43:4382–4390. doi:10.1128/JCM.43.9.4382-4390.2005. 16145081 PMC1234098

[17] Gozalan A, Unaldı O, Guldemir D, Aydogan S, Kuzucu C, Cakirlar FK, Açıkgoz ZC, Durmaz R. 2020. Molecular characterization of carbapenem-resistant Acinetobacter baumannii blood culture isolates from three hospitals in Turkey. Jpn J Infect Dis 74:200–208. doi:10.7883/yoken.JJID.2020.478. 33250488

[18] Li L-H, Yang Y-S, Sun J-R, Huang T-W, Huang W-C, Chen F-J, Wang Y-C, Kuo T-H, Kuo S-C, Chen T-L, Lee Y-T, Chang Y-Y, Yang Y-S, Liu Y-M, Kuo S-C, Liu C-P, Chen T-L, Lee Y-T, Chang Y-Y, Yang Y-S, Liu Y-M, Kuo S-C, Liu C-P, Chen T-L, Lee Y-T. 2020. Clinical and molecular characterization of Acinetobacter seifertii in Taiwan. J Antimicrob Chemother 76:312–321. doi:10.1093/jac/dkaa432. 33128052

[19] Turton JF, Gabriel SN, Valderrey C, Kaufrnann ME, Pitt TL. 2007. Use of sequence-based typing and multiplex PCR to identify clonal lineages of outbreak strains of Acinetobacter baumannii. Clin Microbiol Infect 13:807–815. doi:10.1111/j.1469-0691.2007.01759.x. 17610600

[20] Evans BA, Hamouda A, Towner KJ, Amyes SGB. 2008. OXA-51-like β-lactamases and their association with particular epidemic lineages of Acinetobacter baumannii. Clin Microbiol Infect 14:268–275. doi:10.1111/j.1469-0691.2007.01919.x. 18190566

[21] Hamouda A, Evans BA, Towner KJ, Amyes SGB. 2010. Characterization of epidemiologically unrelated Acinetobacter baumannii isolates from four continents by use of multilocus sequence typing, pulsed-field gel electrophoresis, and sequence-based typing of blaOXA-51-like genes. J Clin Microbiol 48:2476–2483. doi:10.1128/JCM.02431-09. 20421437 PMC2897490

[22] Michalowitz A, Yang J, Castaneda P, Litrenta J. 2020. Existing and emerging methods of diagnosis and monitoring of pediatric musculoskeletal infection. Injury 51:2110–2117. doi:10.1016/j.injury.2020.06.020. 32732117

[23] Lupolova N, Lycett SJ, Gally DL. 2019. A guide to machine learning for bacterial host attribution using genome sequence data. Microb Genom 5:e000317. doi:10.1099/mgen.0.000317. 31778355 PMC6939162

[24] Holt KE, Wertheim H, Zadoks RN, Baker S, Whitehouse CA, Dance D, Jenney A, Connor TR, Hsu LY, Severin J, Brisse S, Cao H, Wilksch J, Gorrie C, Schultz MB, Edwards DJ, Van Nguyen K, Nguyen TV, Dao TT, Mensink M, Le Minh V, Nhu NTK, Schultsz C, Kuntaman K, Newton PN, Moore CE, Strugnell RA, Thomson NR. 2015. Genomic analysis of diversity, population structure, virulence, and antimicrobial resistance in Klebsiella pneumoniae, an urgent threat to public health. Proc Natl Acad Sci USA 112:E3574–E3581. doi:10.1073/pnas.1501049112. 26100894 PMC4500264

[25] Ezewudo M, Borens A, Chiner-Oms Á, Miotto P, Chindelevitch L, Starks AM, Hanna D, Liwski R, Zignol M, Gilpin C, Niemann S, Kohl TA, Warren RM, Crook D, Gagneux S, Hoffner S, Rodrigues C, Comas I, Engelthaler DM, Alland D, Rigouts L, Lange C, Dheda K, Hasan R, McNerney R, Cirillo DM, Schito M, Rodwell TC, Posey J. 2018. Integrating standardized whole genome sequence analysis with a global Mycobacterium tuberculosis antibiotic resistance knowledgebase. Sci Rep 8:15382. doi:10.1038/s41598-018-33731-1. 30337678 PMC6194142

[26] Cario CL, Chen E, Leong L, Emami NC, Lopez K, Tenggara I, Simko JP, Friedlander TW, Li PS, Paris PL, Carroll PR, Witte JS. 2020. A machine learning approach to optimizing cell-free DNA sequencing panels: with an application to prostate cancer. BMC Cancer 20:820. doi:10.1186/s12885-020-07318-x. 32859160 PMC7456018

[27] Ransom EM, Potter RF, Dantas G, Burnham CAD. 2020. Genomic prediction of antimicrobial resistance: ready or not, here it comes! Clin Chem 66:1278–1289. doi:10.1093/clinchem/hvaa172. 32918462

[28] Hernández M, Quijada NM, Rodríguez-Lázaro D, Eiros JM. 2020. Bioinformatics of next generation sequencing in clinical microbiology diagnosis. Rev Argent Microbiol 52:150–161. doi:10.1016/j.ram.2019.06.003. (In Spanish.) 31784184

[29] Drouin A, Giguère S, Déraspe M, Marchand M, Tyers M, Loo VG, Bourgault A-M, Laviolette F, Corbeil J. 2016. Predictive computational phenotyping and biomarker discovery using reference-free genome comparisons. BMC Genomics 17:754. doi:10.1186/s12864-016-2889-6. 27671088 PMC5037627

[30] Lees JA, Mai TT, Galardini M, Wheeler NE, Horsfield ST, Parkhill J, Corander J. 2020. Improved prediction of bacterial genotype-phenotype associations using interpretable pangenome-spanning regressions. mBio 11:e01344-20. doi:10.1128/mBio.01344-20. 32636251 PMC7343994

[31] Khaledi A, Weimann A, Schniederjans M, Asgari E, Kuo T, Oliver A, Cabot G, Kola A, Gastmeier P, Hogardt M, Jonas D, Mofrad MR, Bremges A, McHardy AC, Häussler S. 2020. Predicting antimicrobial resistance in Pseudomonas aeruginosa with machine learning-enabled molecular diagnostics. EMBO Mol Med 12:e10264. doi:10.15252/emmm.201910264. 32048461 PMC7059009

[32] Hyun JC, Kavvas ES, Monk JM, Palsson BO. 2020. Machine learning with random subspace ensembles identifies antimicrobial resistance determinants from pan-genomes of three pathogens. PLoS Comput Biol 16:e1007608. doi:10.1371/journal.pcbi.1007608. 32119670 PMC7067475

[33] Van Camp PJ, Haslam DB, Porollo A. 2020. Prediction of antimicrobial resistance in Gram-negative bacteria from whole-genome sequencing data. Front Microbiol 11:1013. doi:10.3389/fmicb.2020.01013. 32528441 PMC7262952

[34] Aytan-Aktug D, Clausen PTLC, Bortolaia V, Aarestrup FM, Lund O. 2020. Prediction of acquired antimicrobial resistance for multiple bacterial species using neural networks. mSystems 5:e00774-19. doi:10.1128/mSystems.00774-19. PMC6977075 31964771

[35] Lakin SM, Kuhnle A, Alipanahi B, Noyes NR, Dean C, Muggli M, Raymond R, Abdo Z, Prosperi M, Belk KE, Morley PS, Boucher C. 2019. Hierarchical hidden Markov models enable accurate and diverse detection of antimicrobial resistance sequences. Commun Biol 2:294. doi:10.1038/s42003-019-0545-9. 31396574 PMC6684577

[36] Saravanan R, Sujatha P. 2019. A state of art techniques on machine learning algorithms: a perspective of supervised learning approaches in data classification, p 945–949. *In* Proceedings of the 2nd International Conference on Intelligent Computing and Control Systems, ICICCS 2018. Institute of Electrical and Electronics Engineers, Inc.

[37] Macesic N, Polubriaginof F, Tatonetti NP. 2017. Machine learning: novel bioinformatics approaches for combating antimicrobial resistance. Curr Opin Infect Dis 30:511–517. doi:10.1097/QCO.0000000000000406. 28914640

[38] González L, Angulo C, Velasco F, Català A. 2005. Unified dual for bi-class SVM approaches. Pattern Recognit 38:1772–1774. doi:10.1016/j.patcog.2005.03.019.

[39] Scholkopf B, Smola A. 2001. Learning with kernels: support vector machines, regularization, optimization, and beyond. MIT Press, Cambridge, MA. doi:10.1109/tnn.2005.848998.

[40] Boser BE, Guyon IM, Vapnik VN. 1992. Training algorithm for optimal margin classifiers, p 144–152. *In* Proceedings of the Fifth Annual ACM Workshop on Computational Learning Theory. ACM, New York, NY.

[41] Marchand M, Shawe-Taylor J. 2002. The set covering machine. J Machine Learn Res 3:723–746.

[42] Ivanenkov YA, Zhavoronkov A, Yamidanov RS, Osterman IA, Sergiev PV, Aladinskiy VA, Aladinskaya AV, Terentiev VA, Veselov MS, Ayginin AA, Kartsev VG, Skvortsov DA, Chemeris AV, Baimiev AK, Sofronova AA, Malyshev AS, Filkov GI, Bezrukov DS, Zagribelnyy BA, Putin EO, Puchinina MM, Dontsova OA. 2019. Identification of novel antibacterials using machine learning techniques. Front Pharmacol 10:913. doi:10.3389/fphar.2019.00913. 31507413 PMC6719509

[43] Kouchaki S, Yang YY, Walker TM, Walker AS, Wilson DJ, Peto TEA, Crook DW, Clifton DA, Hoosdally SJ, Gibertoni CA, Carter J, Grazian C, Kouchaki S, Walker TM, Fowler PW, Clifton DA, Iqbal Z, Hunt M, Smith EG, Rathod P, Jarrett L, Matias D, Cirillo DM, Borroni E, Battaglia S, Ghodousi A, Spitaleri A, Cabibbe A, Tahseen S, Nilgiriwala K, Shah S, Rodrigues C, Kambli P, Surve U, Khot R, Niemann S, Kohl T, Merker M, Hoffmann H, Molodtsov N, Plesnik S, Ismail N, Omar SV, Joseph L, Marubini E, Thwaites G, Thuong TNT, Ngoc NH, Srinivasan V, Moore D, CRyPTIC Consortium, et al. 2019. Application of machine learning techniques to tuberculosis drug resistance analysis. Bioinformatics 35:2276–2282. doi:10.1093/bioinformatics/bty949. 30462147 PMC6596891

[44] Her HL, Wu YW. 2018. A pan-genome-based machine learning approach for predicting antimicrobial resistance activities of the Escherichia coli strains. Bioinformatics 34:i89–i95. doi:10.1093/bioinformatics/bty276. 29949970 PMC6022653

[45] Yang Y, Niehaus KE, Walker TM, Iqbal Z, Walker AS, Wilson DJ, Peto TEA, Crook DW, Smith EG, Zhu T, Clifton DA. 2018. Machine learning for classifying tuberculosis drug-resistance from DNA sequencing data. Bioinformatics 34:1666–1671. doi:10.1093/bioinformatics/btx801. 29240876 PMC5946815

[46] Zvezdanova ME, Arroyo MJ, Méndez G, Guinea J, Mancera L, Muñoz P, Rodríguez-Sánchez B, Escribano P. 2020. Implementation of MALDI-TOF mass spectrometry and peak analysis: application to the discrimination of cryptococcus neoformans species complex and their interspecies hybrids. J Fungi 6:330. doi:10.3390/jof6040330. PMC7711916 33276478

[47] Lorenz B, Ali N, Bocklitz T, Rösch P, Popp J. 2020. Discrimination between pathogenic and non-pathogenic E. coli strains by means of Raman microspectroscopy. Anal Bioanal Chem 412:8241–8247. doi:10.1007/s00216-020-02957-2. 33033893 PMC7680742

[48] Uysal Ciloglu F, Saridag AM, Kilic IH, Tokmakci M, Kahraman M, Aydin O. 2020. Identification of methicillin-resistant Staphylococcus aureus bacteria using surface-enhanced Raman spectroscopy and machine learning techniques. Analyst 145:7559–7570. doi:10.1039/d0an00476f. 33135033

[49] Guyon I, Weston J, Barnhill S, Labs T, Bank R. 2002. Gene selection for cancer classification using support vector machines. Machine Learning 46:389–422. doi:10.1023/A:1012487302797.

[50] Henneges C, Bullinger D, Fux R, Friese N, Seeger H, Neubauer H, Laufer S, Gleiter CH, Schwab M, Zell A, Kammerer B. 2009. Prediction of breast cancer by profiling of urinary RNA metabolites using support vector machine-based feature selection. BMC Cancer 9:104. doi:10.1186/1471-2407-9-104. 19344524 PMC2680413

[51] Aswathy MA, Jagannath M. 2021. An SVM approach towards breast cancer classification from H&E-stained histopathology images based on integrated features. Med Biol Eng Comput 59:1773–1783. doi:10.1007/s11517-021-02403-0. 34302269

[52] Xu H, Park S, Hwang TH. 2020. Computerized classification of prostate cancer Gleason scores from whole slide images. IEEE/ACM Trans Comput Biol Bioinform 17:1871–1882. doi:10.1109/TCBB.2019.2941195. 31536012

[53] Liu Z, Deng D, Lu H, Sun J, Lv L, Li S, Peng G, Ma X, Li J, Li Z, Rong T, Wang G. 2020. Evaluation of machine learning models for predicting antimicrobial resistance of Actinobacillus pleuropneumoniae from whole genome sequences. Front Microbiol 11:48. doi:10.3389/fmicb.2020.00048. 32117101 PMC7016212

[54] Li Y, Kong Y, Zhang M, Yan A, Liu Z. 2016. Using support vector machine (SVM) for classification of selectivity of H1N1 neuraminidase inhibitors. Mol Inform 35:116–124. doi:10.1002/minf.201500107. 27491921

[55] Hicks AL, Wheeler N, Sánchez-Busó L, Rakeman JL, Harris SR, Grad YH. 2019. Evaluation of parameters affecting performance and reliability of machine learning-based antibiotic susceptibility testing from whole genome sequencing data. PLoS Comput Biol 15:e1007349. doi:10.1371/journal.pcbi.1007349. 31479500 PMC6743791

[56] Bron EE, Smits M, Niessen WJ, Klein S. 2015. Feature selection based on the SVM weight vector for classification of dementia. IEEE J Biomed Health Inform 19:1617–1626. doi:10.1109/JBHI.2015.2432832. 25974958

[57] Kolisetty VV, Rajput DS. 2020. A review on the significance of machine learning for data analysis in big data. Jordanian J Comput Inf Technol 6:41–57.

[58] Diancourt L, Passet V, Nemec A, Dijkshoorn L, Brisse S. 2010. The population structure of Acinetobacter baumannii: expanding multiresistant clones from an ancestral susceptible genetic pool. PLoS One 5:e10034. doi:10.1371/journal.pone.0010034. 20383326 PMC2850921

[59] Karah N, Wai SN, Uhlin BE. 2021. CRISPR-based subtyping to track the evolutionary history of a global clone of Acinetobacter baumannii. Infect Genet Evol 90:104774. doi:10.1016/j.meegid.2021.104774. 33618003

[60] Hamidian M, Hall RM. 2018. The AbaR antibiotic resistance islands found in Acinetobacter baumannii global clone 1—structure, origin and evolution. Drug Resist Updat 41:26–39. doi:10.1016/j.drup.2018.10.003. 30472242

[61] Hamidian M, Hall RM. 2021. Dissemination of novel Tn7 family transposons carrying genes for synthesis and uptake of fimsbactin siderophores among Acinetobacter baumannii isolates. Microb Genom 7:mgen000548. doi:10.1099/mgen.0.000548. 33749577 PMC8190619

[62] Jaillard M, Lima L, Tournoud M, Mahé P, van Belkum A, Lacroix V, Jacob L. 2018. A fast and agnostic method for bacterial genome-wide association studies: bridging the gap between k-mers and genetic events. PLoS Genet 14:e1007758. doi:10.1371/journal.pgen.1007758. 30419019 PMC6258240

[63] Wuebbens MM, Liu MTW, Rajagopalan KV, Schindelin H. 2000. Insight into molybdenum cofactor deficiency provided by the crystal structure of the molybdenum cofactor biosynthesis protein MoaC. Structure 8:709–718. doi:10.1016/S0969-2126(00)00157-X. 10903949

[64] Winter SE, Thiennimitr P, Winter MG, Butler BP, Huseby DL, Crawford RW, Russell JM, Bevins CL, Adams LG, Tsolis RM, Roth JR, Bäumler AJ. 2010. Gut inflammation provides a respiratory electron acceptor for Salmonella. Nature 467:426–429. doi:10.1038/nature09415. 20864996 PMC2946174

[65] Winter SE, Winter MG, Xavier MN, Thiennimitr P, Poon V, Keestra AM, Laughlin RC, Gomez G, Wu J, Lawhon SD, Popova IE, Parikh SJ, Adams LG, Tsolis RM, Stewart VJ, Bäumler AJ. 2013. Host-derived nitrate boosts growth of E. coli in the inflamed gut. Science 339:708–711. doi:10.1126/science.1232467. 23393266 PMC4004111

[66] Denkel LA, Rhen M, Bange FC. 2013. Biotin sulfoxide reductase contributes to oxidative stress tolerance and virulence in Salmonella enterica serovar Typhimurium. Microbiology (Reading, Engl) 159:1447–1458. doi:10.1099/mic.0.067256-0. 23657680

[67] Williams M, Mizrahi V, Kana BD. 2014. Molybdenum cofactor: a key component of Mycobacterium tuberculosis pathogenesis? Crit Rev Microbiol 40:18–29. doi:10.3109/1040841X.2012.749211. 23317461

[68] Vilacoba E, Déraspe M, Traglia GM, Roy PH, Ramírez S. 2014. Draft genome sequence of an international clonal lineage 1 Acinetobacter baumannii strain from Argentina. Genome Announc 2:e01190-14. doi:10.1128/genomeA.01190-14. PMC4246157 25428965

[69] Arivett BA, Fiester SE, Ream DC, Centrón D, Ramírez MS, Tolmasky ME, Actis LA. 2015. Draft genome of the multidrug-resistant Acinetobacter baumannii strain A155 clinical isolate. Genome Announc 3:e00212-15. doi:10.1128/genomeA.00212-15. 25814610 PMC4384150

[70] Navon-Venezia S, Kondratyeva K, Carattoli A. 2017. Klebsiella pneumoniae: a major worldwide source and shuttle for antibiotic resistance. FEMS Microbiol Rev 41:252–275. doi:10.1093/femsre/fux013. 28521338

[71] Mathers A, Peirano G, Mathers AJ, Peirano G, Pitout DD. 2015. The role of epidemic resistance plasmids and international high-risk clones in the spread of multidrug-resistant Enterobacteriaceae. Clin Microbiol Rev 28:565–591. doi:10.1128/CMR.00116-14. 25926236 PMC4405625

[72] Abdouchakour F, Aujoulat F, Licznar-Fajardo P, Marchandin H, Toubiana M, Parer S, Lotthé A, Jumas-Bilak E. 2018. Intraclonal variations of resistance and phenotype in Pseudomonas aeruginosa epidemic high-risk clone ST308: a key to success within a hospital? Int J Med Microbiol 308:279–289. doi:10.1016/j.ijmm.2017.11.008. 29276044

[73] Villa L, Feudi C, Fortini D, Brisse S, Passet V, Bonura C, Endimiani A, Mammina C, Ocampo AM, Jimenez JN, Doumith M, Woodford N, Hopkins K, Carattoli A. 2017. Diversity, virulence, and antimicrobial resistance of the KPC-producing Klebsiella pneumoniae ST307 clone. Microb Genom 3:e000110. doi:10.1099/mgen.0.000110. 28785421 PMC5506382

[74] Oteo J, Pérez-Vázquez M, Bautista V, Ortega A, Zamarrón P, Saez D, Fernández-Romero S, Lara N, Ramiro R, Aracil B, Campos J, Spanish Antibiotic Resistance Surveillance Program Collaborating Group. 2016. The spread of KPC-producing Enterobacteriaceae in Spain: WGS analysis of the emerging high-risk clones of Klebsiella pneumoniae ST11/KPC-2, ST101/KPC-2 and ST512/KPC-3. J Antimicrob Chemother 71:3392–3399. doi:10.1093/jac/dkw321. 27530752 PMC5890657

[75] Cao H, Xia T, Li Y, Xu Z, Bougouffa S, Lo YK, Bajic VB, Luo H, Woo PCY, Yan A. 2019. Uncoupled quorum sensing modulates the interplay of virulence and resistance in a multidrug-resistant clinical Pseudomonas aeruginosa isolate belonging to the MLST550 clonal complex. Antimicrob Agents Chemother 63:e01944-18. doi:10.1128/AAC.01944-18. 30670423 PMC6437519

[76] Heiden SE, Hübner NO, Bohnert JA, Heidecke CD, Kramer A, Balau V, Gierer W, Schaefer S, Eckmanns T, Gatermann S, Eger E, Guenther S, Becker K, Schaufler K. 2020. A Klebsiella pneumoniae ST307 outbreak clone from Germany demonstrates features of extensive drug resistance, hypermucoviscosity, and enhanced iron acquisition. Genome Med 12:113. doi:10.1186/s13073-020-00814-6. 33298160 PMC7724794

[77] Gato E, Vázquez-Ucha JC, Rumbo-Feal S, Álvarez-Fraga L, Vallejo JA, Martínez-Guitián M, Beceiro A, Vivas JR, Sola Campoy PJ, Pérez-Vázquez M, Iglesias JO, Rodiño-Janeiro BK, Romero A, Poza M, Bou G, Pérez A. 2020. Kpi, a chaperone-usher pili system associated with the worldwide-disseminated high-risk clone Klebsiella pneumoniae ST-15. Proc Natl Acad Sci USA 117:17249–17259. doi:10.1073/pnas.1921393117. 32641516 PMC7382220

[78] de Lagarde M, Vanier G, Arsenault J, Fairbrother JM. 2021. High risk clone: a proposal of criteria adapted to the One Health context with application to enterotoxigenic Escherichia coli in the pig population. Antibiotics 10:244–219. doi:10.3390/antibiotics10030244. 33671102 PMC8000703

[79] Zhong Q, Kobe B, Kappler U. 2020. Molybdenum enzymes and how they support virulence in pathogenic bacteria. Front Microbiol 11:615860. doi:10.3389/fmicb.2020.615860. 33362753 PMC7759655

[80] Contreras I, Toro CS, Troncoso G, Mora GC. 1997. Salmonella typhi mutants defective in anaerobic respiration are impaired in their ability to replicate within epithelial cells. Microbiology 143:2665–2672. doi:10.1099/00221287-143-8-2665. 9274020

[81] Rosas-Magallanes V, Stadthagen-Gomez G, Rauzier J, Barreiro LB, Tailleux L, Boudou F, Griffin R, Nigou J, Jackson M, Gicquel B, Neyrolles O. 2007. Signature-tagged transposon mutagenesis identifies novel Mycobacterium tuberculosis genes involved in the parasitism of human macrophages. Infect Immun 75:504–507. doi:10.1128/IAI.00058-06. 17030567 PMC1828433

[82] Brodin P, Poquet Y, Levillain F, Peguillet I, Larrouy-Maumus G, Gilleron M, Ewann F, Christophe T, Fenistein D, Jang J, Jang M-S, Park S-J, Rauzier J, Carralot J-P, Shrimpton R, Genovesio A, Gonzalo-Asensio JA, Puzo G, Martin C, Brosch R, Stewart GR, Gicquel B, Neyrolles O. 2010. High content phenotypic cell-based visual screen identifies Mycobacterium tuberculosis acyltrehalose-containing glycolipids involved in phagosome remodeling. PLoS Pathog 6:e1001100. doi:10.1371/journal.ppat.1001100. 20844580 PMC2936551

[83] Dutta NK, Mehra S, Didier PJ, Roy CJ, Doyle LA, Alvarez X, Ratterree M, Be NA, Lamichhane G, Jain SK, Lacey MR, Lackner AA, Kaushal D. 2010. Genetic requirements for the survival of tubercle bacilli in primates. J Infect Dis 201:1743–1752. doi:10.1086/652497. 20394526 PMC2862080

[84] Levillain F, Poquet Y, Mallet L, Mazères S, Marceau M, Brosch R, Bange F-C, Supply P, Magalon A, Neyrolles O. 2017. Horizontal acquisition of a hypoxia-responsive molybdenum cofactor biosynthesis pathway contributed to Mycobacterium tuberculosis pathoadaptation. PLoS Pathog 13:e1006752. doi:10.1371/journal.ppat.1006752. 29176894 PMC5720804

[85] Macesic N, Bear Don’t Walk OJ, Pe’er I, Tatonetti NP, Peleg AY, Uhlemann A-C. 2020. Predicting phenotypic polymyxin resistance in Klebsiella pneumoniae through machine learning analysis of genomic data. mSystems 5:e00656-19. doi:10.1128/mSystems.00656-19. 32457240 PMC7253370

[86] Deelder W, Christakoudi S, Phelan J, Benavente ED, Campino S, McNerney R, Palla L, Clark TG. 2019. Machine learning predicts accurately mycobacterium tuberculosis drug resistance from whole genome sequencing data. Front Genet 10:922. doi:10.3389/fgene.2019.00922. 31616478 PMC6775242

[87] Mahé P, Tournoud M. 2018. Predicting bacterial resistance from whole-genome sequences using k-mers and stability selection. BMC Bioinformatics 19:383. doi:10.1186/s12859-018-2403-z. 30332990 PMC6192184

[88] Leski TA, Bangura U, Jimmy DH, Ansumana R, Lizewski SE, Li RW, Stenger DA, Taitt CR, Vora GJ. 2013. Identification of blaOXA-51-like, blaOXA-58, bla DIM-1, and blaVIM carbapenemase genes in hospital Enterobacteriaceae isolates from Sierra Leone. J Clin Microbiol 51:2435–2438. doi:10.1128/JCM.00832-13. 23658259 PMC3697688

[89] Lee YT, Kuo SC, Chiang MC, Yang SP, Chen CP, Chen TL, Fung CP. 2012. Emergence of carbapenem-resistant non-baumannii species of Acinetobacter harboring a blaOXA-51-like gene that is intrinsic to A. baumannii. Antimicrob Agents Chemother 56:1124–1127. doi:10.1128/AAC.00622-11. 22083478 PMC3264228

[90] Lee YT, Turton JF, Chen TL, Wu RCC, Chang WC, Fung CP, Chen CP, Cho WL, Huang LY, Siu LK. 2009. First identification of blaOXA-51-like in non-baumannii acinetobacter spp. J Chemother 21:514–520. doi:10.1179/joc.2009.21.5.514. 19933042

[91] Jaillard M, Tournoud M, Lima L, Lacroix V, Veyrieras JB, Jacob L. 2017. Representing genetic determinants in bacterial GWAS with compacted de Bruijn graphs. bioRxiv. doi:10.1101/113563.

[92] Lees JA, Galardini M, Bentley SD, Weiser JN, Corander J. 2018. pyseer: a comprehensive tool for microbial pangenome-wide association studies. Bioinformatics 34:4310–4312. doi:10.1093/bioinformatics/bty539. 30535304 PMC6289128

[93] Aun E, Brauer A, Kisand V, Tenson T, Remm M. 2018. A k-mer-based method for the identification of phenotype-associated genomic biomarkers and predicting phenotypes of sequenced bacteria. PLoS Comput Biol 14:e1006434. doi:10.1371/journal.pcbi.1006434. 30346947 PMC6211763

[94] Cortes C, Vapnik V. 1995. Support-vector networks. Mach Learn 20:273–297. doi:10.1007/BF00994018.

[95] Srivastava PK, Yaduvanshi A, Singh SK, Islam T, Gupta M. 2016. Support vector machines and generalized linear models for quantifying soil dehydrogenase activity in agro-forestry system of mid altitude central Himalaya. Environ Earth Sci 75:1–15. doi:10.1007/s12665-015-5074-3.

[96] Shawe-Taylor J, Cristianini N. 2004. Kernel methods for pattern analysis. Cambridge University Press, Cambridge, United Kingdom.

[97] Salazar DA, Iván VJ, Salazar JC. 2012. Comparison between SVM and logistic regression: which one is better to discriminate? Rev Colomb Estadística 35:223–237. (In Spanish.)

[98] Verplancke T, Van Looy S, Benoit D, Vansteelandt S, Depuydt P, De Turck F, Decruyenaere J. 2008. Support vector machine versus logistic regression modeling for prediction of hospital mortality in critically ill patients with haematological malignancies. BMC Med Inform Decis Mak 8:56. doi:10.1186/1472-6947-8-56. 19061509 PMC2612652

[99] Pochet NLMM, Suykens JAK. 2006. Support vector machines versus logistic regression: improving prospective performance in clinical decision-making. Ultrasound Obstet Gynecol 27:607–608. doi:10.1002/uog.2791. 16715467

[100] Ajiboye AR, Abdullah-Arshah R, Qin H, Isah-Kebbe H. 2015. Evaluating the effect of dataset size on predictive model using supervised learning technique. Int J Softw Eng Comput Sci 1:75–84.

[101] Zhang Y, Ling C. 2018. A strategy to apply machine learning to small datasets in materials science. npj Comput Mater 4:25. doi:10.1038/s41524-018-0081-z.

[102] Schmidt J, Marques MRG, Botti S, Marques MAL. 2019. Recent advances and applications of machine learning in solid-state materials science. npj Comput Mater 5:83. doi:10.1038/s41524-019-0221-0.

[103] Rizk G, Lavenier D, Chikhi R. 2013. DSK: k-mer counting with very low memory usage. Bioinformatics 29:652–653. doi:10.1093/bioinformatics/btt020. 23325618

[104] Drouin A, Letarte G, Raymond F, Marchand M, Corbeil J, Laviolette F. 2019. Interpretable genotype-to-phenotype classifiers with performance guarantees. Sci Rep 9:4071. doi:10.1038/s41598-019-40561-2. 30858411 PMC6411721

[105] Vapnik VN. 2000. The nature of statistical learning theory. Springer, New York, NY.

[106] Pedregosa F, Varoquaux G, Gramfort A, Michel V, Thirion B, Grisel O, Blondel M, Prettenhofer P, Weiss R, Dubourg V, Vanderplas J, Passos A, Cournapeau D, Brucher M, Perrot M, Duchesnay É. 2011. Scikit-learn: machine learning in Python. J Mach Learn Res 12:2825–2830.

[107] Boisvert S, Raymond F, Godzaridis É, Laviolette F, Corbeil J. 2012. Ray Meta: scalable de novo metagenome assembly and profiling. Genome Biol 13:R122. doi:10.1186/gb-2012-13-12-r122. 23259615 PMC4056372

[108] Miller JR, Koren S, Sutton G. 2010. Assembly algorithms for next-generation sequencing data. Genomics 95:315–327. doi:10.1016/j.ygeno.2010.03.001. 20211242 PMC2874646

[109] Guyon I, Weston J, Barnhill S, Vapnik V. 2002. Gene selection for cancer classification using support vector machines. Mach Learn 46:389–422. doi:10.1023/A:1012487302797.

[110] Chang Y, Lin C. 2008. Feature ranking using linear SVM, p 53–64. *In* Proceedings of the Workshop on the Causation and Prediction Challenge at WCCI 2008.

[111] Zisserman A. 2013. Lecture 2 : the SVM classifier. *In* C19 Machine Learning Lectures Hilary 2015. Oxford University, Oxford, United Kingdom.

[112] Altschul SF, Gish W, Miller W, Myers EW, Lipman DJ. 1990. Basic local alignment search tool. J Mol Biol 215:403–410. doi:10.1016/S0022-2836(05)80360-2. 2231712

[113] Rychlik W, Rhoads RE. 1989. A computer program for choosing optimal oligonudeotides for filter hybridization, sequencing and in vitro amplification of DNA. Nucleic Acids Res 17:8543–8551. doi:10.1093/nar/17.21.8543. 2587212 PMC335026

[114] Rychlik W, Spencer WJ, Rhoads RE. 1990. Optimization of the annealing temperature for DNA amplification in vitro. Nucleic Acids Res 18:6409–6412. doi:10.1093/nar/18.21.6409. 2243783 PMC332522

