# Supplementary material for: Identification of a Specific Biomarker of Acinetobacter baumannii Global Clone 1 by Machine Learning and PCR Related to Metabolic Fitness of ESKAPE Pathogens
Source: mSystems. 2023 May 15;8(3):e00734-22. doi: 10.1128/msystems.00734-22 (PMC10308912; doi:10.1128/msystems.00734-22)
Supplement: TABLE S1 [file msystems.00734-22-s0003.pdf]

Table S1

Accesion Number	Class	ST Pasteur
CP001182	GC1	1
JQSF00000000	GC1	1
NZ_CP023029	GC1	1
CP001172	GC1	1
CP012952	GC1	81
NZ_CP027528	GC1	1
JXSV00000000	GC1	1
NZ_LS483472	GC1	1
NZ_CP020595	GC1	1
NZ_CP021782	GC1	1
NZ_CP024418	GC1	1
CP008706	GC1	1
APQW00000000	GC1	1
CP030106	GC1	1
CP010781	GC1	1
CU459141	GC1	1
CP041035	GC1	1
APRD00000000	GC1	1
GCF_002143965	GC1	1
GCF_900042755	GC1	1
GCF_000441955	GC1	1
GCF_001511895	GC1	1
GCF_001511935	GC1	1
GCF_000453205	GC1	1
GCF_000304695	GC1	1
GCF_001512255	GC1	1
GCF_001028265	GC1	1
GCF_900019975	GC1	1
GCF_000369365	GC1	1
GCF_001512155	GC1	1
GCF_001512235	GC1	1
GCF_900029375	GC1	1
GCF_000770605	GC1	1
GCF_001373535	GC1	1
GCF_000304655	GC1	1
GCF_001511975	GC1	1
GCF_001512195	GC1	1
GCF_001612415	GC1	1
GCF_000301435	GC1	1
GCF_000302035	GC1	1
GCF_000297595	GC1	1
GCF_001549575	GC1	1
GCF_000794125	GC1	1
GCF_001512075	GC1	1

GCF_000586655	GC1	1
GCF_003949635	GC1	1
GCF_001512175	GC1	1
GCF_003020215	GC1	1
GCF_001612435	GC1	1
GCF_000453885	GC1	1
GCF_003020205	GC1	1
GCF_001612455	GC1	1
GCF_001657725	GC1	1
GCF_901669955	GC1	1
GCF_001511875	GC1	1
GCF_000453045	GC1	1
GCF_001874945	GC1	1
GCF_003948175	GC1	1
GCF_001373575	GC1	1
GCF_003020415	GC1	1
GCF_006492065	GC1	1
GCF_901484875	GC1	1
GCF_004347305	GC1	1
GCF_900043985	GC1	1
GCF_900406755	GC1	1
GCF_000453105	GC1	1
GCF_003711885	GC1	1
GCF_000623035	GC1	1
GCF_006491985	GC1	1
GCF_900240205	GC1	1
GCF_001512215	GC1	1
GCF_001511915	GC1	1
GCF_006494215	GC1	1
GCF_000586635	GC1	1
GCF_900406745	GC1	1
GCF_000969385	GC1	1
GCF_003020405	GC1	1
GCF_006492525	GC1	1
GCF_900029355	GC1	1
GCF_000453085	GC1	1
GCF_003948995	GC1	1
GCF_000309275	GC1	1
GCF_003325575	GC1	1
GCF_000286615	GC1	1
GCF_003948695	GC1	1
GCF_001511995	GC1	1
GCF_003020365	GC1	1
GCF_006494165	GC1	1
GCF_003185745	GC1	1
GCF_003627465	GC1	1

GCF_003325665	GC1	1
GCF_002838005	GC1	1
GCF_001907145	GC1	1
GCF_900043995	GC1	1
GCF_003020225	GC1	1
GCF_900406765	GC1	1
GCF_000453945	GC1	1
GCF_000305315	GC1	20
GCF_000163355	GC1	81
GCF_002119265	GC1	81
CP018664	non-GC1	437
NZ_JPXZ00000000	non-GC1	79
AEOW01000000	non-GC1	404
AMDR01000001	non-GC1	3
CP000863	non-GC1	2
GCF_000369385	non-GC1	52
GCF_003336135	non-GC1	255
GCF_000876445	non-GC1	250
GCF_000981405	non-GC1	2
GCF_002573905	non-GC1	938
GCF_000297955	non-GC1	2
GCF_000215005	non-GC1	32
GCF_002951015	non-GC1	2
GCF_002093815	non-GC1	2
GCF_000805305	non-GC1	2
GCF_001707985	non-GC1	2
GCF_000623015	non-GC1	10
GCF_001861995	non-GC1	2
GCF_001997285	non-GC1	1168
GCF_001721475	non-GC1	437
GCF_001617945	non-GC1	2
GCF_003052285	non-GC1	2
GCF_000186645	non-GC1	2
GCF_001909135	non-GC1	2
GCF_000413915	non-GC1	39
GCF_002950515	non-GC1	2
GCF_003131095	non-GC1	1156
GCF_001936675	non-GC1	738
GCF_002811175	non-GC1	52
GCF_000584435	non-GC1	2
GCF_001612395	non-GC1	2
GCF_000584455	non-GC1	49
GCF_000341985	non-GC1	52
GCF_002238075	non-GC1	2
GCF_000802885	non-GC1	2
GCF_002573815	non-GC1	821

GCF_003332235	non-GC1	2
GCF_000584395	non-GC1	2
GCF_001864815	non-GC1	2
GCF_002927775	non-GC1	10
GCF_001661665	non-GC1	2
GCF_001680475	non-GC1	2
GCF_001541875	non-GC1	187
GCF_001693095	non-GC1	388
GCF_000966525	non-GC1	2
GCF_003356505	non-GC1	2
GCF_001862985	non-GC1	2
GCF_000162295	non-GC1	52
GCF_002573795	non-GC1	23
GCF_002072795	non-GC1	2
GCF_003336085	non-GC1	2
GCF_003184015	non-GC1	164
GCF_001516805	non-GC1	54
GCF_000761465	non-GC1	2
GCF_003006995	non-GC1	2
GCF_000584475	non-GC1	2
GCF_000417785	non-GC1	215
GCF_002928135	non-GC1	156
GCF_001052775	non-GC1	2
GCF_000584495	non-GC1	2
GCF_003355995	non-GC1	2
GCF_000417805	non-GC1	215
GCF_002837985	non-GC1	2
GCF_000580135	non-GC1	2
GCF_002277715	non-GC1	2
GCF_001648035	non-GC1	1035
GCF_001863465	non-GC1	2
GCF_000584515	non-GC1	499
GCF_000584535	non-GC1	500
GCF_002838015	non-GC1	2
GCF_003231135	non-GC1	1157
GCF_001611995	non-GC1	25
GCF_003352735	non-GC1	2
GCF_000737145	non-GC1	52
GCF_000368545	non-GC1	34
GCF_003007375	non-GC1	2
GCF_001693175	non-GC1	821
GCF_002803655	non-GC1	2
GCF_000804985	non-GC1	2
GCF_000584415	non-GC1	510
GCF_000214965	non-GC1	25
GCF_000278605	non-GC1	2

GCF_003070905	non-GC1	490
GCF_000787335	non-GC1	2
GCF_000692095	non-GC1	2
GCF_001420515	non-GC1	2
GCF_001055965	non-GC1	1140
GCF_002760695	non-GC1	85
GCF_001951035	non-GC1	2
GCF_003240555	non-GC1	113
GCF_001949945	non-GC1	2
GCF_000367885	non-GC1	2
GCF_002634325	non-GC1	2
GCF_001672535	non-GC1	2
GCF_000248275	non-GC1	2
GCF_001432275	non-GC1	85
GCF_000584555	non-GC1	2
GCF_002837945	non-GC1	203
GCF_002760715	non-GC1	85
GCF_002837905	non-GC1	575
GCA_001584465	GC1	1
GCA_003949475	GC1	1
GCA_008990245	GC1	1
GCA_013403505	GC1	1
GCA_016468915	GC1	1
GCA_016469235	GC1	1
GCA_016469315	GC1	1
GCA_016469355	GC1	81
GCA_016469595	GC1	19
GCA_016469835	GC1	1
GCA_016470115	GC1	1
GCA_016472495	GC1	1
GCA_016472915	GC1	1
GCA_016474375	GC1	1
GCA_016474575	GC1	1
GCA_016476205	GC1	1
GCA_016476365	GC1	623
GCA_016476385	GC1	81
GCA_016476805	GC1	1
GCA_016485265	GC1	1
GCA_016485645	GC1	1
GCA_016486385	GC1	19
GCA_016486565	GC1	81
GCA_016487365	GC1	1
GCA_016496445	GC1	1
GCA_016498365	GC1	1
GCA_016498625	GC1	1
GCA_016498705	GC1	1

GCA_016510835	GC1	1
GCA_016511855	GC1	1
GCA_016511875	GC1	1
GCA_016511895	GC1	1
GCA_016512035	GC1	1
GCA_016512055	GC1	1
GCA_016512075	GC1	1
GCA_016512095	GC1	1
GCA_016512135	GC1	1
GCA_016512815	GC1	1
GCA_016512915	GC1	1
GCA_016512935	GC1	1
GCA_016512955	GC1	1
GCA_016512975	GC1	1
GCA_016513015	GC1	1
GCA_016513035	GC1	1
GCA_016513055	GC1	1
GCA_016513075	GC1	1
GCA_016513115	GC1	1
GCA_016513135	GC1	1
GCA_016513155	GC1	1
GCA_016513175	GC1	1
GCA_016513395	GC1	20
GCA_016513415	GC1	20
GCA_016513575	GC1	20
GCA_016513615	GC1	20
GCA_016513655	GC1	20
GCA_016513935	GC1	623
GCA_016514115	GC1	623
GCA_016514475	GC1	623
GCA_016514515	GC1	623
GCA_016514595	GC1	623
GCA_016514615	GC1	1
GCA_016514675	GC1	623
GCA_016514715	GC1	623
GCA_016514755	GC1	623
GCA_016514975	GC1	623
GCA_016515635	GC1	1
GCA_016516715	GC1	623
GCA_016516895	GC1	623
GCA_016516995	GC1	623
GCA_016517095	GC1	623
GCA_016517165	GC1	623
GCA_016517175	GC1	623
GCA_016517315	GC1	1
GCA_016517435	GC1	623

GCA_016517455	GC1	1
GCA_016517595	GC1	1
GCA_016517635	GC1	81
GCA_016517765	GC1	623
GCA_016517805	GC1	1
GCA_016517885	GC1	81
GCA_016517965	GC1	20
GCA_016517985	GC1	1
GCA_016518035	GC1	1
GCA_016518045	GC1	20
GCA_016518145	GC1	1
GCA_016518155	GC1	81
GCA_016518355	GC1	1
GCA_016518435	GC1	81
GCA_016519305	GC1	81
GCA_016519345	GC1	81
GCA_016519375	GC1	81
GCA_016519385	GC1	81
GCA_016519795	GC1	1
GCA_016519975	GC1	19
GCA_016520245	GC1	19
GCA_016521015	GC1	1
GCA_016521295	GC1	19
GCA_016521325	GC1	19
GCA_016521645	GC1	19
GCA_016521905	GC1	19
GCF_000018445	non-GC1	2
GCF_000173395	non-GC1	49
GCF_000184475	non-GC1	2
GCF_000184495	non-GC1	16
GCF_000186665	non-GC1	267
GCF_000187205	non-GC1	2
GCF_000188215	non-GC1	2
GCF_000189655	non-GC1	2
GCF_000189675	non-GC1	2
GCF_000189695	non-GC1	78
GCF_000214985	non-GC1	3
GCF_000222245	non-GC1	2
GCF_000222265	non-GC1	415
GCF_000222285	non-GC1	2
GCF_000226275	non-GC1	2
GCF_000241705	non-GC1	25
GCF_000241725	non-GC1	2
GCF_000248195	non-GC1	69
GCF_000278625	non-GC1	3
GCF_000278645	non-GC1	3

GCF_000278665	non-GC1	25
GCF_000278685	non-GC1	2
GCF_000286535	non-GC1	3
GCF_000292545	non-GC1	218
GCF_000297515	non-GC1	414
GCF_000297535	non-GC1	136
GCF_000297575	non-GC1	513
GCF_000299655	non-GC1	2
GCF_000299675	non-GC1	2
GCF_000301175	non-GC1	2
GCF_000301195	non-GC1	417
GCF_000301215	non-GC1	2
GCF_000301235	non-GC1	113
GCF_000301255	non-GC1	113
GCF_000301275	non-GC1	2
GCF_000301295	non-GC1	241
GCF_000301315	non-GC1	415
GCF_000301335	non-GC1	25
GCF_000301355	non-GC1	416
GCF_000301375	non-GC1	2
GCF_000301395	non-GC1	2
GCF_000301415	non-GC1	417
GCF_000301455	non-GC1	2
GCF_000301475	non-GC1	2
GCF_000301495	non-GC1	2
GCF_000301515	non-GC1	406
GCF_000301535	non-GC1	406
GCF_000301555	non-GC1	406
GCF_000301575	non-GC1	2
GCF_000301595	non-GC1	2
GCF_000301615	non-GC1	2
GCF_000301655	non-GC1	2
GCF_000301835	non-GC1	413
GCF_000301855	non-GC1	422
GCF_000301875	non-GC1	429
GCF_000301895	non-GC1	241
GCF_000301915	non-GC1	2
GCF_000301935	non-GC1	416
GCF_000301955	non-GC1	415
GCF_000301975	non-GC1	417
GCF_000301995	non-GC1	417
GCF_000302015	non-GC1	2
GCF_000302055	non-GC1	2
GCF_000302075	non-GC1	2
GCF_000302095	non-GC1	2
GCF_000302135	non-GC1	438

GCF_000302155	non-GC1	417
GCF_000302175	non-GC1	406
GCF_000302195	non-GC1	2
GCF_000302215	non-GC1	2
GCF_000302235	non-GC1	241
GCF_000302255	non-GC1	422
GCF_000302575	non-GC1	2
GCF_000304675	non-GC1	405
GCF_000305235	non-GC1	10
GCF_000305255	non-GC1	2
GCF_000305275	non-GC1	515
GCF_000305295	non-GC1	3
GCF_000307895	non-GC1	412
GCF_000308995	non-GC1	2
GCF_000309095	non-GC1	32
GCF_000309115	non-GC1	136
GCF_000309135	non-GC1	32
GCF_000309155	non-GC1	49
GCF_000309175	non-GC1	2
GCF_000309215	non-GC1	3
GCF_000309235	non-GC1	403
GCF_000309255	non-GC1	428
GCF_000314635	non-GC1	412
GCF_000314655	non-GC1	2
GCF_000332855	non-GC1	2
GCF_000333715	non-GC1	2
GCF_000335535	non-GC1	155
GCF_000335595	non-GC1	158
GCF_000335615	non-GC1	431
GCF_000335635	non-GC1	665
GCF_000335655	non-GC1	2
GCF_000335675	non-GC1	2
GCF_000338835	non-GC1	255
GCF_000342065	non-GC1	2
GCF_000342085	non-GC1	516
GCF_000353795	non-GC1	2
GCF_000353815	non-GC1	2
GCF_000353855	non-GC1	2
GCF_000353895	non-GC1	2
GCF_000353915	non-GC1	2
GCF_000354035	non-GC1	2
GCF_000354055	non-GC1	2
GCF_000354075	non-GC1	2
GCF_000354095	non-GC1	2
GCF_000354115	non-GC1	2
GCF_000354135	non-GC1	2

GCF_000354155	non-GC1	2
GCF_000359725	non-GC1	2
GCF_000368105	non-GC1	3
GCF_000369165	non-GC1	38
GCF_000419385	non-GC1	2
GCF_000419405	non-GC1	23
GCF_000419425	non-GC1	2
GCF_000453025	non-GC1	78
GCF_000453065	non-GC1	2
GCF_000453145	non-GC1	2
GCF_000453165	non-GC1	425
GCF_000453185	non-GC1	2
GCF_000453225	non-GC1	406
GCF_000453245	non-GC1	406
GCF_000453265	non-GC1	406
GCF_000453285	non-GC1	2
GCF_000453305	non-GC1	2
GCF_000453325	non-GC1	2
GCF_000453345	non-GC1	2
GCF_000453365	non-GC1	2
GCF_000453385	non-GC1	2
GCF_000453405	non-GC1	2
GCF_000453425	non-GC1	2
GCF_000453445	non-GC1	6
GCF_000453465	non-GC1	427
GCF_000453485	non-GC1	417
GCF_000453505	non-GC1	417
GCF_000453525	non-GC1	2
GCF_000453545	non-GC1	2
GCF_000453565	non-GC1	2
GCF_000453585	non-GC1	2
GCF_000453605	non-GC1	33
GCF_000453625	non-GC1	79
GCF_000453645	non-GC1	2
GCF_000453665	non-GC1	2
GCF_000453685	non-GC1	406
GCF_000453705	non-GC1	2
GCF_000453725	non-GC1	411
GCF_000453745	non-GC1	2
GCF_000453765	non-GC1	2
GCF_000453785	non-GC1	2
GCF_000453805	non-GC1	2
GCF_000453825	non-GC1	2
GCF_000453845	non-GC1	2
GCF_000453865	non-GC1	2
GCF_000453905	non-GC1	2

GCF_000453925	non-GC1	2
GCF_000465635	non-GC1	221
GCF_000493615	non-GC1	25
GCF_000498375	non-GC1	2
GCF_000505685	non-GC1	639
GCF_000513795	non-GC1	2
GCF_000515435	non-GC1	2
GCF_000515455	non-GC1	2
GCF_000515475	non-GC1	2
GCF_000515495	non-GC1	2
GCF_000515515	non-GC1	2
GCF_000515535	non-GC1	2
GCF_000515555	non-GC1	2
GCF_000515575	non-GC1	79
GCF_000515595	non-GC1	2
GCF_000515615	non-GC1	2
GCF_000515635	non-GC1	2
GCF_000515655	non-GC1	2
GCF_000515675	non-GC1	2
GCF_000515695	non-GC1	2
GCF_000515715	non-GC1	2
GCF_000515735	non-GC1	2
GCF_000515755	non-GC1	79
GCF_000515775	non-GC1	2
GCF_000515795	non-GC1	79
GCF_000515815	non-GC1	2
GCF_000515835	non-GC1	2
GCF_000515855	non-GC1	79
GCF_000515875	non-GC1	524
GCF_000515895	non-GC1	2
GCF_000515915	non-GC1	2
GCF_000515935	non-GC1	2
GCF_000515955	non-GC1	2
GCF_000515975	non-GC1	2
GCF_000515995	non-GC1	2
GCF_000516015	non-GC1	2
GCF_000516035	non-GC1	2
GCF_000516055	non-GC1	2
GCF_000516075	non-GC1	2
GCF_000516095	non-GC1	2
GCF_000516115	non-GC1	2
GCF_000516135	non-GC1	406
