# Supplementary material for: Identification of a Specific Biomarker of Acinetobacter baumannii Global Clone 1 by Machine Learning and PCR Related to Metabolic Fitness of ESKAPE Pathogens
Source: mSystems. 2023 May 15;8(3):e00734-22. doi: 10.1128/msystems.00734-22 (PMC10308912; doi:10.1128/msystems.00734-22)
Supplement: TABLE S2 [file msystems.00734-22-s0004.pdf]

Table S2.

Accession Number	Class	ST Pasteur
CP027246	GC1	20
CP050403	GC1	1
CP058625	GC1	1
GCA_000353995	GC1	1
GCF_000021245	GC1	1
GCF_000069245	GC1	1
GCF_000163375	GC1	81
GCF_000302275	GC1	1
GCF_000305215	GC1	19
GCF_000309195	GC1	19
GCF_000353775	GC1	1
GCF_000353955	GC1	1
GCF_000354015	GC1	1
GCF_000369185	GC1	1
GCF_000454005	GC1	1
GCF_000830055	GC1	1
GCF_000836625	GC1	1
GCF_000963815	GC1	1
GCF_001028315	GC1	1
GCF_001028325	GC1	81
GCF_001612115	GC1	717
GCF_000177695	GC1	1
GCF_000177715	GC1	20
GCF_000177735	GC1	1
GCF_000369325	GC1	1
GCF_000731975	GC1	94
GCF_000750855	GC1	94
GCF_001028305	GC1	94
GCF_001028335	GC1	94
GCF_001028385	GC1	94
GCF_001399655	GC1	81
GCF_001443265	GC1	94
GCF_001444215	GC1	94
GCF_001444225	GC1	94
GCF_001444255	GC1	94
GCF_001444265	GC1	94
GCF_001511855	GC1	1
GCF_001512015	GC1	1
GCF_001512035	GC1	1
GCF_001512055	GC1	1
GCF_001512115	GC1	1
GCF_001512135	GC1	1
GCF_001612005	GC1	20

GCF_001612255	GC1	1
GCF_001612275	GC1	1
GCF_001704695	GC1	1
GCF_001907155	GC1	1
GCF_002082825	GC1	1
GCF_002119355	GC1	81
GCF_002137655	GC1	81
GCF_002150485	GC1	1
GCF_002210065	GC1	1
GCF_002245595	GC1	1
GCF_002245665	GC1	1
GCF_002288625	GC1	1
GCF_002416345	GC1	20
GCF_002416375	GC1	20
GCF_002416475	GC1	20
GCF_002741415	GC1	1
GCF_002803025	GC1	1
GCF_002837955	GC1	1
GCF_002838025	GC1	717
GCF_002927895	GC1	1
GCF_002996805	GC1	1
GCF_002999195	GC1	20
GCF_003296225	GC1	1
GCF_003345845	GC1	1
GCF_003345965	GC1	1
GCF_003522845	GC1	1
GCF_003615305	GC1	1
GCF_003670075	GC1	1
GCF_003940075	GC1	1
GCF_004357085	GC1	1
GCF_004357105	GC1	1
GCF_004357125	GC1	1
GCF_005819215	GC1	717
GCF_006385075	GC1	1
GCF_006491855	GC1	20
GCF_006491965	GC1	1
GCF_006492055	GC1	19
GCF_006492195	GC1	1
GCF_006492575	GC1	81
GCF_006492735	GC1	94
GCF_006494155	GC1	1
GCF_008806495	GC1	1
GCF_009268055	GC1	328
GCF_009445405	GC1	19
GCF_009497435	GC1	328
GCF_009497495	GC1	328

GCF_009497505	GC1	328
GCF_009497515	GC1	328
GCF_009497535	GC1	328
GCF_009497575	GC1	328
GCF_009497595	GC1	328
GCF_009497615	GC1	328
GCF_009754965	GC1	1
GCF_009759915	GC1	1
GCF_009760475	GC1	1
GCF_009760485	GC1	1
GCF_009829545	GC1	1
GCF_009887785	GC1	19
GCF_009887905	GC1	1
GCF_009901675	GC1	19
GCF_009901705	GC1	19
GCF_010500435	GC1	1
GCF_011603485	GC1	1
GCF_011603925	GC1	1
GCF_012276735	GC1	1
GCF_012935005	GC1	1
GCF_013138415	GC1	1
GCF_013138435	GC1	1
GCF_013138475	GC1	1
GCF_013185135	GC1	1
GCF_014334855	GC1	1
GCF_014334865	GC1	1
GCF_014334965	GC1	1
GCF_014530695	GC1	1
GCF_014531085	GC1	1
GCF_014531115	GC1	1
GCF_014531135	GC1	1
GCF_014531335	GC1	19
GCF_014531535	GC1	1
GCF_014531545	GC1	1
GCF_014531695	GC1	1
GCF_014531705	GC1	1
GCF_014531735	GC1	1
GCF_014531745	GC1	1
GCF_014622595	GC1	1
GCF_015265745	GC1	1
GCF_015536975	GC1	1
GCF_015537225	GC1	1
GCF_015537285	GC1	1
GCF_015537445	GC1	1
GCF_015537505	GC1	1
GCF_015537575	GC1	1

GCF_015537675	GC1	1
GCF_015537755	GC1	1
GCF_015537865	GC1	1
GCF_015537905	GC1	1
GCF_015537915	GC1	1
GCF_015537945	GC1	1
GCF_015537965	GC1	1
GCF_015537975	GC1	1
GCF_015538055	GC1	1
GCF_015538125	GC1	20
GCF_015538235	GC1	1
GCF_015538275	GC1	1
GCF_015538335	GC1	1
GCF_015538355	GC1	1
GCF_015666515	GC1	1
GCF_016055715	GC1	1
GCF_016055945	GC1	1
GCF_016056005	GC1	1
GCF_016056125	GC1	1
GCF_016127515	GC1	1
GCF_016416045	GC1	1
GCF_016654305	GC1	1
GCF_016762855	GC1	1
GCF_016767955	GC1	1
GCF_016767975	GC1	1
GCF_017168635	GC1	1
GCF_017168645	GC1	1
GCF_017168655	GC1	1
GCF_017168855	GC1	1
GCF_017168935	GC1	1
GCF_017168955	GC1	1
GCF_017168975	GC1	1
GCF_017169335	GC1	1
GCF_017169355	GC1	1
GCF_017169375	GC1	1
GCF_017909475	GC1	1
GCF_017960395	GC1	1
GCF_018156665	GC1	1
GCF_018156705	GC1	1
GCF_018156725	GC1	1
GCF_018361305	GC1	1
GCF_018445185	GC1	81
GCF_018445625	GC1	81
GCF_018788655	GC1	1
GCF_018788735	GC1	1
GCF_018788755	GC1	1

GCF_018831465	GC1	1
GCF_018831485	GC1	1
GCF_900019965	GC1	1
GCF_900020545	GC1	1
GCF_900029365	GC1	1
GCF_900031715	GC1	1
GCF_900067075	GC1	1
GCF_900406715	GC1	1
GCF_900476685	GC1	1
GCF_900476715	GC1	1
GCF_900476755	GC1	1
GCF_900476775	GC1	1
GCF_900476785	GC1	1
GCF_900476875	GC1	1
GCF_900476885	GC1	1
GCF_900476965	GC1	1
GCF_900477435	GC1	1
GCF_900477445	GC1	1
GCF_900477455	GC1	1
GCF_900477475	GC1	1
GCF_900477525	GC1	1
GCF_900478055	GC1	1
GCF_900478075	GC1	1
GCF_900478145	GC1	1
GCF_900490095	GC1	1
GCF_902702975	GC1	1
GCF_902703065	GC1	1
GCF_902703085	GC1	1
GCF_902703115	GC1	1
GCF_904884555	GC1	1
GCF_904885795	GC1	19
GCF_904885835	GC1	1
GCF_904886895	GC1	1
GCF_904886905	GC1	1
GCF_904887015	GC1	81
GCF_904890255	GC1	1
GCA_016522555	GC1	19
GCA_016522795	GC1	19
GCA_016523025	GC1	19
GCA_016523175	GC1	81
GCA_016523315	GC1	1
GCA_016523335	GC1	1
GCA_016523475	GC1	1
GCA_016523595	GC1	19
GCA_016523965	GC1	1
GCA_016524255	GC1	19

GCA_016524275	GC1	19
GCA_016524775	GC1	1
GCA_016524845	GC1	1
GCA_016524975	GC1	19
GCA_016525135	GC1	1
GCA_016527935	GC1	1
GCA_016528005	GC1	20
GCA_016528105	GC1	19
GCA_016528125	GC1	19
GCA_016528215	GC1	19
GCA_016528665	GC1	1
GCA_016530025	GC1	19
GCA_016530115	GC1	1
GCA_016532705	GC1	19
GCA_016532955	GC1	81
GCA_016533055	GC1	19
GCA_016534745	GC1	19
GCA_016535275	GC1	1
GCA_016535365	GC1	1
GCA_016535445	GC1	1
GCA_016535545	GC1	1
GCA_016535595	GC1	1
GCA_016535605	GC1	1
GCA_016535665	GC1	1
GCA_016535685	GC1	1
GCA_016535705	GC1	1
GCA_016535765	GC1	94
GCA_016535775	GC1	20
GCA_016535805	GC1	1
GCA_016535925	GC1	81
GCA_016536395	GC1	19
GCA_016536465	GC1	19
GCA_016537005	GC1	1
GCA_016537145	GC1	1
GCA_016537205	GC1	1
GCA_016537555	GC1	1
GCA_016537745	GC1	1
GCA_016538265	GC1	81
GCA_016538365	GC1	1
GCA_016538465	GC1	1
GCA_016538485	GC1	1
GCA_016538535	GC1	1
GCA_016538985	GC1	19
GCA_016539055	GC1	19
GCA_016539065	GC1	94
GCA_016539385	GC1	1

GCA_016540055	GC1	94
GCA_016540065	GC1	20
GCA_016540125	GC1	1
GCA_016540145	GC1	1
GCA_016540155	GC1	1
GCA_016540205	GC1	1
GCA_016540215	GC1	1
GCA_016540245	GC1	1
GCA_016540255	GC1	94
GCA_016540295	GC1	20
GCA_016540365	GC1	1
GCA_016540395	GC1	20
GCA_016540505	GC1	1
GCA_016540525	GC1	81
GCA_016540685	GC1	1
GCA_016540705	GC1	1
GCA_016540825	GC1	81
GCA_016540865	GC1	81
GCA_016540885	GC1	81
GCA_016540905	GC1	94
GCA_016540985	GC1	81
GCA_016541005	GC1	81
GCA_016541185	GC1	81
GCA_016541205	GC1	81
GCA_016609685	GC1	1
GCA_016609805	GC1	1
GCA_016610505	GC1	1
GCA_016973895	GC1	1
GCA_017953535	GC1	1
GCA_019188985	GC1	19
GCA_900490095	GC1	1
GCA_900607735	GC1	1
GCA_901484965	GC1	1
GCF_003346005	GC1	1106
GCF_004357185	GC1	1106
GCF_005819735	GC1	315
GCF_018422645	GC1	315
GCF_900476815	GC1	460
GCF_902713735	GC1	315
GCF_000163395	non-GC1	2
GCF_000297935	non-GC1	419
GCF_000368125	non-GC1	47
GCF_000368185	non-GC1	25
GCF_000368205	non-GC1	12
GCF_000368225	non-GC1	2
GCF_000368245	non-GC1	15

GCF_000368525	non-GC1	9
GCF_000369205	non-GC1	10
GCF_000369225	non-GC1	11
GCF_000369265	non-GC1	35
GCF_000369285	non-GC1	2
GCF_000369305	non-GC1	36
GCF_000369345	non-GC1	37
GCF_000804725	non-GC1	2
GCF_000804895	non-GC1	2
GCF_000804905	non-GC1	2
GCF_000804955	non-GC1	23
GCF_000804975	non-GC1	2
GCF_000805045	non-GC1	23
GCF_000805055	non-GC1	2
GCF_000805115	non-GC1	2
GCF_000805125	non-GC1	2
GCF_000805195	non-GC1	2
GCF_000805205	non-GC1	2
GCF_000805225	non-GC1	2
GCF_000805275	non-GC1	2
GCF_000805295	non-GC1	2
GCF_001541455	non-GC1	745
GCF_001541525	non-GC1	2
GCF_001541695	non-GC1	2
GCF_001541775	non-GC1	745
GCF_000297915	non-GC1	2
GCF_000369245	non-GC1	40
GCF_000802915	non-GC1	10
GCF_000804635	non-GC1	2
GCF_000804645	non-GC1	2
GCF_000804655	non-GC1	2
GCF_000804665	non-GC1	2
GCF_000804715	non-GC1	2
GCF_000804735	non-GC1	2
GCF_000804745	non-GC1	2
GCF_000804795	non-GC1	2
GCF_000804805	non-GC1	2
GCF_000804815	non-GC1	2
GCF_000804825	non-GC1	2
GCF_000804875	non-GC1	2
GCF_000804885	non-GC1	2
GCF_000804965	non-GC1	23
GCF_000805065	non-GC1	1554
GCF_000805135	non-GC1	105
GCF_000805145	non-GC1	-
GCF_000805215	non-GC1	10

GCF_001541685	non-GC1	-
GCF_001541705	non-GC1	187
GCF_001541725	non-GC1	187
GCF_001541765	non-GC1	187
GCF_001541795	non-GC1	187
GCF_001612015	non-GC1	3
GCF_001612025	non-GC1	48
GCF_001612075	non-GC1	2
GCF_001612095	non-GC1	25
GCF_001612105	non-GC1	25
GCF_001612155	non-GC1	2
GCF_001612175	non-GC1	2
GCF_001612185	non-GC1	2
GCF_001612215	non-GC1	3
GCF_001612235	non-GC1	2
GCF_001612285	non-GC1	2
GCF_001612315	non-GC1	2
GCF_001612335	non-GC1	2
GCF_001612355	non-GC1	2
GCF_001612365	non-GC1	2
GCF_001617835	non-GC1	2
GCF_001617885	non-GC1	2
GCF_001617915	non-GC1	2
GCF_001680115	non-GC1	2
GCF_001680185	non-GC1	2
GCF_001680195	non-GC1	2
GCF_001680215	non-GC1	2
GCF_001680225	non-GC1	2
GCF_001680265	non-GC1	2
GCF_001680285	non-GC1	2
GCF_001680295	non-GC1	2
GCF_001680305	non-GC1	2
GCF_001680345	non-GC1	2
GCF_001680365	non-GC1	2
GCF_001680375	non-GC1	2
GCF_001680395	non-GC1	2
GCF_001680425	non-GC1	2
GCF_001680445	non-GC1	2
GCF_001693115	non-GC1	23
GCF_001700445	non-GC1	79
GCF_001704675	non-GC1	10
GCF_001704685	non-GC1	2
GCF_001704705	non-GC1	-
GCF_001721375	non-GC1	-
GCF_001721385	non-GC1	437
GCF_001721425	non-GC1	437

GCF_001721445	non-GC1	437
GCF_001721455	non-GC1	437
GCF_001721465	non-GC1	437
GCF_001861395	non-GC1	-
GCF_001861435	non-GC1	2
GCF_001861465	non-GC1	2
GCF_001861515	non-GC1	2
GCF_001861535	non-GC1	2
GCF_001861545	non-GC1	2
GCF_001861585	non-GC1	2
GCF_001861615	non-GC1	2
GCF_001861655	non-GC1	2
GCF_001861665	non-GC1	2
GCF_000516155	non-GC1	78
GCF_000516175	non-GC1	2
GCF_000516195	non-GC1	2
GCF_000516215	non-GC1	2
GCF_000516235	non-GC1	2
GCF_000516255	non-GC1	669
GCF_000516275	non-GC1	79
GCF_000516295	non-GC1	2
GCF_000516315	non-GC1	79
GCF_000516335	non-GC1	2
GCF_000516355	non-GC1	2
GCF_000516375	non-GC1	79
GCF_000516575	non-GC1	25
GCF_000516595	non-GC1	2
GCF_000516635	non-GC1	25
GCF_000517485	non-GC1	2
GCF_000517505	non-GC1	2
GCF_000580155	non-GC1	514
GCF_000580175	non-GC1	523
GCF_000580195	non-GC1	2
GCF_000580235	non-GC1	2
GCF_000580255	non-GC1	416
GCF_000580275	non-GC1	2
GCF_000580295	non-GC1	2
GCF_000580315	non-GC1	537
GCF_000580335	non-GC1	2
GCF_000580355	non-GC1	500
GCF_000580375	non-GC1	2
GCF_000580395	non-GC1	2
GCF_000580415	non-GC1	16
GCF_000580435	non-GC1	416
GCF_000580455	non-GC1	527
GCF_000580475	non-GC1	2

GCF_000580495	non-GC1	499
GCF_000580515	non-GC1	241
GCF_000580535	non-GC1	416
GCF_000580555	non-GC1	2
GCF_000580575	non-GC1	2
GCF_000580595	non-GC1	529
GCF_000580615	non-GC1	203
GCF_000580695	non-GC1	512
GCF_000580715	non-GC1	503
GCF_000580735	non-GC1	2
GCF_000580755	non-GC1	499
GCF_000580775	non-GC1	528
GCF_000580815	non-GC1	536
GCF_000580835	non-GC1	2
GCF_000580875	non-GC1	240
GCF_000580975	non-GC1	508
GCF_000580995	non-GC1	10
GCF_000581015	non-GC1	2
GCF_000581055	non-GC1	25
GCF_000581075	non-GC1	103
GCF_000581135	non-GC1	25
GCF_000581155	non-GC1	49
GCF_000581175	non-GC1	2
GCF_000581235	non-GC1	2
GCF_000581255	non-GC1	509
GCF_000581295	non-GC1	506
GCF_000581315	non-GC1	16
GCF_000581335	non-GC1	2
GCF_000581355	non-GC1	2
GCF_000581375	non-GC1	2
GCF_000581395	non-GC1	2
GCF_000581415	non-GC1	2
GCF_000581455	non-GC1	2
GCF_000581475	non-GC1	416
GCF_000581495	non-GC1	2
GCF_000581515	non-GC1	670
GCF_000581535	non-GC1	2
GCF_000581555	non-GC1	522
GCF_000581575	non-GC1	2
GCF_000581595	non-GC1	650
GCF_000581615	non-GC1	2
GCF_000581635	non-GC1	2
GCF_000581655	non-GC1	2
**GCF_000581675**	**non-GC1**	**2**
GCF_000581695	non-GC1	2
GCF_000581735	non-GC1	2

GCF_000581755	non-GC1	78
GCF_000581775	non-GC1	505
GCF_000581815	non-GC1	2
GCF_000581855	non-GC1	533
GCF_000581875	non-GC1	154
GCF_000581915	non-GC1	16
GCF_000581935	non-GC1	2
GCF_000581955	non-GC1	16
GCF_000581975	non-GC1	78
GCF_000582015	non-GC1	2
GCF_000582035	non-GC1	514
GCF_000582115	non-GC1	2
GCF_000582155	non-GC1	10
GCF_000582175	non-GC1	32
GCF_000582215	non-GC1	2
GCF_000582275	non-GC1	499
GCF_000582295	non-GC1	514
GCF_000582335	non-GC1	2
GCF_000582355	non-GC1	108
GCF_000582945	non-GC1	2
GCF_000582965	non-GC1	16
GCF_000583235	non-GC1	2
GCF_000583255	non-GC1	2
GCF_000583275	non-GC1	499
GCF_000583295	non-GC1	2
GCF_000583315	non-GC1	2
GCF_000583335	non-GC1	2
GCF_000583355	non-GC1	2
GCF_000583415	non-GC1	500
GCF_000583435	non-GC1	2
GCF_000583455	non-GC1	2
GCF_000583475	non-GC1	526
GCF_000583495	non-GC1	525
GCF_000583515	non-GC1	2
GCF_000583575	non-GC1	2
GCF_000583595	non-GC1	2
GCF_000584035	non-GC1	195
GCF_000584075	non-GC1	2
GCF_000584095	non-GC1	535
GCF_000584115	non-GC1	2
GCF_000584135	non-GC1	2
GCF_000584155	non-GC1	2
GCF_000584175	non-GC1	2
GCF_000584195	non-GC1	2
GCF_000584235	non-GC1	2
GCF_000584275	non-GC1	500

GCF_000584295	non-GC1	499
GCF_000584315	non-GC1	499
GCF_000584335	non-GC1	10
GCF_000584355	non-GC1	651
GCF_000584595	non-GC1	500
GCF_000584635	non-GC1	500
GCF_000584655	non-GC1	2
GCF_000584675	non-GC1	2
GCF_000584695	non-GC1	16
GCF_000584715	non-GC1	54
GCF_000584735	non-GC1	2
GCF_000584755	non-GC1	671
GCF_000584775	non-GC1	2
GCF_000584835	non-GC1	2
GCF_000584855	non-GC1	499
GCF_000584875	non-GC1	2
GCF_000584895	non-GC1	2
GCF_000587875	non-GC1	2
GCF_000587895	non-GC1	2
GCF_000587915	non-GC1	2
GCF_000587935	non-GC1	2
GCF_000587955	non-GC1	499
GCF_000587975	non-GC1	2
GCF_000588035	non-GC1	532
GCF_000588055	non-GC1	2
GCF_000588075	non-GC1	416
GCF_000588115	non-GC1	500
GCF_000588135	non-GC1	652
GCF_000588155	non-GC1	2
GCF_000588175	non-GC1	2
GCF_000588195	non-GC1	652
GCF_000588215	non-GC1	2
GCF_000588235	non-GC1	499
GCF_000588275	non-GC1	563
GCF_000588295	non-GC1	2
GCF_000588315	non-GC1	2
GCF_000588355	non-GC1	521
GCF_000588375	non-GC1	2
GCF_000588435	non-GC1	2
GCF_000588455	non-GC1	2
GCF_000588475	non-GC1	652
GCF_000588495	non-GC1	2
GCF_000588555	non-GC1	2
GCF_000588615	non-GC1	499
GCF_000588635	non-GC1	2
GCF_000588655	non-GC1	2

GCF_000588675	non-GC1	10
GCF_000588695	non-GC1	2
GCF_000588735	non-GC1	2
GCF_000588755	non-GC1	285
GCF_000588775	non-GC1	499
GCF_000588795	non-GC1	2
GCF_000588815	non-GC1	2
GCF_000588895	non-GC1	2
GCF_000588915	non-GC1	2
GCF_000588935	non-GC1	2
GCF_000588955	non-GC1	2
GCF_000588975	non-GC1	2
GCF_000588995	non-GC1	2
GCF_000589015	non-GC1	2
GCF_000589035	non-GC1	2
GCF_000589055	non-GC1	2
GCF_000589075	non-GC1	2
GCF_000589095	non-GC1	2
GCF_000589195	non-GC1	2
GCF_000589275	non-GC1	2
GCF_000589295	non-GC1	2
GCF_000589315	non-GC1	2
GCF_000589355	non-GC1	2
GCF_000589375	non-GC1	2
GCF_000589395	non-GC1	2
GCF_000589415	non-GC1	2
GCF_000589435	non-GC1	2
GCF_000589455	non-GC1	2
GCF_000589475	non-GC1	2
GCF_000589495	non-GC1	2
GCF_000589515	non-GC1	2
GCF_000589535	non-GC1	2
GCF_000589555	non-GC1	2
GCF_000589575	non-GC1	2
GCF_000589595	non-GC1	2
GCF_000589615	non-GC1	2
GCF_000589635	non-GC1	661
GCF_000589655	non-GC1	2
GCF_000589675	non-GC1	2
GCF_000589695	non-GC1	2
GCF_000589715	non-GC1	2
GCF_000589735	non-GC1	2
GCF_000589755	non-GC1	2
GCF_000589775	non-GC1	2
GCF_000589795	non-GC1	2
GCF_000589815	non-GC1	2

GCF_000589835	non-GC1	2
GCF_000591575	non-GC1	2
GCF_000591595	non-GC1	2
GCF_000591615	non-GC1	2
GCF_000591635	non-GC1	2
GCF_000591655	non-GC1	2
GCF_000591675	non-GC1	2
GCF_000591695	non-GC1	2
GCF_000591715	non-GC1	2
GCF_000591735	non-GC1	2
GCF_000591755	non-GC1	2
GCF_000591775	non-GC1	2
GCF_000591795	non-GC1	2
GCF_000591815	non-GC1	2
GCF_000591835	non-GC1	2
GCF_000591855	non-GC1	2
GCF_000591875	non-GC1	2
GCF_000591895	non-GC1	2
GCF_000591915	non-GC1	2
GCF_000591935	non-GC1	2
GCF_000591955	non-GC1	2
GCF_000591975	non-GC1	2
GCF_000591995	non-GC1	2
GCF_000592015	non-GC1	2
GCF_000592035	non-GC1	2
GCF_000592055	non-GC1	2
GCF_000592075	non-GC1	2
GCF_000592095	non-GC1	2
GCF_000592115	non-GC1	2
GCF_000592135	non-GC1	2
GCF_000592155	non-GC1	2
GCF_000592175	non-GC1	2
GCF_000592195	non-GC1	2
GCF_000592215	non-GC1	2
GCF_000592235	non-GC1	2
GCF_000592255	non-GC1	2
GCF_000592275	non-GC1	2
GCF_000592295	non-GC1	2
GCF_000592315	non-GC1	2
GCF_000592335	non-GC1	2
GCF_000592355	non-GC1	2
GCF_000592375	non-GC1	2
GCF_000592395	non-GC1	2
GCF_000592415	non-GC1	2
GCF_000592435	non-GC1	2
GCF_000592455	non-GC1	2

GCF_000592475	non-GC1	2
GCF_000592495	non-GC1	2
GCF_000592515	non-GC1	2
GCF_000592535	non-GC1	2
GCF_000592555	non-GC1	2
GCF_000592575	non-GC1	2
GCF_000592595	non-GC1	2
GCF_000592615	non-GC1	2
GCF_000592635	non-GC1	2
GCF_000592655	non-GC1	662
GCF_000592675	non-GC1	2
GCF_000592695	non-GC1	2
GCF_000592715	non-GC1	2
GCF_000592735	non-GC1	2
GCF_000592755	non-GC1	2
GCF_000600375	non-GC1	2
GCF_000600395	non-GC1	2
GCF_000600415	non-GC1	2
GCF_000600435	non-GC1	2
GCF_000600455	non-GC1	2
GCF_000600475	non-GC1	2
GCF_000600495	non-GC1	2
GCF_000600515	non-GC1	2
GCF_000600535	non-GC1	2
GCF_000600555	non-GC1	2
GCF_000600575	non-GC1	2
GCF_000600595	non-GC1	2
GCF_000600615	non-GC1	2
GCF_000600635	non-GC1	2
GCF_000600655	non-GC1	2
GCF_000600675	non-GC1	2
GCF_000600695	non-GC1	2
GCF_000600715	non-GC1	2
GCF_000600735	non-GC1	417
GCF_000600755	non-GC1	656
GCF_000600775	non-GC1	417
GCF_000600795	non-GC1	416
GCF_000600815	non-GC1	657
GCF_000600835	non-GC1	658
GCF_000600855	non-GC1	659
GCF_000600875	non-GC1	416
GCF_000600895	non-GC1	417
GCF_000600915	non-GC1	2
GCF_000600935	non-GC1	2
GCF_000611045	non-GC1	415
GCF_000611065	non-GC1	415

GCF_000611085	non-GC1	415
GCF_000611105	non-GC1	415
GCF_000611165	non-GC1	415
GCF_000611185	non-GC1	415
GCF_000611205	non-GC1	416
GCF_000611225	non-GC1	417
GCF_000611245	non-GC1	417
GCF_000611265	non-GC1	2
GCF_000611285	non-GC1	514
GCF_000611305	non-GC1	514
GCF_000611325	non-GC1	2
GCF_000611345	non-GC1	2
GCF_000611365	non-GC1	416
GCF_000611385	non-GC1	660
GCF_000611405	non-GC1	2
GCF_000611425	non-GC1	2
GCF_000611445	non-GC1	2
GCF_000611465	non-GC1	417
GCF_000611485	non-GC1	514
GCF_000611505	non-GC1	2
GCF_000611525	non-GC1	2
GCF_000611545	non-GC1	415
GCF_000611565	non-GC1	2
GCF_000627335	non-GC1	2
GCF_000627355	non-GC1	2
GCF_000627395	non-GC1	2
GCF_000627415	non-GC1	2
GCF_000627495	non-GC1	2
GCF_000627515	non-GC1	2
GCF_000627535	non-GC1	2
GCF_000627555	non-GC1	2
GCF_000627575	non-GC1	2
GCF_000627595	non-GC1	2
GCF_000627615	non-GC1	2
GCF_000627635	non-GC1	416
GCF_000627655	non-GC1	416
GCF_000627695	non-GC1	673
GCF_000627715	non-GC1	674
GCF_000627735	non-GC1	2
GCF_000627755	non-GC1	500
GCF_000627775	non-GC1	514
GCF_000671415	non-GC1	514
GCF_000671475	non-GC1	514
GCF_000671495	non-GC1	2
GCF_000671515	non-GC1	2
GCF_000671535	non-GC1	2

GCF_000671555	non-GC1	2
GCF_000671575	non-GC1	2
GCF_000671595	non-GC1	514
GCF_000671615	non-GC1	514
GCF_000671635	non-GC1	514
GCF_000671655	non-GC1	2
GCF_000671675	non-GC1	500
GCF_000681575	non-GC1	336
GCF_000681595	non-GC1	1143
GCF_000681615	non-GC1	417
GCF_000681635	non-GC1	499
GCF_000681655	non-GC1	2
GCF_000681675	non-GC1	2
GCF_000681695	non-GC1	2
GCF_000681735	non-GC1	2
GCF_000681755	non-GC1	500
GCF_000681775	non-GC1	514
GCF_000681795	non-GC1	514
GCF_000681835	non-GC1	2
GCF_000681855	non-GC1	2
GCF_000681875	non-GC1	2
GCF_000681895	non-GC1	2
GCF_000681935	non-GC1	2
GCF_000681955	non-GC1	2
GCF_000681975	non-GC1	2
GCF_000681995	non-GC1	2
GCF_000682015	non-GC1	500
GCF_000682035	non-GC1	500
GCF_000682055	non-GC1	514
GCF_000682075	non-GC1	2
GCF_000682095	non-GC1	501
GCF_000682115	non-GC1	2
GCF_000682135	non-GC1	2
GCF_000682175	non-GC1	2
GCF_000682215	non-GC1	499
GCF_000682235	non-GC1	2
GCF_000682255	non-GC1	499
GCF_000682275	non-GC1	514
GCF_000682335	non-GC1	71
GCF_000682375	non-GC1	499
GCF_000682415	non-GC1	56
GCF_000682435	non-GC1	126
GCF_000682475	non-GC1	10
GCF_000682495	non-GC1	2
GCF_000682535	non-GC1	668
GCF_000682555	non-GC1	2

GCF_000682575	non-GC1	2
GCF_000682595	non-GC1	664
GCF_000682655	non-GC1	2
GCF_000695855	non-GC1	2
GCF_000708775	non-GC1	499
GCF_000708795	non-GC1	499
GCF_000722965	non-GC1	40
GCF_000722975	non-GC1	40
GCF_000722985	non-GC1	2
GCF_000723045	non-GC1	2
GCF_000723065	non-GC1	2
GCF_000723075	non-GC1	2
GCF_000723125	non-GC1	2
GCF_000723145	non-GC1	2
GCF_000731965	non-GC1	621
GCF_000734775	non-GC1	2
GCF_000738845	non-GC1	499
GCF_000746605	non-GC1	638
GCF_000746645	non-GC1	79
GCF_000757665	non-GC1	79
GCF_000759395	non-GC1	271
GCF_000760395	non-GC1	40
GCF_000760415	non-GC1	40
GCF_000760435	non-GC1	52
GCF_000760455	non-GC1	40
GCF_000760475	non-GC1	52
GCF_000761175	non-GC1	79
GCF_000766105	non-GC1	2
GCF_000770805	non-GC1	40
GCF_000770815	non-GC1	2
GCF_000770885	non-GC1	2
GCF_000770905	non-GC1	2
GCF_000775835	non-GC1	52
GCF_000775855	non-GC1	52
GCF_000775875	non-GC1	40
GCF_000786735	non-GC1	10
GCF_000787015	non-GC1	119
GCF_000787215	non-GC1	2
GCF_000787225	non-GC1	2
GCF_000787235	non-GC1	2
GCF_000787275	non-GC1	40
GCF_000787295	non-GC1	2
GCF_000787315	non-GC1	2
GCF_000787355	non-GC1	2
GCF_000802895	non-GC1	2
GCF_000805035	non-GC1	2

GCF_000805365	non-GC1	1184
GCF_000805515	non-GC1	215
GCF_000805525	non-GC1	2
GCF_000805535	non-GC1	653
GCF_000805545	non-GC1	2
GCF_000805595	non-GC1	2
GCF_000805615	non-GC1	2
GCF_000805635	non-GC1	2
GCF_000808765	non-GC1	2
GCF_000808775	non-GC1	2
GCF_000808785	non-GC1	2
GCF_000808815	non-GC1	2
GCF_000808845	non-GC1	2
GCF_000808885	non-GC1	2
GCF_000808905	non-GC1	2
GCF_000808925	non-GC1	2
GCF_000808945	non-GC1	2
GCF_000808985	non-GC1	2
GCF_000809005	non-GC1	2
GCF_000809045	non-GC1	2
GCF_000809065	non-GC1	2
GCF_000809085	non-GC1	2
GCF_000809205	non-GC1	2
GCF_000809215	non-GC1	2
GCF_000809225	non-GC1	2
GCF_000809235	non-GC1	2
GCF_000809285	non-GC1	2
GCF_000809305	non-GC1	2
GCF_000809325	non-GC1	2
GCF_000809345	non-GC1	2
GCF_000809365	non-GC1	2
GCF_000809385	non-GC1	2
GCF_000809405	non-GC1	2
GCF_000809425	non-GC1	2
GCF_000809445	non-GC1	2
GCF_000809465	non-GC1	2
GCF_000809485	non-GC1	2
GCF_000809505	non-GC1	78
GCF_000809525	non-GC1	2
GCF_000809545	non-GC1	2
GCF_000809565	non-GC1	2
GCF_000809585	non-GC1	2
GCF_000809605	non-GC1	2
GCF_000809625	non-GC1	2
GCF_000809645	non-GC1	2
GCF_000809665	non-GC1	2

GCF_000809685	non-GC1	2
GCF_000809705	non-GC1	2
GCF_000809725	non-GC1	2
GCF_000809745	non-GC1	2
GCF_000809765	non-GC1	2
GCF_000809785	non-GC1	2
GCF_000809805	non-GC1	2
GCF_000809825	non-GC1	2
GCF_000809845	non-GC1	79
GCF_000809865	non-GC1	79
GCF_000809885	non-GC1	2
GCF_000809905	non-GC1	79
GCF_000809925	non-GC1	1088
GCF_000809945	non-GC1	2
GCF_000809965	non-GC1	1088
GCF_000809985	non-GC1	2
GCF_000810005	non-GC1	1088
GCF_000810025	non-GC1	2
GCF_000810045	non-GC1	2
GCF_000810065	non-GC1	2
GCF_000810085	non-GC1	2
GCF_000810145	non-GC1	2
GCF_000810165	non-GC1	2
GCF_000810185	non-GC1	2
GCF_000810205	non-GC1	2
GCF_000810245	non-GC1	2
GCF_000810265	non-GC1	2
GCF_000810285	non-GC1	2
GCF_000810305	non-GC1	2
GCF_000810325	non-GC1	2
GCF_000810345	non-GC1	2
GCF_000810365	non-GC1	2
GCF_000810385	non-GC1	2
GCF_000810405	non-GC1	2
GCF_000810425	non-GC1	2
GCF_000810445	non-GC1	2
GCF_000810465	non-GC1	2
GCF_000810485	non-GC1	2
GCF_000810505	non-GC1	2
GCF_000810525	non-GC1	2
GCF_000810545	non-GC1	2
GCF_000810565	non-GC1	2
GCF_000810585	non-GC1	2
GCF_000810605	non-GC1	2
GCF_000810625	non-GC1	2
GCF_000810645	non-GC1	2

GCF_000810665	non-GC1	2
GCF_000810685	non-GC1	2
GCF_000810725	non-GC1	2
GCF_000810745	non-GC1	2
GCF_000810765	non-GC1	2
GCF_000810825	non-GC1	2
GCF_000810835	non-GC1	2
GCF_000810865	non-GC1	2
GCF_000810875	non-GC1	2
GCF_000810905	non-GC1	2
GCF_000810915	non-GC1	2
GCF_000810935	non-GC1	2
GCF_000810945	non-GC1	2
GCF_000810985	non-GC1	2
GCF_000810995	non-GC1	250
GCF_000811005	non-GC1	2
GCF_000811015	non-GC1	2
GCF_000811065	non-GC1	2
GCF_000811085	non-GC1	2
GCF_000811105	non-GC1	2
GCF_000811145	non-GC1	2
GCF_000811165	non-GC1	2
GCF_000811185	non-GC1	2
GCF_000811205	non-GC1	2
GCF_000811225	non-GC1	2
GCF_000811245	non-GC1	2
GCF_000811265	non-GC1	2
GCF_000811285	non-GC1	2
GCF_000811305	non-GC1	2
GCF_000811325	non-GC1	2
GCF_000811345	non-GC1	2
GCF_000811365	non-GC1	2
GCF_000811405	non-GC1	2
GCF_000811485	non-GC1	2
GCF_000811505	non-GC1	2
GCF_000811525	non-GC1	2
GCF_000811545	non-GC1	2
GCF_000811565	non-GC1	2
**GCF_000811585**	**non-GC1**	**2**
GCF_000811605	non-GC1	2
GCF_000811625	non-GC1	2
GCF_000811645	non-GC1	2
GCF_000811665	non-GC1	2
GCF_000811685	non-GC1	2
GCF_000811705	non-GC1	2
GCF_000811725	non-GC1	2

GCF_000811745	non-GC1	2
GCF_000811765	non-GC1	2
GCF_000811785	non-GC1	2
GCF_000811805	non-GC1	10
GCF_000811825	non-GC1	2
GCF_000811865	non-GC1	2
GCF_000811885	non-GC1	1088
GCF_000811905	non-GC1	2
GCF_000811925	non-GC1	2
GCF_000811945	non-GC1	2
GCF_000814345	non-GC1	464
GCF_000828795	non-GC1	2
GCF_000828935	non-GC1	622
GCF_000836075	non-GC1	52
GCF_000876055	non-GC1	40
GCF_000876075	non-GC1	34
GCF_000876095	non-GC1	52
GCF_000931755	non-GC1	52
GCF_000939395	non-GC1	2
GCF_000939405	non-GC1	2
GCF_000939415	non-GC1	2
GCF_000939425	non-GC1	2
GCF_000939475	non-GC1	2
GCF_000939485	non-GC1	2
GCF_000939495	non-GC1	2
GCF_000939535	non-GC1	2
GCF_000939555	non-GC1	2
GCF_000939565	non-GC1	2
GCF_000939575	non-GC1	2
GCF_000939615	non-GC1	2
GCF_000939635	non-GC1	2
GCF_000949035	non-GC1	2
GCF_000949045	non-GC1	2
GCF_000949055	non-GC1	2
GCF_000949065	non-GC1	2
GCF_000949115	non-GC1	2
GCF_000949165	non-GC1	250
GCF_000949175	non-GC1	2
GCF_000949185	non-GC1	250
GCF_000949195	non-GC1	2
GCF_000966495	non-GC1	2
GCF_000966505	non-GC1	16
GCF_000966515	non-GC1	2
GCF_000969285	non-GC1	2
GCF_000969365	non-GC1	2
GCF_000969485	non-GC1	654

GCF_000972525	non-GC1	10
GCF_000972625	non-GC1	10
GCF_000988155	non-GC1	15
GCF_001007685	non-GC1	402
GCF_001007705	non-GC1	25
GCF_001007725	non-GC1	25
GCF_001007745	non-GC1	25
GCF_001007765	non-GC1	25
GCF_001007775	non-GC1	25
GCF_001007815	non-GC1	25
GCF_001008125	non-GC1	2
GCF_001026965	non-GC1	2
GCF_001027885	non-GC1	2
GCF_001052015	non-GC1	2
GCF_001052325	non-GC1	2
GCF_001052675	non-GC1	2
GCF_001053695	non-GC1	2
GCF_001053755	non-GC1	1426
GCF_001059495	non-GC1	2
GCF_001077555	non-GC1	1545
GCF_001077655	non-GC1	10
GCF_001077675	non-GC1	437
GCF_001077965	non-GC1	1549
GCF_001261895	non-GC1	267
GCF_001276055	non-GC1	2
GCF_001276085	non-GC1	10
GCF_001292715	non-GC1	2
GCF_001400975	non-GC1	492
GCF_001414565	non-GC1	79
GCF_001414655	non-GC1	406
GCF_001414735	non-GC1	79
GCF_001414785	non-GC1	79
GCF_001414805	non-GC1	79
GCF_001414865	non-GC1	2
GCF_001414905	non-GC1	79
GCF_001414915	non-GC1	498
GCF_001414935	non-GC1	79
GCF_001415015	non-GC1	79
GCF_001415065	non-GC1	49
GCF_001415095	non-GC1	2
GCF_001415165	non-GC1	406
GCF_001415295	non-GC1	79
GCF_001415325	non-GC1	79
GCF_001415345	non-GC1	79
GCF_001415355	non-GC1	79
GCF_001415385	non-GC1	79

GCF_001415435	non-GC1	103
GCF_001415505	non-GC1	499
GCF_001415515	non-GC1	499
GCF_001415595	non-GC1	79
GCF_001415615	non-GC1	499
GCF_001415645	non-GC1	2
GCF_001415695	non-GC1	2
GCF_001415805	non-GC1	406
GCF_001415875	non-GC1	79
GCF_001415985	non-GC1	406
GCF_001416075	non-GC1	2
GCF_001416095	non-GC1	2
GCF_001416115	non-GC1	1337
GCF_001416145	non-GC1	499
GCF_001416155	non-GC1	499
GCF_001416225	non-GC1	2
GCF_001416235	non-GC1	406
GCF_001416275	non-GC1	2
GCF_001416305	non-GC1	499
GCF_001416545	non-GC1	406
GCF_001416585	non-GC1	406
GCF_001416625	non-GC1	250
GCF_001416715	non-GC1	79
GCF_001420505	non-GC1	2
GCF_001432265	non-GC1	85
GCF_001432375	non-GC1	406
GCF_001432385	non-GC1	79
GCF_001432425	non-GC1	372
GCF_001432455	non-GC1	6
GCF_001432545	non-GC1	25
GCF_001432625	non-GC1	79
GCF_001432665	non-GC1	78
GCF_001432685	non-GC1	1022
GCF_001432705	non-GC1	1022
GCF_001432745	non-GC1	497
GCF_001432755	non-GC1	2
GCF_001432785	non-GC1	406
GCF_001432825	non-GC1	32
GCF_001432845	non-GC1	78
GCF_001432965	non-GC1	79
GCF_001432975	non-GC1	79
GCF_001433045	non-GC1	2
GCF_001433075	non-GC1	406
GCF_001433205	non-GC1	2
GCF_001433245	non-GC1	79
GCF_001433275	non-GC1	79

GCF_001433335	non-GC1	79
GCF_001433655	non-GC1	79
GCF_001433675	non-GC1	2
GCF_001433695	non-GC1	79
GCF_001441355	non-GC1	10
GCF_001441405	non-GC1	10
GCF_001441415	non-GC1	10
GCF_001441455	non-GC1	10
GCF_001441465	non-GC1	10
GCF_001441475	non-GC1	10
GCF_001441485	non-GC1	10
GCF_001441545	non-GC1	10
GCF_001441555	non-GC1	10
GCF_001441565	non-GC1	10
GCF_001441625	non-GC1	10
GCF_001441645	non-GC1	10
GCF_001442575	non-GC1	2
GCF_001446715	non-GC1	742
GCF_001457535	non-GC1	126
GCF_001471855	non-GC1	16
GCF_001500225	non-GC1	15
GCF_001514375	non-GC1	2
GCF_001517645	non-GC1	126
GCF_001518715	non-GC1	2
GCF_001531135	non-GC1	79
GCF_001531205	non-GC1	2
GCF_001531265	non-GC1	79
GCF_001531455	non-GC1	2
GCF_001541025	non-GC1	2
GCF_001541035	non-GC1	2
GCF_001541045	non-GC1	2
GCF_001541425	non-GC1	187
GCF_001543995	non-GC1	2
GCF_001544395	non-GC1	126
GCF_001573065	non-GC1	2
GCF_001573085	non-GC1	2
GCF_001573105	non-GC1	215
GCF_001573125	non-GC1	2
GCF_001576615	non-GC1	2
GCF_001578145	non-GC1	23
GCF_001593425	non-GC1	437
GCF_001617825	non-GC1	2
GCF_001617895	non-GC1	2
GCF_001618125	non-GC1	2
GCF_001624975	non-GC1	2
GCF_001636235	non-GC1	422

GCF_001640065	non-GC1	604
GCF_001640075	non-GC1	2
GCF_001649595	non-GC1	107
GCF_001649615	non-GC1	317
GCF_001649625	non-GC1	317
GCF_001649675	non-GC1	79
GCF_001649705	non-GC1	79
GCF_001649765	non-GC1	79
GCF_001649795	non-GC1	79
GCF_001649805	non-GC1	79
GCF_001649815	non-GC1	107
GCF_001649835	non-GC1	79
GCF_001649875	non-GC1	79
GCF_001649895	non-GC1	79
GCF_001649955	non-GC1	79
GCF_001649975	non-GC1	317
GCF_001664565	non-GC1	2
GCF_001664575	non-GC1	2
GCF_001664585	non-GC1	2
GCF_001664595	non-GC1	2
GCF_001664645	non-GC1	2
GCF_001664655	non-GC1	2
GCF_001664665	non-GC1	2
GCF_001664685	non-GC1	1088
GCF_001664725	non-GC1	2
GCF_001664735	non-GC1	2
GCF_001664755	non-GC1	2
GCF_001664765	non-GC1	406
GCF_001664805	non-GC1	2
GCF_001664815	non-GC1	250
GCF_001664835	non-GC1	2
GCF_001664855	non-GC1	2
GCF_001664885	non-GC1	2
GCF_001664895	non-GC1	2
GCF_001664905	non-GC1	2
GCF_001664915	non-GC1	2
GCF_001664965	non-GC1	2
GCF_001664975	non-GC1	2
GCF_001664985	non-GC1	2
GCF_001665005	non-GC1	499
GCF_001665045	non-GC1	2
GCF_001665055	non-GC1	2
GCF_001665065	non-GC1	2
GCF_001665095	non-GC1	2
GCF_001665125	non-GC1	2
GCF_001665145	non-GC1	406

GCF_001665155	non-GC1	632
GCF_001665175	non-GC1	2
GCF_001665205	non-GC1	2
GCF_001665215	non-GC1	2
GCF_001665285	non-GC1	2
GCF_001665985	non-GC1	2
GCF_001666065	non-GC1	2
GCF_001666075	non-GC1	2
GCF_001666085	non-GC1	2
GCF_001666105	non-GC1	2
GCF_001666145	non-GC1	2
GCF_001666155	non-GC1	2
GCF_001666175	non-GC1	2
GCF_001666195	non-GC1	2
GCF_001666225	non-GC1	250
GCF_001666235	non-GC1	2
GCF_001666255	non-GC1	2
GCF_001666275	non-GC1	2
GCF_001666305	non-GC1	2
GCF_001666315	non-GC1	2
GCF_001666325	non-GC1	2
GCF_001666365	non-GC1	1088
GCF_001666385	non-GC1	2
GCF_001666395	non-GC1	2
GCF_001666405	non-GC1	632
GCF_001666425	non-GC1	2
GCF_001666465	non-GC1	2
GCF_001666475	non-GC1	2
GCF_001666485	non-GC1	2
GCF_001666515	non-GC1	2
GCF_001666545	non-GC1	2
GCF_001666555	non-GC1	2
GCF_001666565	non-GC1	2
GCF_001666575	non-GC1	2
GCF_001666625	non-GC1	2
GCF_001666635	non-GC1	2
GCF_001666645	non-GC1	2
GCF_001666665	non-GC1	2
GCF_001666705	non-GC1	2
GCF_001666715	non-GC1	2
GCF_001668445	non-GC1	2
GCF_001668465	non-GC1	2
GCF_001668475	non-GC1	2
GCF_001668515	non-GC1	2
GCF_001668535	non-GC1	2
GCF_001668555	non-GC1	2

GCF_001668585	non-GC1	2
GCF_001668645	non-GC1	2
GCF_001668755	non-GC1	2
GCF_001668765	non-GC1	2
GCF_001668775	non-GC1	2
GCF_001668815	non-GC1	2
GCF_001668835	non-GC1	2
GCF_001668855	non-GC1	2
GCF_001668865	non-GC1	2
GCF_001668875	non-GC1	499
GCF_001668915	non-GC1	2
GCF_001668935	non-GC1	2
GCF_001668945	non-GC1	2
GCF_001668955	non-GC1	2
GCF_001668995	non-GC1	2
GCF_001669005	non-GC1	2
GCF_001669025	non-GC1	2
GCF_001669035	non-GC1	2
GCF_001669075	non-GC1	2
GCF_001669095	non-GC1	2
GCF_001669105	non-GC1	2
GCF_001669115	non-GC1	2
GCF_001669145	non-GC1	2
GCF_001669175	non-GC1	2
GCF_001669185	non-GC1	2
GCF_001669195	non-GC1	2
GCF_001669225	non-GC1	2
GCF_001669255	non-GC1	2
GCF_001669265	non-GC1	2
GCF_001669295	non-GC1	2
GCF_001669305	non-GC1	2
GCF_001669355	non-GC1	2
GCF_001669365	non-GC1	2
GCF_001669415	non-GC1	2
GCF_001669425	non-GC1	2
GCF_001669435	non-GC1	2
GCF_001669455	non-GC1	2
GCF_001669495	non-GC1	2
GCF_001669505	non-GC1	2
GCF_001669525	non-GC1	2
GCF_001669535	non-GC1	2
GCF_001669575	non-GC1	2
GCF_001669595	non-GC1	2
GCF_001669605	non-GC1	2
GCF_001669635	non-GC1	2
GCF_001669645	non-GC1	2

GCF_001669675	non-GC1	2
GCF_001669695	non-GC1	2
GCF_001669705	non-GC1	2
GCF_001669735	non-GC1	2
GCF_001673795	non-GC1	2
GCF_001673805	non-GC1	2
GCF_001673815	non-GC1	2
GCF_001673825	non-GC1	2
GCF_001673875	non-GC1	2
GCF_001673895	non-GC1	2
GCF_001673905	non-GC1	2
GCF_001673915	non-GC1	2
GCF_001673955	non-GC1	2
GCF_001673975	non-GC1	2
GCF_001673985	non-GC1	2
GCF_001674015	non-GC1	2
GCF_001674045	non-GC1	2
GCF_001674075	non-GC1	2
GCF_001674085	non-GC1	2
GCF_001674115	non-GC1	2
GCF_001674135	non-GC1	2
GCF_001674155	non-GC1	2
GCF_001674165	non-GC1	2
GCF_001674185	non-GC1	250
GCF_001674215	non-GC1	2
GCF_001674235	non-GC1	2
GCF_001674245	non-GC1	2
GCF_001674255	non-GC1	2
GCF_001674295	non-GC1	2
GCF_001674315	non-GC1	2
GCF_001674325	non-GC1	2
GCF_001674345	non-GC1	2
GCF_001674365	non-GC1	2
GCF_001674395	non-GC1	2
GCF_001674415	non-GC1	2
GCF_001674425	non-GC1	2
GCF_001674435	non-GC1	2
GCF_001674475	non-GC1	2
GCF_001674485	non-GC1	632
GCF_001674505	non-GC1	2
GCF_001674515	non-GC1	2
GCF_001674555	non-GC1	2
GCF_001674575	non-GC1	2
GCF_001674585	non-GC1	2
GCF_001674605	non-GC1	2
GCF_001674635	non-GC1	2

GCF_001674655	non-GC1	2
GCF_001674665	non-GC1	2
GCF_001674695	non-GC1	1079
GCF_001674705	non-GC1	2
GCF_001674735	non-GC1	2
GCF_001674755	non-GC1	2
GCF_001674765	non-GC1	2
GCF_001674785	non-GC1	2
GCF_001674805	non-GC1	2
GCF_001674835	non-GC1	2
GCF_001674845	non-GC1	2
GCF_001674865	non-GC1	2
GCF_001674885	non-GC1	2
GCF_001674915	non-GC1	2
GCF_001675265	non-GC1	111
GCF_001680105	non-GC1	2
GCF_001693105	non-GC1	943
GCF_001693125	non-GC1	821
GCF_001693185	non-GC1	512
GCF_001693195	non-GC1	309
GCF_001693205	non-GC1	80
GCF_001700395	non-GC1	2
GCF_001708025	non-GC1	2
GCF_001721365	non-GC1	437
GCF_001721705	non-GC1	1546
GCF_001756815	non-GC1	107
GCF_001758065	non-GC1	32
GCF_001758085	non-GC1	15
GCF_001758125	non-GC1	107
GCF_001758135	non-GC1	79
GCF_001758165	non-GC1	107
GCF_001806345	non-GC1	2
GCF_001806365	non-GC1	2
GCF_001806385	non-GC1	2
GCF_001806405	non-GC1	2
GCF_001806425	non-GC1	2
GCF_001806445	non-GC1	2
GCF_001806465	non-GC1	2
GCF_001806485	non-GC1	2
GCF_001855175	non-GC1	2
GCF_001861695	non-GC1	2
GCF_001861735	non-GC1	2
GCF_001861745	non-GC1	2
GCF_001861775	non-GC1	2
GCF_001861785	non-GC1	2
GCF_001861855	non-GC1	2

GCF_001861865	non-GC1	2
GCF_001861895	non-GC1	2
GCF_001861905	non-GC1	2
GCF_001861935	non-GC1	10
GCF_001861955	non-GC1	10
GCF_001861975	non-GC1	2
GCF_001862035	non-GC1	2
GCF_001862055	non-GC1	2
GCF_001862065	non-GC1	2
GCF_001862075	non-GC1	2
GCF_001862105	non-GC1	2
GCF_001862175	non-GC1	2
GCF_001862215	non-GC1	2
GCF_001862225	non-GC1	2
GCF_001862235	non-GC1	2
GCF_001862265	non-GC1	2
GCF_001862295	non-GC1	2
GCF_001862305	non-GC1	2
GCF_001862335	non-GC1	46
GCF_001862345	non-GC1	2
GCF_001862375	non-GC1	2
GCF_001862385	non-GC1	880
GCF_001862395	non-GC1	2
GCF_001862425	non-GC1	2
GCF_001862455	non-GC1	2
GCF_001862485	non-GC1	2
GCF_001862505	non-GC1	2
GCF_001862535	non-GC1	2
GCF_001862555	non-GC1	2
GCF_001862565	non-GC1	2
GCF_001862615	non-GC1	2
GCF_001862645	non-GC1	2
GCF_001862665	non-GC1	2
GCF_001862715	non-GC1	2
GCF_001862725	non-GC1	338
GCF_001862735	non-GC1	2
GCF_001862775	non-GC1	2
GCF_001862795	non-GC1	23
GCF_001862825	non-GC1	2
GCF_001862855	non-GC1	2
GCF_001862875	non-GC1	2
GCF_001862885	non-GC1	2
GCF_001862895	non-GC1	2
GCF_001862955	non-GC1	2
GCF_001862975	non-GC1	2
GCF_001863015	non-GC1	2

GCF_001863035	non-GC1	2
GCF_001863055	non-GC1	2
GCF_001863065	non-GC1	2
GCF_001863085	non-GC1	2
GCF_001863115	non-GC1	2
GCF_001863135	non-GC1	2
GCF_001863145	non-GC1	2
GCF_001863165	non-GC1	2
GCF_001863205	non-GC1	2
GCF_001863225	non-GC1	2
GCF_001863295	non-GC1	2
GCF_001863315	non-GC1	2
GCF_001863335	non-GC1	2
GCF_001863345	non-GC1	2
GCF_001863395	non-GC1	2
GCF_001863405	non-GC1	2
GCF_001863415	non-GC1	46
GCF_001863455	non-GC1	2
GCF_001863475	non-GC1	2
GCF_001863485	non-GC1	46
GCF_001863535	non-GC1	2
GCF_001863545	non-GC1	2
GCF_001863555	non-GC1	2
GCF_001863575	non-GC1	2
GCF_001863615	non-GC1	2
GCF_001863625	non-GC1	2
GCF_001863665	non-GC1	2
GCF_001863695	non-GC1	2
GCF_001863705	non-GC1	875
GCF_001863725	non-GC1	46
GCF_001863745	non-GC1	46
GCF_001863775	non-GC1	2
GCF_001863785	non-GC1	878
**GCF_001863815**	**non-GC1**	**2**
GCF_001863835	non-GC1	46
GCF_001863855	non-GC1	2
GCF_001863865	non-GC1	2
GCF_001863875	non-GC1	2
GCF_001863905	non-GC1	2
GCF_001863935	non-GC1	2
GCF_001863955	non-GC1	2
GCF_001863965	non-GC1	2
GCF_001864015	non-GC1	2
GCF_001864035	non-GC1	2
GCF_001864045	non-GC1	2
GCF_001864065	non-GC1	2

GCF_001864095	non-GC1	46
GCF_001864115	non-GC1	2
GCF_001864125	non-GC1	2
GCF_001864155	non-GC1	46
GCF_001864165	non-GC1	46
GCF_001864195	non-GC1	2
GCF_001864235	non-GC1	46
GCF_001864285	non-GC1	2
GCF_001864305	non-GC1	2
GCF_001864315	non-GC1	2
GCF_001864355	non-GC1	46
GCF_001864375	non-GC1	2
GCF_001864385	non-GC1	2
GCF_001864395	non-GC1	2
GCF_001864435	non-GC1	46
GCF_001864455	non-GC1	2
GCF_001864465	non-GC1	46
GCF_001864475	non-GC1	2
GCF_001864505	non-GC1	2
GCF_001864555	non-GC1	23
GCF_001864565	non-GC1	2
GCF_001864575	non-GC1	879
GCF_001864615	non-GC1	2
GCF_001864635	non-GC1	2
GCF_001864655	non-GC1	2
GCF_001864675	non-GC1	2
GCF_001864715	non-GC1	10
GCF_001864755	non-GC1	2
GCF_001864795	non-GC1	2
GCF_001874685	non-GC1	79
GCF_001874765	non-GC1	85
GCF_001874885	non-GC1	85
GCF_001874915	non-GC1	2
GCF_001887305	non-GC1	2
GCF_001891205	non-GC1	920
GCF_001895125	non-GC1	412
GCF_001895985	non-GC1	2
GCF_001896005	non-GC1	79
GCF_001902375	non-GC1	2
GCF_001907125	non-GC1	2
GCF_001908295	non-GC1	10
GCF_001910585	non-GC1	1580
GCF_001910595	non-GC1	2
GCF_001910605	non-GC1	1580
GCF_001910615	non-GC1	1580
GCF_001910665	non-GC1	1580

GCF_001910675	non-GC1	2
GCF_001910685	non-GC1	1580
GCF_001922205	non-GC1	2
GCF_001922225	non-GC1	2
GCF_001922245	non-GC1	2
GCF_001922635	non-GC1	156
GCF_001922685	non-GC1	156
GCF_001922695	non-GC1	156
GCF_001922705	non-GC1	156
GCF_001922745	non-GC1	156
GCF_001922755	non-GC1	156
GCF_001923005	non-GC1	156
GCF_001939605	non-GC1	2
GCF_001939665	non-GC1	2
GCF_001941765	non-GC1	437
GCF_001949935	non-GC1	2
GCF_001949955	non-GC1	2
GCF_001949965	non-GC1	2
GCF_001950035	non-GC1	2
GCF_001950065	non-GC1	2
GCF_001950085	non-GC1	2
GCF_001950095	non-GC1	3
GCF_001950155	non-GC1	2
GCF_001950165	non-GC1	2
GCF_001950195	non-GC1	2
GCF_001950215	non-GC1	2
GCF_001950265	non-GC1	2
GCF_001950285	non-GC1	2
GCF_001950295	non-GC1	2
GCF_001950315	non-GC1	3
GCF_002006635	non-GC1	85
GCF_002006655	non-GC1	85
GCF_002006665	non-GC1	85
GCF_002014105	non-GC1	78
GCF_002014175	non-GC1	2
GCF_002014195	non-GC1	2
GCF_002014235	non-GC1	2
GCF_002014245	non-GC1	2
GCF_002014265	non-GC1	2
GCF_002014275	non-GC1	1088
GCF_002014315	non-GC1	1195
GCF_002014325	non-GC1	250
GCF_002014345	non-GC1	406
GCF_002014355	non-GC1	2
GCF_002014395	non-GC1	2
GCF_002014405	non-GC1	2

GCF_002014435	non-GC1	2
GCF_002014445	non-GC1	2
GCF_002014475	non-GC1	2
GCF_002016655	non-GC1	1088
GCF_002016735	non-GC1	2
GCF_002016745	non-GC1	2
GCF_002016775	non-GC1	2
GCF_002016785	non-GC1	79
GCF_002016815	non-GC1	2
GCF_002016825	non-GC1	250
GCF_002016855	non-GC1	406
GCF_002016865	non-GC1	2
GCF_002016915	non-GC1	2
GCF_002016935	non-GC1	2
GCF_002016955	non-GC1	2
GCF_002016975	non-GC1	1088
GCF_002016985	non-GC1	79
GCF_002016995	non-GC1	1088
GCF_002017035	non-GC1	2
GCF_002017055	non-GC1	2
GCF_002017065	non-GC1	2
GCF_002017095	non-GC1	2
GCF_002017105	non-GC1	2
GCF_002017115	non-GC1	2
GCF_002017125	non-GC1	2
GCF_002017175	non-GC1	1088
GCF_002017185	non-GC1	2
GCF_002017195	non-GC1	2
GCF_002017205	non-GC1	2
GCF_002017255	non-GC1	2
GCF_002017265	non-GC1	2
GCF_002017285	non-GC1	2
GCF_002017315	non-GC1	79
GCF_002017335	non-GC1	2
GCF_002017345	non-GC1	2
GCF_002017355	non-GC1	2
GCF_002017395	non-GC1	2
GCF_002017405	non-GC1	2
GCF_002017415	non-GC1	2
GCF_002017425	non-GC1	2
GCF_002017475	non-GC1	2
GCF_002017485	non-GC1	10
GCF_002017515	non-GC1	2
GCF_002017525	non-GC1	2
GCF_002017555	non-GC1	2
GCF_002017565	non-GC1	1088

GCF_002017595	non-GC1	1088
GCF_002017605	non-GC1	2
GCF_002017615	non-GC1	2
GCF_002017625	non-GC1	2
GCF_002017675	non-GC1	2
GCF_002017685	non-GC1	2
GCF_002017705	non-GC1	2
GCF_002017735	non-GC1	2
GCF_002017745	non-GC1	2
GCF_002017765	non-GC1	2
GCF_002017795	non-GC1	2
GCF_002017805	non-GC1	941
GCF_002018935	non-GC1	79
GCF_002018965	non-GC1	79
GCF_002018995	non-GC1	79
GCF_002019005	non-GC1	79
GCF_002019025	non-GC1	79
GCF_002019035	non-GC1	79
GCF_002028445	non-GC1	2
GCF_002028475	non-GC1	2
GCF_002028485	non-GC1	2
GCF_002028535	non-GC1	2
GCF_002082625	non-GC1	2
GCF_002082645	non-GC1	2
GCF_002082685	non-GC1	2
GCF_002082705	non-GC1	2
GCF_002082725	non-GC1	2
GCF_002082745	non-GC1	2
GCF_002082785	non-GC1	25
GCF_002082805	non-GC1	10
GCF_002082845	non-GC1	187
GCF_002082865	non-GC1	2
GCF_002082885	non-GC1	2
GCF_002085505	non-GC1	2
GCF_002116575	non-GC1	2
GCF_002116925	non-GC1	52
GCF_002119275	non-GC1	2
GCF_002119285	non-GC1	2
GCF_002119295	non-GC1	2
GCF_002136595	non-GC1	32
GCF_002136605	non-GC1	406
GCF_002136615	non-GC1	2
GCF_002136655	non-GC1	150
GCF_002136675	non-GC1	78
GCF_002136685	non-GC1	810
GCF_002136705	non-GC1	2

GCF_002136735	non-GC1	1048
GCF_002136745	non-GC1	2
GCF_002136775	non-GC1	2
GCF_002136815	non-GC1	142
GCF_002136825	non-GC1	2
GCF_002136855	non-GC1	78
GCF_002136885	non-GC1	2
GCF_002136895	non-GC1	48
GCF_002136935	non-GC1	2
GCF_002136955	non-GC1	494
GCF_002136965	non-GC1	2
GCF_002136995	non-GC1	464
GCF_002137035	non-GC1	2
GCF_002137045	non-GC1	2
GCF_002137185	non-GC1	78
GCF_002137215	non-GC1	49
GCF_002137225	non-GC1	2
GCF_002137255	non-GC1	848
GCF_002137265	non-GC1	498
GCF_002137285	non-GC1	2
GCF_002137295	non-GC1	2
GCF_002137335	non-GC1	406
GCF_002137345	non-GC1	2
GCF_002137395	non-GC1	2
GCF_002137425	non-GC1	2
GCF_002137445	non-GC1	2
GCF_002137475	non-GC1	2
GCF_002137495	non-GC1	79
GCF_002137525	non-GC1	2
GCF_002137555	non-GC1	2
GCF_002137565	non-GC1	2
GCF_002137575	non-GC1	203
GCF_002137645	non-GC1	49
GCF_002137695	non-GC1	2
GCF_002137715	non-GC1	2
GCF_002137725	non-GC1	126
GCF_002137755	non-GC1	10
GCF_002137775	non-GC1	48
GCF_002137795	non-GC1	2
GCF_002137815	non-GC1	2
GCF_002137875	non-GC1	2
GCF_002137895	non-GC1	2
GCF_002137945	non-GC1	203
GCF_002137955	non-GC1	150
GCF_002137995	non-GC1	2
GCF_002138005	non-GC1	2

GCF_002138035	non-GC1	2
GCF_002138055	non-GC1	32
GCF_002138065	non-GC1	78
GCF_002138105	non-GC1	25
GCF_002138115	non-GC1	2
GCF_002138155	non-GC1	49
GCF_002138165	non-GC1	2
GCF_002138175	non-GC1	669
GCF_002138215	non-GC1	2
GCF_002138245	non-GC1	2
GCF_002138295	non-GC1	2
GCF_002138305	non-GC1	2
GCF_002138315	non-GC1	2
GCF_002138345	non-GC1	2
GCF_002138375	non-GC1	2
GCF_002142735	non-GC1	1048
GCF_002142935	non-GC1	2
GCF_002142945	non-GC1	509
GCF_002142955	non-GC1	2
GCF_002142995	non-GC1	2
GCF_002143015	non-GC1	2
GCF_002143035	non-GC1	2
GCF_002143045	non-GC1	2
GCF_002143095	non-GC1	2
GCF_002143105	non-GC1	2
GCF_002143145	non-GC1	79
GCF_002143185	non-GC1	39
GCF_002143195	non-GC1	2
GCF_002143235	non-GC1	2
GCF_002143265	non-GC1	2
GCF_002143275	non-GC1	1088
GCF_002143315	non-GC1	2
GCF_002143335	non-GC1	2
GCF_002143345	non-GC1	941
GCF_002143375	non-GC1	406
GCF_002143395	non-GC1	2
GCF_002143405	non-GC1	2
GCF_002143425	non-GC1	2
GCF_002143475	non-GC1	150
GCF_002143495	non-GC1	2
GCF_002143555	non-GC1	2
GCF_002143575	non-GC1	2
GCF_002143605	non-GC1	32
GCF_002143625	non-GC1	2
GCF_002143655	non-GC1	2
GCF_002143675	non-GC1	2

GCF_002143685	non-GC1	2
GCF_002143715	non-GC1	2
GCF_002143725	non-GC1	48
GCF_002143755	non-GC1	406
GCF_002143775	non-GC1	2
GCF_002143785	non-GC1	2
GCF_002143795	non-GC1	2
GCF_002143835	non-GC1	2
GCF_002143855	non-GC1	10
GCF_002143875	non-GC1	32
GCF_002143885	non-GC1	973
GCF_002143935	non-GC1	2
GCF_002143955	non-GC1	216
GCF_002143975	non-GC1	25
GCF_002144685	non-GC1	2
GCF_002144695	non-GC1	250
GCF_002144725	non-GC1	2
GCF_002144755	non-GC1	2
GCF_002144765	non-GC1	250
GCF_002144795	non-GC1	412
GCF_002144805	non-GC1	150
GCF_002144835	non-GC1	49
GCF_002144925	non-GC1	79
GCF_002144955	non-GC1	32
GCF_002144965	non-GC1	10
GCF_002144975	non-GC1	2
GCF_002144995	non-GC1	221
GCF_002145035	non-GC1	2
GCF_002145055	non-GC1	2
GCF_002145065	non-GC1	2
GCF_002145095	non-GC1	250
GCF_002145115	non-GC1	2
GCF_002145125	non-GC1	1088
GCF_002145175	non-GC1	2
GCF_002145195	non-GC1	2
GCF_002145255	non-GC1	85
GCF_002145375	non-GC1	10
GCF_002145395	non-GC1	505
GCF_002145435	non-GC1	2
GCF_002148725	non-GC1	2
GCF_002148735	non-GC1	11
GCF_002148745	non-GC1	1165
GCF_002150405	non-GC1	3
GCF_002154155	non-GC1	187
GCF_002154215	non-GC1	2
GCF_002182345	non-GC1	79

GCF_002182365	non-GC1	2
GCF_002182385	non-GC1	2
GCF_002182405	non-GC1	10
GCF_002182425	non-GC1	2
GCF_002182445	non-GC1	2
GCF_002182465	non-GC1	221
GCF_002182485	non-GC1	1088
GCF_002182505	non-GC1	2
GCF_002182545	non-GC1	2
GCF_002182605	non-GC1	2
GCF_002182615	non-GC1	2
GCF_002182625	non-GC1	2
GCF_002182645	non-GC1	2
GCF_002182685	non-GC1	79
GCF_002182695	non-GC1	2
GCF_002182705	non-GC1	2
GCF_002182725	non-GC1	2
GCF_002182765	non-GC1	2
GCF_002182785	non-GC1	2
GCF_002182795	non-GC1	2
GCF_002182805	non-GC1	2
GCF_002182845	non-GC1	2
GCF_002182875	non-GC1	2
GCF_002182895	non-GC1	2
GCF_002182925	non-GC1	2
GCF_002182945	non-GC1	79
GCF_002182955	non-GC1	2
GCF_002182965	non-GC1	2
GCF_002183005	non-GC1	250
GCF_002183025	non-GC1	2
GCF_002183035	non-GC1	2
GCF_002183045	non-GC1	250
GCF_002183085	non-GC1	2
GCF_002183105	non-GC1	250
GCF_002183115	non-GC1	2
GCF_002183125	non-GC1	250
GCF_002183165	non-GC1	2
GCF_002183185	non-GC1	2
GCF_002183195	non-GC1	2
GCF_002183215	non-GC1	2
GCF_002183245	non-GC1	2
GCF_002183265	non-GC1	2
GCF_002183275	non-GC1	2
GCF_002183285	non-GC1	2
GCF_002183325	non-GC1	2
GCF_002183345	non-GC1	2

GCF_002183355	non-GC1	2
GCF_002183365	non-GC1	2
GCF_002183405	non-GC1	2
GCF_002183425	non-GC1	250
GCF_002183435	non-GC1	2
GCF_002183445	non-GC1	2
GCF_002183485	non-GC1	2
GCF_002183505	non-GC1	79
GCF_002183515	non-GC1	3
GCF_002183545	non-GC1	2
GCF_002183555	non-GC1	2
GCF_002183585	non-GC1	2
GCF_002183595	non-GC1	2
GCF_002183625	non-GC1	2
GCF_002183635	non-GC1	2
GCF_002183665	non-GC1	2
GCF_002183675	non-GC1	48
GCF_002183705	non-GC1	2
GCF_002183725	non-GC1	2
GCF_002183735	non-GC1	2
GCF_002183765	non-GC1	2
GCF_002183785	non-GC1	2
GCF_002183805	non-GC1	2
GCF_002183815	non-GC1	2
GCF_002183845	non-GC1	2
GCF_002183855	non-GC1	2
GCF_002183875	non-GC1	2
GCF_002183905	non-GC1	2
GCF_002183925	non-GC1	10
GCF_002183945	non-GC1	2
GCF_002183955	non-GC1	2
GCF_002183975	non-GC1	2
GCF_002184005	non-GC1	2
GCF_002184025	non-GC1	2
GCF_002184035	non-GC1	2
GCF_002184055	non-GC1	2
GCF_002184085	non-GC1	2
GCF_002184105	non-GC1	2
GCF_002184265	non-GC1	2
GCF_002184275	non-GC1	79
GCF_002184285	non-GC1	2
GCF_002184325	non-GC1	406
GCF_002184335	non-GC1	406
GCF_002184365	non-GC1	79
GCF_002184385	non-GC1	2
GCF_002184565	non-GC1	2

GCF_002184605	non-GC1	2
GCF_002197745	non-GC1	622
GCF_002212115	non-GC1	2
GCF_002212155	non-GC1	2
GCF_002212165	non-GC1	2
GCF_002212225	non-GC1	113
GCF_002212235	non-GC1	2
GCF_002212245	non-GC1	2
GCF_002212285	non-GC1	2
GCF_002212305	non-GC1	2
GCF_002212315	non-GC1	2
GCF_002212365	non-GC1	2
GCF_002212385	non-GC1	2
GCF_002212405	non-GC1	2
GCF_002212445	non-GC1	2
GCF_002212465	non-GC1	2
GCF_002212485	non-GC1	2
GCF_002212525	non-GC1	2
GCF_002212535	non-GC1	2
GCF_002212565	non-GC1	2
GCF_002212605	non-GC1	2
GCF_002212625	non-GC1	2
GCF_002212635	non-GC1	2
GCF_002212685	non-GC1	2
GCF_002212705	non-GC1	2
GCF_002212715	non-GC1	2
GCF_002239725	non-GC1	16
GCF_002239735	non-GC1	2
GCF_002239865	non-GC1	2
GCF_002239885	non-GC1	2
GCF_002241105	non-GC1	2
GCF_002249135	non-GC1	2
GCF_002249185	non-GC1	2
GCF_002249225	non-GC1	2
GCF_002249245	non-GC1	2
GCF_002249255	non-GC1	2
GCF_002249265	non-GC1	2
GCF_002249305	non-GC1	2
GCF_002249325	non-GC1	2
GCF_002249335	non-GC1	2
GCF_002249365	non-GC1	215
GCF_002249385	non-GC1	2
GCF_002249405	non-GC1	2
GCF_002249425	non-GC1	2
GCF_002249435	non-GC1	215
GCF_002249465	non-GC1	2

GCF_002249475	non-GC1	571
GCF_002249505	non-GC1	2
GCF_002249525	non-GC1	2
GCF_002249545	non-GC1	2
GCF_002249585	non-GC1	2
GCF_002249605	non-GC1	2
GCF_002249655	non-GC1	2
GCF_002250465	non-GC1	730
GCF_002261505	non-GC1	2
GCF_002261535	non-GC1	307
GCF_002265555	non-GC1	49
GCF_002265675	non-GC1	2
GCF_002277145	non-GC1	2
GCF_002277175	non-GC1	2
GCF_002277185	non-GC1	2
GCF_002277215	non-GC1	2
GCF_002277225	non-GC1	2
GCF_002277235	non-GC1	2
GCF_002277275	non-GC1	2
GCF_002277285	non-GC1	2
GCF_002277315	non-GC1	2
GCF_002277325	non-GC1	2
GCF_002277355	non-GC1	2
GCF_002277365	non-GC1	2
GCF_002277385	non-GC1	2
GCF_002277405	non-GC1	2
GCF_002277435	non-GC1	2
GCF_002277445	non-GC1	2
**GCF_002277485**	**non-GC1**	**2**
GCF_002277505	non-GC1	2
GCF_002277515	non-GC1	2
GCF_002277535	non-GC1	2
GCF_002277585	non-GC1	2
GCF_002277605	non-GC1	2
GCF_002277635	non-GC1	2
GCF_002277655	non-GC1	2
GCF_002277675	non-GC1	2
GCF_002277705	non-GC1	2
GCF_002367895	non-GC1	294
GCF_002411775	non-GC1	164
GCF_002416325	non-GC1	3
GCF_002416365	non-GC1	3
GCF_002416395	non-GC1	2
GCF_002416425	non-GC1	2
GCF_002416435	non-GC1	2
GCF_002504145	non-GC1	138

GCF_002573725	non-GC1	923
GCF_002573735	non-GC1	927
GCF_002573805	non-GC1	25
GCF_002573825	non-GC1	240
GCF_002573875	non-GC1	1179
GCF_002573895	non-GC1	690
GCF_002573915	non-GC1	138
GCF_002753915	non-GC1	2
GCF_002760785	non-GC1	85
GCF_002760835	non-GC1	85
GCF_002761575	non-GC1	2
GCF_002762095	non-GC1	2
GCF_002762115	non-GC1	2
GCF_002762155	non-GC1	2
GCF_002762495	non-GC1	2
GCF_002762505	non-GC1	2
GCF_002762535	non-GC1	2
GCF_002837005	non-GC1	2
GCF_002837035	non-GC1	2
GCF_002837885	non-GC1	575
GCF_002837895	non-GC1	513
GCF_002843665	non-GC1	2
GCF_002885855	non-GC1	286
GCF_002902885	non-GC1	1543
GCF_002922935	non-GC1	107
GCF_002927805	non-GC1	10
GCF_002927855	non-GC1	25
GCF_002927915	non-GC1	156
GCF_002927965	non-GC1	25
GCF_002927995	non-GC1	10
GCF_002928015	non-GC1	106
GCF_002928075	non-GC1	108
GCF_002928095	non-GC1	156
GCF_002928145	non-GC1	77
GCF_002928155	non-GC1	267
GCF_002928195	non-GC1	106
GCF_002947415	non-GC1	2
GCF_002947845	non-GC1	79
GCF_002948925	non-GC1	229
GCF_002950415	non-GC1	2
GCF_002950425	non-GC1	2
GCF_002950435	non-GC1	46
GCF_002950455	non-GC1	2
GCF_002950495	non-GC1	2
GCF_002950525	non-GC1	2
GCF_002950545	non-GC1	2

GCF_002950595	non-GC1	2
GCF_002950615	non-GC1	2
GCF_002950625	non-GC1	2
GCF_002950655	non-GC1	2
GCF_002950675	non-GC1	2
GCF_002950715	non-GC1	2
GCF_002950725	non-GC1	2
GCF_002950755	non-GC1	2
GCF_002950795	non-GC1	2
GCF_002950815	non-GC1	2
GCF_002950825	non-GC1	2
GCF_002950855	non-GC1	2
GCF_002950895	non-GC1	2
GCF_002950915	non-GC1	2
GCF_002950925	non-GC1	2
GCF_002950975	non-GC1	2
GCF_002950995	non-GC1	2
GCF_002951055	non-GC1	2
GCF_002951075	non-GC1	2
GCF_002951095	non-GC1	2
GCF_002951135	non-GC1	2
GCF_002951155	non-GC1	2
GCF_002951175	non-GC1	2
GCF_002951215	non-GC1	2
GCF_002951235	non-GC1	2
GCF_002951255	non-GC1	2
GCF_002951275	non-GC1	2
GCF_002951315	non-GC1	2
GCF_002951335	non-GC1	2
GCF_002951345	non-GC1	2
GCF_002951375	non-GC1	2
GCF_002951415	non-GC1	2
GCF_002951435	non-GC1	2
GCF_002992205	non-GC1	2
GCF_002992225	non-GC1	2
GCF_002992235	non-GC1	2
GCF_002992255	non-GC1	2
GCF_002992285	non-GC1	2
GCF_002992305	non-GC1	2
GCF_002992325	non-GC1	2
GCF_002992345	non-GC1	2
GCF_002992365	non-GC1	2
GCF_002992385	non-GC1	2
GCF_002992405	non-GC1	2
GCF_002992425	non-GC1	2
GCF_002992445	non-GC1	2

GCF_002992465	non-GC1	2
GCF_002992485	non-GC1	2
GCF_002992505	non-GC1	2
GCF_002992525	non-GC1	2
GCF_002992545	non-GC1	2
GCF_002992565	non-GC1	113
GCF_002992585	non-GC1	2
GCF_002992605	non-GC1	2
GCF_002992625	non-GC1	2
GCF_002992645	non-GC1	113
GCF_002992665	non-GC1	2
GCF_002992685	non-GC1	2
GCF_002992705	non-GC1	2
GCF_002992715	non-GC1	2
GCF_002992745	non-GC1	2
GCF_002992765	non-GC1	2
GCF_002992775	non-GC1	2
GCF_002992805	non-GC1	2
GCF_002992825	non-GC1	2
GCF_002992845	non-GC1	2
GCF_002992865	non-GC1	2
GCF_002992875	non-GC1	2
GCF_002992905	non-GC1	2
GCF_003006035	non-GC1	25
GCF_003006835	non-GC1	2
GCF_003006845	non-GC1	2
GCF_003006875	non-GC1	2
GCF_003006885	non-GC1	2
GCF_003006945	non-GC1	52
GCF_003006975	non-GC1	2
GCF_003007015	non-GC1	2
GCF_003007025	non-GC1	2
GCF_003007055	non-GC1	2
GCF_003007065	non-GC1	2
GCF_003007095	non-GC1	2
GCF_003007115	non-GC1	2
GCF_003007135	non-GC1	2
GCF_003007145	non-GC1	2
GCF_003007215	non-GC1	2
GCF_003007245	non-GC1	626
GCF_003007275	non-GC1	2
GCF_003007335	non-GC1	2
GCF_003007355	non-GC1	2
GCF_003007395	non-GC1	2
GCF_003007405	non-GC1	2
GCF_003007435	non-GC1	2

GCF_003007455	non-GC1	2
GCF_003007475	non-GC1	2
GCF_003007485	non-GC1	2
GCF_003007515	non-GC1	2
GCF_003007535	non-GC1	2
GCF_003007585	non-GC1	2
GCF_003007615	non-GC1	2
GCF_003007635	non-GC1	2
GCF_003007655	non-GC1	2
GCF_003010655	non-GC1	2
GCF_003012695	non-GC1	636
GCF_003020265	non-GC1	2
GCF_003020275	non-GC1	2
GCF_003020285	non-GC1	2
GCF_003020325	non-GC1	2
GCF_003020385	non-GC1	2
GCF_003020445	non-GC1	2
GCF_003020455	non-GC1	2
GCF_003020485	non-GC1	2
GCF_003020505	non-GC1	2
GCF_003020525	non-GC1	2
GCF_003020545	non-GC1	2
GCF_003020565	non-GC1	2
GCF_003020575	non-GC1	2
GCF_003029495	non-GC1	2
GCF_003038935	non-GC1	636
GCF_003038965	non-GC1	636
GCF_003039005	non-GC1	1580
GCF_003039035	non-GC1	2
GCF_003039055	non-GC1	636
GCF_003039075	non-GC1	388
GCF_003039115	non-GC1	636
GCF_003039135	non-GC1	636
GCF_003039155	non-GC1	636
GCF_003039175	non-GC1	636
GCF_003039195	non-GC1	636
GCF_003039215	non-GC1	636
GCF_003039255	non-GC1	636
GCF_003039275	non-GC1	1580
GCF_003039295	non-GC1	636
GCF_003039315	non-GC1	636
GCF_003039335	non-GC1	636
GCF_003039345	non-GC1	636
GCF_003039375	non-GC1	2
GCF_003039395	non-GC1	636
GCF_003039415	non-GC1	636

GCF_003039475	non-GC1	2
GCF_003056465	non-GC1	2
GCF_003056475	non-GC1	2
GCF_003056505	non-GC1	2
GCF_003056515	non-GC1	2
GCF_003056525	non-GC1	2
GCF_003070915	non-GC1	150
GCF_003070935	non-GC1	575
GCF_003070945	non-GC1	216
GCF_003072095	non-GC1	2
GCF_003130565	non-GC1	15
GCF_003130905	non-GC1	193
GCF_003130915	non-GC1	329
GCF_003130925	non-GC1	40
GCF_003130945	non-GC1	193
GCF_003130985	non-GC1	36
GCF_003131005	non-GC1	106
GCF_003131025	non-GC1	10
GCF_003181015	non-GC1	2
GCF_003183965	non-GC1	2
GCF_003194535	non-GC1	2
GCF_003231175	non-GC1	52
GCF_003231185	non-GC1	341
GCF_003240485	non-GC1	85
GCF_003264255	non-GC1	2
GCF_003264275	non-GC1	2
GCF_003264295	non-GC1	2
GCF_003288775	non-GC1	2
GCF_003335945	non-GC1	2
GCF_003335955	non-GC1	2
GCF_003335965	non-GC1	255
GCF_003336005	non-GC1	2
GCF_003336045	non-GC1	2
GCF_003336075	non-GC1	2
GCF_003336125	non-GC1	2
GCF_003345235	non-GC1	156
GCF_003345795	non-GC1	2
GCF_003345805	non-GC1	2
GCF_003345835	non-GC1	2
GCF_003345855	non-GC1	2
GCF_003345865	non-GC1	158
GCF_003345915	non-GC1	2
GCF_003345925	non-GC1	158
GCF_003345955	non-GC1	2
GCF_003345995	non-GC1	2
GCF_003347295	non-GC1	636

GCF_003347305	non-GC1	2
GCF_003347325	non-GC1	2
GCF_003347355	non-GC1	1580
GCF_003347375	non-GC1	1580
GCF_003347385	non-GC1	2
GCF_003347395	non-GC1	2
GCF_003347435	non-GC1	636
GCF_003352485	non-GC1	2
GCF_003352495	non-GC1	2
GCF_003352525	non-GC1	2
GCF_003352535	non-GC1	2
GCF_003352595	non-GC1	2
GCF_003352605	non-GC1	2
GCF_003352615	non-GC1	2
GCF_003352665	non-GC1	2
GCF_003352675	non-GC1	2
GCF_003352685	non-GC1	2
GCF_003352725	non-GC1	2
GCF_003355585	non-GC1	2
GCF_003355595	non-GC1	2
GCF_003355605	non-GC1	2
GCF_003355665	non-GC1	2
GCF_003355675	non-GC1	2
GCF_003355705	non-GC1	2
GCF_003355725	non-GC1	2
GCF_003355745	non-GC1	2
GCF_003355755	non-GC1	2
GCF_003355785	non-GC1	2
GCF_003355795	non-GC1	2
GCF_003355815	non-GC1	2
GCF_003355845	non-GC1	2
GCF_003355865	non-GC1	2
GCF_003355875	non-GC1	2
GCF_003355885	non-GC1	2
GCF_003355915	non-GC1	2
GCF_003355945	non-GC1	2
GCF_003355965	non-GC1	2
GCF_003355985	non-GC1	2
GCF_003356025	non-GC1	33
GCF_003356045	non-GC1	2
GCF_003356055	non-GC1	2
GCF_003356085	non-GC1	2
GCF_003356105	non-GC1	2
GCF_003356125	non-GC1	2
GCF_003356145	non-GC1	2
GCF_003356185	non-GC1	2

GCF_003356205	non-GC1	2
GCF_003356225	non-GC1	2
GCF_003356235	non-GC1	2
GCF_003356265	non-GC1	2
GCF_003356285	non-GC1	2
GCF_003356295	non-GC1	2
GCF_003356315	non-GC1	2
GCF_003356345	non-GC1	2
GCF_003356365	non-GC1	2
GCF_003356385	non-GC1	2
GCF_003356425	non-GC1	2
GCF_003356445	non-GC1	2
GCF_003356455	non-GC1	2
GCF_003356485	non-GC1	2
GCF_003356525	non-GC1	2
GCF_003356535	non-GC1	2
GCF_003356565	non-GC1	2
GCF_003356575	non-GC1	2
GCF_003356605	non-GC1	2
GCF_003356625	non-GC1	2
GCF_003356635	non-GC1	2
GCF_003356645	non-GC1	2
GCF_003356685	non-GC1	2
GCF_003356705	non-GC1	2
GCF_003356725	non-GC1	2
GCF_003356745	non-GC1	2
GCF_003356765	non-GC1	2
GCF_003356785	non-GC1	2
GCF_003356795	non-GC1	2
GCF_003356815	non-GC1	2
GCF_003356845	non-GC1	2
GCF_003356865	non-GC1	2
GCF_003356875	non-GC1	2
GCF_003356905	non-GC1	2
GCF_003356925	non-GC1	2
GCF_003356945	non-GC1	2
GCF_003356965	non-GC1	2
GCF_003356975	non-GC1	2
GCF_003357005	non-GC1	2
GCF_003357025	non-GC1	2
GCF_003357035	non-GC1	2
GCF_003357045	non-GC1	2
GCF_003357085	non-GC1	2
GCF_003382235	non-GC1	164
GCF_003431385	non-GC1	25
GCF_003431865	non-GC1	32

GCF_003516005	non-GC1	1544
GCF_003522665	non-GC1	422
GCF_003522705	non-GC1	10
GCF_003522785	non-GC1	2
GCF_003522885	non-GC1	2
GCF_003547115	non-GC1	32
GCF_003569425	non-GC1	239
GCF_003569445	non-GC1	25
GCF_003569625	non-GC1	138
GCF_003569645	non-GC1	911
GCF_003574715	non-GC1	1158
GCF_003583525	non-GC1	991
GCF_003583535	non-GC1	25
GCF_003583565	non-GC1	25
GCF_003583575	non-GC1	25
GCF_003583605	non-GC1	25
GCF_003583615	non-GC1	991
GCF_003583625	non-GC1	25
GCF_003583665	non-GC1	15
GCF_003584255	non-GC1	25
GCF_003584275	non-GC1	25
GCF_003584305	non-GC1	25
GCF_003584315	non-GC1	25
GCF_003584325	non-GC1	25
GCF_003584365	non-GC1	25
GCF_003584375	non-GC1	25
GCF_003584385	non-GC1	25
GCF_003584425	non-GC1	25
GCF_003584445	non-GC1	25
GCF_003584455	non-GC1	25
GCF_003584485	non-GC1	25
GCF_003584505	non-GC1	991
GCF_003584515	non-GC1	25
GCF_003584535	non-GC1	991
GCF_003591615	non-GC1	922
GCF_003595665	non-GC1	25
GCF_003595675	non-GC1	25
GCF_003595685	non-GC1	25
GCF_003595745	non-GC1	991
GCF_003595755	non-GC1	991
GCF_003595765	non-GC1	25
GCF_003595805	non-GC1	991
GCF_003595815	non-GC1	25
GCF_003595835	non-GC1	991
GCF_003595865	non-GC1	25
GCF_003595875	non-GC1	79

GCF_003595885	non-GC1	991
GCF_003595925	non-GC1	25
GCF_003595935	non-GC1	25
GCF_003595955	non-GC1	25
GCF_003595985	non-GC1	79
GCF_003595995	non-GC1	991
GCF_003596015	non-GC1	991
GCF_003596025	non-GC1	991
GCF_003596065	non-GC1	991
GCF_003596075	non-GC1	991
GCF_003596105	non-GC1	991
GCF_003596125	non-GC1	25
GCF_003596135	non-GC1	25
GCF_003596165	non-GC1	267
GCF_003596175	non-GC1	25
GCF_003596205	non-GC1	25
GCF_003596215	non-GC1	79
GCF_003596235	non-GC1	25
GCF_003596265	non-GC1	991
GCF_003596275	non-GC1	991
GCF_003597385	non-GC1	991
GCF_003627455	non-GC1	2
GCF_003697885	non-GC1	108
GCF_003711855	non-GC1	2
GCF_003711895	non-GC1	2
GCF_003721135	non-GC1	40
GCF_003812065	non-GC1	1542
GCF_003812385	non-GC1	57
GCF_003812485	non-GC1	79
GCF_003843745	non-GC1	2
GCF_003863935	non-GC1	2
GCF_003931755	non-GC1	2
GCF_003931775	non-GC1	2
GCF_003939385	non-GC1	2
GCF_003939395	non-GC1	2
GCF_003939415	non-GC1	187
GCF_003939465	non-GC1	2
GCF_003939505	non-GC1	2
GCF_003939525	non-GC1	2
GCF_003939585	non-GC1	145
GCF_003939595	non-GC1	145
GCF_003939625	non-GC1	2
GCF_003939645	non-GC1	2
GCF_003939655	non-GC1	2
GCF_003939925	non-GC1	2
GCF_003939985	non-GC1	2

GCF_003940005	non-GC1	187
GCF_003940065	non-GC1	2
GCF_003940105	non-GC1	52
GCF_003940125	non-GC1	2
GCF_003940135	non-GC1	2
GCF_003940325	non-GC1	2
GCF_003940335	non-GC1	2
GCF_003940365	non-GC1	2
GCF_003940395	non-GC1	2
GCF_003940865	non-GC1	2
GCF_003940875	non-GC1	2
GCF_003940895	non-GC1	821
GCF_003940915	non-GC1	2
GCF_003940925	non-GC1	2
GCF_003940965	non-GC1	2
GCF_003941015	non-GC1	2
GCF_003941025	non-GC1	2
GCF_003941065	non-GC1	2
GCF_003941085	non-GC1	2
GCF_003941095	non-GC1	821
GCF_003941125	non-GC1	2
GCF_003941135	non-GC1	2
GCF_003941145	non-GC1	2
GCF_003941185	non-GC1	2
GCF_003941195	non-GC1	2
GCF_003941205	non-GC1	2
GCF_003941245	non-GC1	2
GCF_003941255	non-GC1	2
GCF_003941285	non-GC1	2
GCF_003941295	non-GC1	52
GCF_003941305	non-GC1	2
GCF_003941315	non-GC1	2
GCF_003941325	non-GC1	2
GCF_003941385	non-GC1	2
GCF_003941405	non-GC1	2
GCF_003941415	non-GC1	2
GCF_003941425	non-GC1	2
GCF_003941435	non-GC1	2
GCF_003941485	non-GC1	2
GCF_003941505	non-GC1	2
GCF_003941515	non-GC1	2
GCF_003941525	non-GC1	2
GCF_003941545	non-GC1	2
GCF_003941585	non-GC1	2
GCF_003941605	non-GC1	2
GCF_003941615	non-GC1	2

GCF_003941625	non-GC1	2
GCF_003941645	non-GC1	2
GCF_003941685	non-GC1	2
GCF_003941695	non-GC1	2
GCF_003941715	non-GC1	2
GCF_003941735	non-GC1	2
GCF_003941755	non-GC1	2
GCF_003941785	non-GC1	2
GCF_003941795	non-GC1	2
GCF_003941815	non-GC1	2
GCF_003947815	non-GC1	2
GCF_003947855	non-GC1	2
GCF_003947865	non-GC1	2
GCF_003948085	non-GC1	78
GCF_003948095	non-GC1	2
GCF_003948125	non-GC1	78
GCF_003948145	non-GC1	2
GCF_003948185	non-GC1	2
GCF_003948195	non-GC1	3
GCF_003948225	non-GC1	2
GCF_003948235	non-GC1	429
GCF_003948295	non-GC1	6
GCF_003948305	non-GC1	78
**GCF_003948345**	**non-GC1**	**416**
GCF_003948375	non-GC1	78
GCF_003948385	non-GC1	411
GCF_003948415	non-GC1	412
GCF_003948425	non-GC1	2
GCF_003948455	non-GC1	2
GCF_003948465	non-GC1	2
GCF_003948495	non-GC1	1436
GCF_003948505	non-GC1	2
GCF_003948535	non-GC1	2
GCF_003948545	non-GC1	417
GCF_003948585	non-GC1	2
GCF_003948595	non-GC1	406
GCF_003948605	non-GC1	2
GCF_003948645	non-GC1	2
GCF_003948675	non-GC1	241
GCF_003948685	non-GC1	437
GCF_003948725	non-GC1	498
GCF_003948745	non-GC1	2
GCF_003948775	non-GC1	1438
GCF_003948795	non-GC1	2
GCF_003948805	non-GC1	2
GCF_003948835	non-GC1	1437

GCF_003948875	non-GC1	2
GCF_003948885	non-GC1	2
GCF_003948905	non-GC1	32
GCF_003948915	non-GC1	415
GCF_003948975	non-GC1	2
GCF_003948985	non-GC1	108
GCF_003949015	non-GC1	79
GCF_003949025	non-GC1	2
GCF_003949075	non-GC1	2
GCF_003949095	non-GC1	78
GCF_003949105	non-GC1	78
GCF_003949115	non-GC1	78
GCF_003949125	non-GC1	203
GCF_003949175	non-GC1	2
GCF_003949195	non-GC1	416
GCF_003949205	non-GC1	2
GCF_003949225	non-GC1	2
GCF_003949235	non-GC1	152
GCF_003949275	non-GC1	2
GCF_003949325	non-GC1	2
GCF_003949335	non-GC1	33
GCF_003949365	non-GC1	2
GCF_003949395	non-GC1	78
GCF_003949405	non-GC1	78
GCF_003949425	non-GC1	2
GCF_003949435	non-GC1	78
GCF_003949485	non-GC1	2
GCF_003949555	non-GC1	2
GCF_003949575	non-GC1	415
GCF_003949595	non-GC1	2
GCF_003949615	non-GC1	2
GCF_003949655	non-GC1	2
GCF_003949725	non-GC1	78
GCF_003949755	non-GC1	2
GCF_003949765	non-GC1	2
GCF_003949935	non-GC1	2
GCF_003953975	non-GC1	2
GCF_003953985	non-GC1	2
GCF_003954005	non-GC1	2
GCF_003954085	non-GC1	2
GCF_003954095	non-GC1	2
GCF_003954105	non-GC1	2
GCF_003954115	non-GC1	2
GCF_003954175	non-GC1	2
GCF_003954215	non-GC1	2
GCF_003954235	non-GC1	2

GCF_003954275	non-GC1	2
GCF_003954315	non-GC1	2
GCF_003954365	non-GC1	2
GCF_003954415	non-GC1	2
GCF_003954595	non-GC1	2
GCF_003954795	non-GC1	2
GCF_003954845	non-GC1	2
GCF_003954905	non-GC1	2
GCF_003955235	non-GC1	2
GCF_003955305	non-GC1	2
GCF_003955355	non-GC1	25
GCF_003955385	non-GC1	2
GCF_003955435	non-GC1	2
GCF_003955485	non-GC1	2
GCF_003965825	non-GC1	2
GCF_003966015	non-GC1	136
GCF_003970215	non-GC1	136
GCF_003976955	non-GC1	156
GCF_003976985	non-GC1	156
GCF_003977015	non-GC1	156
GCF_003977045	non-GC1	156
GCF_003979005	non-GC1	2
GCF_003984795	non-GC1	2
GCF_003991295	non-GC1	570
GCF_003994455	non-GC1	1303
GCF_004028375	non-GC1	77
GCF_004101685	non-GC1	2
GCF_004101705	non-GC1	2
GCF_004101725	non-GC1	2
GCF_004101745	non-GC1	2
GCF_004101965	non-GC1	2
GCF_004123315	non-GC1	85
GCF_004136355	non-GC1	2
GCF_004136905	non-GC1	2
GCF_004136925	non-GC1	2
GCF_004152685	non-GC1	241
GCF_004152945	non-GC1	2
GCF_004153595	non-GC1	25
GCF_004168615	non-GC1	2
GCF_004194555	non-GC1	1197
GCF_004208995	non-GC1	156
GCF_004209005	non-GC1	156
GCF_004209025	non-GC1	156
GCF_004209035	non-GC1	156
GCF_004209075	non-GC1	156
GCF_004209195	non-GC1	25

GCF_004209205	non-GC1	156
GCF_004209215	non-GC1	156
GCF_004209225	non-GC1	156
GCF_004209675	non-GC1	2
GCF_004209715	non-GC1	2
GCF_004282835	non-GC1	78
GCF_004282845	non-GC1	78
GCF_004296275	non-GC1	2
GCF_004299615	non-GC1	156
GCF_004321575	non-GC1	156
GCF_004321595	non-GC1	156
GCF_004347325	non-GC1	79
GCF_004357075	non-GC1	2
GCF_004357095	non-GC1	2
GCF_004357175	non-GC1	2
GCF_004357205	non-GC1	25
GCF_004357225	non-GC1	23
GCF_004357275	non-GC1	2
GCF_004357285	non-GC1	158
GCF_004359905	non-GC1	79
GCF_004377785	non-GC1	2
GCF_004378285	non-GC1	2
GCF_004378295	non-GC1	2
GCF_004378395	non-GC1	2
GCF_004378435	non-GC1	2
GCF_004378505	non-GC1	2
GCF_004378525	non-GC1	2
GCF_004378535	non-GC1	2
GCF_004378545	non-GC1	2
GCF_004378625	non-GC1	2
GCF_004564095	non-GC1	345
GCF_004564115	non-GC1	345
GCF_004758865	non-GC1	10
GCF_004768705	non-GC1	85
GCF_004790725	non-GC1	164
GCF_004794205	non-GC1	156
GCF_004797155	non-GC1	437
GCF_004799205	non-GC1	2
GCF_004799225	non-GC1	23
GCF_004912235	non-GC1	25
GCF_005048905	non-GC1	2
GCF_005280375	non-GC1	2
GCF_005280435	non-GC1	622
GCF_005280675	non-GC1	285
GCF_005280695	non-GC1	2
GCF_005280715	non-GC1	2

GCF_005298065	non-GC1	388
GCF_005298085	non-GC1	388
GCF_005518095	non-GC1	25
GCF_005519135	non-GC1	2
GCF_005671375	non-GC1	2
GCF_005784435	non-GC1	2
GCF_005784445	non-GC1	2
GCF_005784525	non-GC1	2
GCF_005784535	non-GC1	2
GCF_005784565	non-GC1	2
GCF_005784575	non-GC1	2
GCF_005784585	non-GC1	2
GCF_005784625	non-GC1	2
GCF_005784645	non-GC1	2
GCF_005784655	non-GC1	2
GCF_005784685	non-GC1	2
GCF_005784705	non-GC1	2
GCF_005784725	non-GC1	2
GCF_005784745	non-GC1	2
GCF_005784755	non-GC1	2
GCF_005784785	non-GC1	2
GCF_005784795	non-GC1	2
GCF_005784825	non-GC1	2
GCF_005784835	non-GC1	2
GCF_005784865	non-GC1	2
GCF_005784875	non-GC1	2
GCF_005784885	non-GC1	2
GCF_005784925	non-GC1	2
GCF_005784945	non-GC1	2
GCF_005784965	non-GC1	2
GCF_005784975	non-GC1	2
GCF_005784985	non-GC1	2
GCF_005785025	non-GC1	2
GCF_005785045	non-GC1	2
GCF_005785065	non-GC1	2
GCF_005785075	non-GC1	2
GCF_005785095	non-GC1	2
GCF_005785115	non-GC1	2
GCF_005785125	non-GC1	2
GCF_005785165	non-GC1	2
GCF_005785185	non-GC1	2
GCF_005785205	non-GC1	2
GCF_005785215	non-GC1	2
GCF_005785225	non-GC1	52
GCF_005785265	non-GC1	52
GCF_005785275	non-GC1	52

GCF_005785305	non-GC1	2
GCF_005785315	non-GC1	2
GCF_005785325	non-GC1	2
GCF_005785355	non-GC1	2
GCF_005785385	non-GC1	2
GCF_005785405	non-GC1	2
GCF_005785415	non-GC1	2
GCF_005785445	non-GC1	2
GCF_005785455	non-GC1	2
GCF_005785465	non-GC1	2
GCF_005785505	non-GC1	2
GCF_005785515	non-GC1	2
GCF_005785545	non-GC1	2
GCF_005785555	non-GC1	2
GCF_005785575	non-GC1	2
GCF_005785605	non-GC1	2
GCF_005785615	non-GC1	2
GCF_005785645	non-GC1	2
GCF_005785675	non-GC1	2
GCF_005785695	non-GC1	2
GCF_005785725	non-GC1	2
GCF_005785745	non-GC1	2
GCF_005785765	non-GC1	2
GCF_005785775	non-GC1	2
GCF_005785785	non-GC1	2
GCF_005785815	non-GC1	2
GCF_005785845	non-GC1	2
GCF_005785865	non-GC1	2
GCF_005785875	non-GC1	2
GCF_005785885	non-GC1	2
GCF_005785895	non-GC1	2
GCF_005785945	non-GC1	2
GCF_005785965	non-GC1	2
GCF_005785985	non-GC1	2
GCF_005785995	non-GC1	2
GCF_005786005	non-GC1	2
GCF_005786015	non-GC1	2
GCF_005786065	non-GC1	2
GCF_005786085	non-GC1	2
GCF_005786095	non-GC1	2
GCF_005786105	non-GC1	2
GCF_005786115	non-GC1	2
GCF_005786135	non-GC1	2
GCF_005786185	non-GC1	2
GCF_005786205	non-GC1	2
GCF_005786215	non-GC1	2

GCF_005786225	non-GC1	2
GCF_005786235	non-GC1	2
GCF_005786285	non-GC1	2
GCF_005786305	non-GC1	2
GCF_005786315	non-GC1	2
GCF_005786335	non-GC1	2
GCF_005786365	non-GC1	2
GCF_005786375	non-GC1	2
GCF_005786405	non-GC1	2
GCF_005786425	non-GC1	2
GCF_005786435	non-GC1	2
GCF_005786445	non-GC1	2
GCF_005786455	non-GC1	2
GCF_005786505	non-GC1	2
GCF_005786525	non-GC1	2
GCF_005786535	non-GC1	2
GCF_005786545	non-GC1	2
GCF_005786585	non-GC1	2
GCF_005786595	non-GC1	2
GCF_005819005	non-GC1	2
GCF_005819035	non-GC1	2
GCF_005819065	non-GC1	2
GCF_005819085	non-GC1	2
GCF_005819095	non-GC1	2
GCF_005819115	non-GC1	2
GCF_005819135	non-GC1	2
GCF_005819165	non-GC1	2
GCF_005819175	non-GC1	2
GCF_005819205	non-GC1	2
GCF_005819225	non-GC1	2
GCF_005819265	non-GC1	2
GCF_005819275	non-GC1	400
GCF_005819305	non-GC1	2
GCF_005819315	non-GC1	113
GCF_005819325	non-GC1	2
GCF_005819365	non-GC1	113
GCF_005819375	non-GC1	2
GCF_005819405	non-GC1	2
GCF_005819415	non-GC1	2
GCF_005819425	non-GC1	2
GCF_005819445	non-GC1	2
GCF_005819475	non-GC1	113
GCF_005819505	non-GC1	636
GCF_005819515	non-GC1	2
GCF_005819535	non-GC1	2
GCF_005819545	non-GC1	2

GCF_005819585	non-GC1	2
GCF_005819595	non-GC1	2
GCF_005819625	non-GC1	2
GCF_005819635	non-GC1	2
GCF_005819645	non-GC1	2
GCF_005819685	non-GC1	85
GCF_005819705	non-GC1	2
GCF_005819725	non-GC1	2
GCF_005819755	non-GC1	2
GCF_005819765	non-GC1	2
GCF_005863285	non-GC1	2
GCF_005863305	non-GC1	2
GCF_005863345	non-GC1	2
GCF_005863355	non-GC1	2
GCF_005863385	non-GC1	187
GCF_005863395	non-GC1	2
GCF_005863405	non-GC1	2
GCF_005863415	non-GC1	2
GCF_005863455	non-GC1	2
GCF_005863535	non-GC1	2
GCF_006365355	non-GC1	2
GCF_006365365	non-GC1	2
GCF_006365375	non-GC1	2
GCF_006365385	non-GC1	2
GCF_006365405	non-GC1	2
GCF_006365455	non-GC1	2
GCF_006365465	non-GC1	2
GCF_006365495	non-GC1	2
GCF_006365515	non-GC1	221
GCF_006365525	non-GC1	2
GCF_006365555	non-GC1	2
GCF_006365565	non-GC1	2
GCF_006365575	non-GC1	2
GCF_006365585	non-GC1	46
GCF_006365635	non-GC1	2
GCF_006365655	non-GC1	2
GCF_006365665	non-GC1	2
GCF_006365675	non-GC1	2
GCF_006365685	non-GC1	2
GCF_006365735	non-GC1	2
GCF_006365745	non-GC1	2
GCF_006365775	non-GC1	2
GCF_006365795	non-GC1	2
GCF_006365805	non-GC1	2
GCF_006365835	non-GC1	2
GCF_006365845	non-GC1	2

GCF_006365865	non-GC1	2
GCF_006365885	non-GC1	2
GCF_006365895	non-GC1	2
GCF_006365935	non-GC1	2
GCF_006365945	non-GC1	2
GCF_006365955	non-GC1	2
GCF_006365965	non-GC1	2
GCF_006365975	non-GC1	2
GCF_006366035	non-GC1	2
GCF_006366055	non-GC1	2
GCF_006366065	non-GC1	2
GCF_006366085	non-GC1	2
GCF_006366115	non-GC1	2
GCF_006366125	non-GC1	2
GCF_006366135	non-GC1	2
GCF_006366155	non-GC1	2
GCF_006366195	non-GC1	2
GCF_006366215	non-GC1	2
GCF_006366235	non-GC1	2
GCF_006366245	non-GC1	2
GCF_006366275	non-GC1	2
GCF_006366285	non-GC1	2
GCF_006366315	non-GC1	2
GCF_006366335	non-GC1	142
GCF_006366355	non-GC1	2
GCF_006366365	non-GC1	2
GCF_006366385	non-GC1	2
GCF_006366405	non-GC1	2
GCF_006366435	non-GC1	2
GCF_006366445	non-GC1	2
GCF_006366465	non-GC1	2
GCF_006366475	non-GC1	2
GCF_006366515	non-GC1	2
GCF_006366535	non-GC1	2
GCF_006366545	non-GC1	2
GCF_006366575	non-GC1	2
GCF_006366585	non-GC1	2
GCF_006366595	non-GC1	2
GCF_006366635	non-GC1	2
GCF_006366645	non-GC1	2
GCF_006366665	non-GC1	2
GCF_006366685	non-GC1	2
GCF_006366715	non-GC1	2
GCF_006366735	non-GC1	46
GCF_006366745	non-GC1	2
GCF_006366755	non-GC1	2

GCF_006366785	non-GC1	2
GCF_006366805	non-GC1	2
GCF_006366825	non-GC1	2
GCF_006366845	non-GC1	2
GCF_006366875	non-GC1	2
GCF_006366895	non-GC1	2
GCF_006366905	non-GC1	2
GCF_006366925	non-GC1	2
GCF_006366935	non-GC1	2
GCF_006366975	non-GC1	2
GCF_006366985	non-GC1	2
GCF_006367015	non-GC1	2
GCF_006367025	non-GC1	2
GCF_006367045	non-GC1	2
GCF_006367055	non-GC1	2
GCF_006367085	non-GC1	2
GCF_006367115	non-GC1	2
GCF_006367125	non-GC1	46
GCF_006367135	non-GC1	2
GCF_006367175	non-GC1	2
GCF_006367195	non-GC1	2
GCF_006367205	non-GC1	2
GCF_006367225	non-GC1	2
GCF_006367245	non-GC1	2
GCF_006367275	non-GC1	2
GCF_006367285	non-GC1	2
GCF_006367295	non-GC1	2
GCF_006367335	non-GC1	2
GCF_006367345	non-GC1	2
GCF_006367375	non-GC1	2
GCF_006367395	non-GC1	213
GCF_006367405	non-GC1	2
GCF_006367435	non-GC1	2
GCF_006367475	non-GC1	2
GCF_006367485	non-GC1	2
GCF_006367505	non-GC1	2
GCF_006367515	non-GC1	2
GCF_006367535	non-GC1	2
GCF_006367575	non-GC1	2
GCF_006367595	non-GC1	2
GCF_006367605	non-GC1	2
GCF_006367615	non-GC1	2
GCF_006367625	non-GC1	2
GCF_006367675	non-GC1	2
GCF_006367685	non-GC1	2
GCF_006367695	non-GC1	2

GCF_006367735	non-GC1	2
GCF_006367745	non-GC1	2
GCF_006367765	non-GC1	2
GCF_006367795	non-GC1	2
GCF_006367805	non-GC1	2
GCF_006367835	non-GC1	2
GCF_006367845	non-GC1	2
GCF_006367855	non-GC1	221
GCF_006367895	non-GC1	2
GCF_006367905	non-GC1	2
GCF_006367915	non-GC1	2
GCF_006367945	non-GC1	2
GCF_006367965	non-GC1	2
GCF_006367995	non-GC1	2
GCF_006368005	non-GC1	2
GCF_006368025	non-GC1	2
GCF_006368045	non-GC1	2
GCF_006368055	non-GC1	2
GCF_006368095	non-GC1	2
GCF_006368105	non-GC1	2
GCF_006368125	non-GC1	2
GCF_006368155	non-GC1	2
GCF_006368165	non-GC1	922
GCF_006369695	non-GC1	2
GCF_006369735	non-GC1	2
GCF_006369755	non-GC1	2
GCF_006369795	non-GC1	2
GCF_006369805	non-GC1	2
GCF_006369815	non-GC1	2
GCF_006369835	non-GC1	2
GCF_006369845	non-GC1	2
GCF_006376725	non-GC1	2
GCF_006491865	non-GC1	49
GCF_006491875	non-GC1	154
GCF_006491885	non-GC1	32
GCF_006491955	non-GC1	239
GCF_006491975	non-GC1	23
GCF_006492075	non-GC1	2
GCF_006492085	non-GC1	195
GCF_006492125	non-GC1	417
GCF_006492155	non-GC1	2
GCF_006492165	non-GC1	415
GCF_006492175	non-GC1	32
GCF_006492215	non-GC1	155
GCF_006492245	non-GC1	2
GCF_006492265	non-GC1	25

GCF_006492295	non-GC1	203
GCF_006492305	non-GC1	2
GCF_006492315	non-GC1	1130
GCF_006492335	non-GC1	25
GCF_006492375	non-GC1	10
GCF_006492385	non-GC1	2
GCF_006492395	non-GC1	636
GCF_006492405	non-GC1	412
GCF_006492425	non-GC1	307
GCF_006492475	non-GC1	388
GCF_006492495	non-GC1	54
GCF_006492505	non-GC1	973
GCF_006492515	non-GC1	150
GCF_006492585	non-GC1	215
GCF_006492615	non-GC1	126
GCF_006492625	non-GC1	2
GCF_006492635	non-GC1	25
GCF_006492675	non-GC1	2
**GCF_006492685**	**non-GC1**	**113**
GCF_006492705	non-GC1	111
GCF_006492715	non-GC1	665
GCF_006492775	non-GC1	32
GCF_006492785	non-GC1	32
GCF_006492795	non-GC1	499
GCF_006492805	non-GC1	25
GCF_006492855	non-GC1	1093
GCF_006492865	non-GC1	78
GCF_006492885	non-GC1	963
GCF_006492905	non-GC1	412
GCF_006492925	non-GC1	2
GCF_006492945	non-GC1	338
GCF_006492965	non-GC1	1095
GCF_006492985	non-GC1	498
GCF_006493005	non-GC1	40
GCF_006493035	non-GC1	32
GCF_006493045	non-GC1	48
GCF_006493645	non-GC1	1108
GCF_006493655	non-GC1	2
GCF_006493685	non-GC1	16
GCF_006493705	non-GC1	136
GCF_006493735	non-GC1	79
GCF_006493745	non-GC1	2
GCF_006493755	non-GC1	2
GCF_006493765	non-GC1	229
GCF_006493815	non-GC1	2
GCF_006493835	non-GC1	10

GCF_006493845	non-GC1	537
GCF_006493855	non-GC1	56
GCF_006493875	non-GC1	428
GCF_006493915	non-GC1	107
GCF_006493925	non-GC1	2
GCF_006493945	non-GC1	40
GCF_006493955	non-GC1	240
GCF_006493975	non-GC1	193
GCF_006494005	non-GC1	3
GCF_006494015	non-GC1	250
GCF_006494055	non-GC1	464
GCF_006494065	non-GC1	632
GCF_006494075	non-GC1	515
GCF_006494085	non-GC1	1126
GCF_006494115	non-GC1	403
GCF_006494175	non-GC1	622
GCF_006494225	non-GC1	1118
GCF_006494255	non-GC1	79
GCF_006494265	non-GC1	108
GCF_006494285	non-GC1	374
GCF_006494305	non-GC1	267
GCF_006494565	non-GC1	85
GCF_006494575	non-GC1	106
GCF_006494585	non-GC1	10
GCF_006494615	non-GC1	972
GCF_006494665	non-GC1	136
GCF_006658565	non-GC1	15
GCF_006658595	non-GC1	103
GCF_006937715	non-GC1	2
GCF_006937725	non-GC1	2
GCF_007107525	non-GC1	49
GCF_007113605	non-GC1	2
GCF_007113615	non-GC1	2
GCF_007113625	non-GC1	2
GCF_007113635	non-GC1	2
GCF_007113645	non-GC1	2
GCF_007113705	non-GC1	2
GCF_007113715	non-GC1	2
GCF_007113765	non-GC1	2
GCF_007113785	non-GC1	2
GCF_007113795	non-GC1	2
GCF_007113815	non-GC1	2
GCF_007113845	non-GC1	2
GCF_007113875	non-GC1	2
GCF_007115055	non-GC1	2
GCF_007197635	non-GC1	49

GCF_007197865	non-GC1	2
GCF_007221455	non-GC1	2
GCF_007647215	non-GC1	2
GCF_007713645	non-GC1	2
GCF_007713655	non-GC1	2
GCF_007833155	non-GC1	109
GCF_007833195	non-GC1	1302
GCF_007999045	non-GC1	2
GCF_007999055	non-GC1	2
GCF_007999075	non-GC1	2
GCF_007999145	non-GC1	2
GCF_007999155	non-GC1	2
GCF_007999165	non-GC1	2
GCF_007999175	non-GC1	2
GCF_007999225	non-GC1	2
GCF_007999255	non-GC1	2
GCF_007999305	non-GC1	2
GCF_007999345	non-GC1	2
GCF_007999365	non-GC1	2
GCF_007999375	non-GC1	2
GCF_007999395	non-GC1	2
GCF_007999405	non-GC1	2
GCF_007999425	non-GC1	2
GCF_007999465	non-GC1	2
GCF_007999485	non-GC1	2
GCF_007999515	non-GC1	2
GCF_007999525	non-GC1	2
GCF_007999555	non-GC1	2
GCF_007999585	non-GC1	2
GCF_007999605	non-GC1	2
GCF_007999625	non-GC1	2
GCF_007999635	non-GC1	2
GCF_007999645	non-GC1	2
GCF_007999665	non-GC1	2
GCF_007999705	non-GC1	2
GCF_007999725	non-GC1	2
GCF_007999745	non-GC1	2
GCF_007999755	non-GC1	2
GCF_007999765	non-GC1	2
GCF_007999805	non-GC1	2
GCF_007999815	non-GC1	2
GCF_007999855	non-GC1	2
GCF_007999875	non-GC1	2
GCF_007999885	non-GC1	2
GCF_007999905	non-GC1	2
GCF_007999945	non-GC1	2

GCF_007999955	non-GC1	2
GCF_007999965	non-GC1	2
GCF_007999985	non-GC1	2
GCF_008000025	non-GC1	2
GCF_008000045	non-GC1	2
GCF_008000065	non-GC1	2
GCF_008000075	non-GC1	2
GCF_008000085	non-GC1	2
GCF_008000095	non-GC1	2
GCF_008000125	non-GC1	2
GCF_008000175	non-GC1	2
GCF_008000185	non-GC1	221
GCF_008000235	non-GC1	2
GCF_008000275	non-GC1	2
GCF_008000715	non-GC1	2
GCF_008000725	non-GC1	2
GCF_008033255	non-GC1	2
GCF_008123485	non-GC1	10
GCF_008123545	non-GC1	10
GCF_008124045	non-GC1	294
GCF_008180145	non-GC1	730
GCF_008244865	non-GC1	2
GCF_008244885	non-GC1	2
GCF_008244905	non-GC1	2
GCF_008477565	non-GC1	15
GCF_008581385	non-GC1	213
GCF_008581485	non-GC1	152
GCF_008581605	non-GC1	237
GCF_008581715	non-GC1	152
GCF_008581785	non-GC1	152
GCF_008629755	non-GC1	2
GCF_008629945	non-GC1	2
GCF_008630895	non-GC1	437
GCF_008632635	non-GC1	46
GCF_008709565	non-GC1	2
GCF_008709585	non-GC1	10
GCF_008709665	non-GC1	2
GCF_008709675	non-GC1	10
GCF_008709685	non-GC1	575
GCF_008709745	non-GC1	2
GCF_008726135	non-GC1	2
GCF_008801795	non-GC1	46
GCF_008802935	non-GC1	203
GCF_008806435	non-GC1	2
GCF_008806475	non-GC1	2
GCF_008806485	non-GC1	2

GCF_008806505	non-GC1	2
GCF_008806535	non-GC1	638
GCF_008807055	non-GC1	2
GCF_008830715	non-GC1	25
GCF_008830785	non-GC1	2
GCF_008830795	non-GC1	2
GCF_008830805	non-GC1	2
GCF_008830835	non-GC1	2
GCF_008830855	non-GC1	2
GCF_008830885	non-GC1	1230
GCF_008830895	non-GC1	2
GCF_008830905	non-GC1	2
GCF_008830945	non-GC1	2
GCF_008830955	non-GC1	2
GCF_008830985	non-GC1	2
GCF_008830995	non-GC1	2
GCF_008831005	non-GC1	2
GCF_008931365	non-GC1	1547
GCF_009035845	non-GC1	52
GCF_009361735	non-GC1	575
GCF_009445385	non-GC1	78
GCF_009445395	non-GC1	78
GCF_009445415	non-GC1	78
GCF_009455505	non-GC1	2
GCF_009497995	non-GC1	422
GCF_009498335	non-GC1	2
GCF_009645235	non-GC1	25
GCF_009645275	non-GC1	25
GCF_009645285	non-GC1	25
GCF_009645335	non-GC1	25
GCF_009645345	non-GC1	25
GCF_009645365	non-GC1	25
GCF_009645395	non-GC1	25
GCF_009645415	non-GC1	25
GCF_009645425	non-GC1	25
GCF_009645465	non-GC1	25
GCF_009645495	non-GC1	25
GCF_009645505	non-GC1	25
GCF_009645525	non-GC1	25
GCF_009645575	non-GC1	25
GCF_009645595	non-GC1	25
GCF_009645605	non-GC1	25
GCF_009645635	non-GC1	25
GCF_009645645	non-GC1	25
GCF_009645675	non-GC1	25
GCF_009645685	non-GC1	25

GCF_009645705	non-GC1	25
GCF_009645745	non-GC1	25
GCF_009645775	non-GC1	25
GCF_009645805	non-GC1	25
GCF_009645815	non-GC1	25
GCF_009645825	non-GC1	25
GCF_009645905	non-GC1	25
GCF_009645915	non-GC1	25
GCF_009645925	non-GC1	25
GCF_009645975	non-GC1	25
GCF_009645995	non-GC1	25
GCF_009646005	non-GC1	25
GCF_009646015	non-GC1	25
GCF_009710715	non-GC1	636
GCF_009735745	non-GC1	2
GCF_009735775	non-GC1	2
GCF_009735845	non-GC1	2
GCF_009735865	non-GC1	2
GCF_009735875	non-GC1	2
GCF_009735885	non-GC1	2
GCF_009735915	non-GC1	2
GCF_009735945	non-GC1	2
GCF_009735965	non-GC1	2
GCF_009735985	non-GC1	2
GCF_009735995	non-GC1	2
GCF_009736025	non-GC1	2
GCF_009736045	non-GC1	2
GCF_009736065	non-GC1	2
GCF_009736075	non-GC1	2
GCF_009736095	non-GC1	2
GCF_009736115	non-GC1	2
GCF_009736145	non-GC1	10
GCF_009736155	non-GC1	2
GCF_009736185	non-GC1	2
GCF_009736195	non-GC1	10
GCF_009736205	non-GC1	2
GCF_009736245	non-GC1	2
GCF_009736255	non-GC1	10
GCF_009736285	non-GC1	2
GCF_009736295	non-GC1	2
GCF_009736305	non-GC1	2
GCF_009736345	non-GC1	2
GCF_009736355	non-GC1	10
GCF_009736385	non-GC1	2
GCF_009736395	non-GC1	2
GCF_009736405	non-GC1	2

GCF_009736445	non-GC1	2
GCF_009736455	non-GC1	2
GCF_009736485	non-GC1	2
GCF_009736505	non-GC1	2
GCF_009736525	non-GC1	2
GCF_009736545	non-GC1	2
GCF_009736555	non-GC1	2
GCF_009736565	non-GC1	2
GCF_009736605	non-GC1	2
GCF_009736625	non-GC1	2
GCF_009736645	non-GC1	2
GCF_009736655	non-GC1	2
GCF_009736665	non-GC1	2
GCF_009736705	non-GC1	2
GCF_009736725	non-GC1	2
GCF_009736745	non-GC1	2
GCF_009736755	non-GC1	2
GCF_009736765	non-GC1	2
GCF_009736805	non-GC1	2
GCF_009736815	non-GC1	2
GCF_009736845	non-GC1	2
GCF_009736865	non-GC1	2
GCF_009736875	non-GC1	2
GCF_009736905	non-GC1	2
GCF_009736925	non-GC1	2
GCF_009736935	non-GC1	2
GCF_009736965	non-GC1	2
GCF_009736975	non-GC1	2
GCF_009736995	non-GC1	2
GCF_009737245	non-GC1	2
GCF_009737295	non-GC1	2
GCF_009737325	non-GC1	2
GCF_009737345	non-GC1	2
GCF_009737365	non-GC1	2
GCF_009737375	non-GC1	2
GCF_009737385	non-GC1	2
GCF_009737425	non-GC1	2
GCF_009737435	non-GC1	2
GCF_009737465	non-GC1	2
GCF_009737475	non-GC1	2
GCF_009737505	non-GC1	2
GCF_009737525	non-GC1	2
GCF_009737545	non-GC1	2
GCF_009737565	non-GC1	2
GCF_009737575	non-GC1	2
GCF_009737595	non-GC1	2

GCF_009737625	non-GC1	2
GCF_009737635	non-GC1	2
GCF_009737665	non-GC1	2
GCF_009737675	non-GC1	2
GCF_009737685	non-GC1	2
GCF_009737695	non-GC1	2
GCF_009737735	non-GC1	193
GCF_009737765	non-GC1	2
GCF_009737775	non-GC1	2
GCF_009737785	non-GC1	2
GCF_009737795	non-GC1	2
GCF_009737805	non-GC1	2
GCF_009737865	non-GC1	2
GCF_009737885	non-GC1	2
GCF_009737895	non-GC1	2
GCF_009737905	non-GC1	2
GCF_009737915	non-GC1	2
GCF_009737925	non-GC1	2
GCF_009737985	non-GC1	2
GCF_009738015	non-GC1	2
GCF_009738065	non-GC1	2
GCF_009740145	non-GC1	150
GCF_009753645	non-GC1	2
GCF_009753665	non-GC1	2
GCF_009753685	non-GC1	2
GCF_009753745	non-GC1	2
GCF_009753775	non-GC1	23
GCF_009753845	non-GC1	2
GCF_009753855	non-GC1	2
GCF_009753885	non-GC1	2
GCF_009753905	non-GC1	2
GCF_009753915	non-GC1	2
GCF_009753925	non-GC1	2
GCF_009753965	non-GC1	2
GCF_009753995	non-GC1	2
GCF_009754005	non-GC1	2
GCF_009754985	non-GC1	374
GCF_009758295	non-GC1	2
GCF_009758335	non-GC1	2
GCF_009758395	non-GC1	2
GCF_009759685	non-GC1	52
GCF_009759905	non-GC1	2
GCF_009759925	non-GC1	2
GCF_009759935	non-GC1	2
GCF_009759985	non-GC1	2
GCF_009759995	non-GC1	2

GCF_009760005	non-GC1	2
GCF_009760015	non-GC1	2
GCF_009760065	non-GC1	2
GCF_009760075	non-GC1	2
GCF_009760085	non-GC1	2
GCF_009760105	non-GC1	2
GCF_009760145	non-GC1	2
GCF_009760155	non-GC1	2
GCF_009760165	non-GC1	2
GCF_009760175	non-GC1	2
GCF_009760225	non-GC1	2
GCF_009760235	non-GC1	2
GCF_009760245	non-GC1	2
GCF_009760285	non-GC1	2
GCF_009760305	non-GC1	2
GCF_009760315	non-GC1	2
GCF_009760325	non-GC1	2
GCF_009760365	non-GC1	2
GCF_009760385	non-GC1	2
GCF_009760445	non-GC1	2
GCF_009760455	non-GC1	2
GCF_009760525	non-GC1	2
GCF_009760535	non-GC1	2
GCF_009760565	non-GC1	2
GCF_009760575	non-GC1	2
GCF_009760605	non-GC1	2
GCF_009760625	non-GC1	2
GCF_009760635	non-GC1	2
GCF_009760645	non-GC1	2
GCF_009760685	non-GC1	2
GCF_009760705	non-GC1	2
GCF_009760715	non-GC1	2
GCF_009760725	non-GC1	2
GCF_009760775	non-GC1	2
GCF_009828675	non-GC1	162
GCF_009829525	non-GC1	79
GCF_009829535	non-GC1	79
GCF_009829555	non-GC1	1448
GCF_009829595	non-GC1	25
GCF_009829755	non-GC1	15
GCF_009833005	non-GC1	825
GCF_009857695	non-GC1	126
GCF_009857705	non-GC1	10
GCF_009887795	non-GC1	400
GCF_009887985	non-GC1	78
GCF_009888065	non-GC1	400

GCF_009898245	non-GC1	15
GCF_009901665	non-GC1	400
GCF_009910195	non-GC1	79
GCF_009910215	non-GC1	79
GCF_010178615	non-GC1	2
GCF_010178625	non-GC1	2
GCF_010178645	non-GC1	2
GCF_010178695	non-GC1	2
GCF_010178715	non-GC1	2
GCF_010178725	non-GC1	2
GCF_010178735	non-GC1	2
GCF_010178765	non-GC1	2
GCF_010178795	non-GC1	187
GCF_010178815	non-GC1	2
GCF_010178825	non-GC1	2
GCF_010178835	non-GC1	2
GCF_010178855	non-GC1	2
GCF_010178895	non-GC1	2
GCF_010178925	non-GC1	2
GCF_010178945	non-GC1	2
GCF_010178955	non-GC1	2
GCF_010179015	non-GC1	2
GCF_010179035	non-GC1	2
GCF_010179055	non-GC1	2
GCF_010179065	non-GC1	2
GCF_010179075	non-GC1	2
GCF_010179105	non-GC1	2
GCF_010179135	non-GC1	2
GCF_010179145	non-GC1	25
GCF_010179175	non-GC1	2
GCF_010179185	non-GC1	2
GCF_010179205	non-GC1	2
GCF_010179235	non-GC1	2
GCF_010179245	non-GC1	2
GCF_010179275	non-GC1	2
GCF_010179285	non-GC1	2
GCF_010179295	non-GC1	2
GCF_010179335	non-GC1	2
GCF_010179355	non-GC1	2
GCF_010179375	non-GC1	2
GCF_010179385	non-GC1	2
GCF_010179395	non-GC1	187
GCF_010179435	non-GC1	2
GCF_010179455	non-GC1	2
GCF_010179475	non-GC1	2
GCF_010179495	non-GC1	149

GCF_010179515	non-GC1	2
GCF_010179525	non-GC1	25
GCF_010179545	non-GC1	2
GCF_010179575	non-GC1	2
GCF_010179585	non-GC1	2
GCF_010499635	non-GC1	2
GCF_010499675	non-GC1	2
GCF_010499685	non-GC1	2
GCF_010499705	non-GC1	2
GCF_010499735	non-GC1	25
GCF_010499775	non-GC1	10
GCF_010499785	non-GC1	103
GCF_010499805	non-GC1	2
GCF_010499875	non-GC1	2
GCF_010499885	non-GC1	2
GCF_010499935	non-GC1	2
GCF_010499945	non-GC1	1278
GCF_010499955	non-GC1	2
GCF_010499975	non-GC1	2
GCF_010500015	non-GC1	2
GCF_010500025	non-GC1	149
GCF_010500035	non-GC1	2
GCF_010500075	non-GC1	2
GCF_010500085	non-GC1	2
GCF_010500115	non-GC1	2
GCF_010500125	non-GC1	2
GCF_010500155	non-GC1	2
GCF_010500175	non-GC1	25
GCF_010500185	non-GC1	2
GCF_010500235	non-GC1	10
GCF_010500255	non-GC1	2
GCF_010500265	non-GC1	2
GCF_010500315	non-GC1	2
GCF_010500335	non-GC1	2
GCF_010500345	non-GC1	622
GCF_010500355	non-GC1	2
GCF_010500385	non-GC1	149
GCF_010500415	non-GC1	976
GCF_010500455	non-GC1	10
GCF_010500475	non-GC1	2
GCF_010500485	non-GC1	2
GCF_010500515	non-GC1	2
GCF_010500535	non-GC1	2
GCF_010500545	non-GC1	1512
GCF_010500565	non-GC1	10
GCF_010500585	non-GC1	149

GCF_010500625	non-GC1	2
GCF_010500635	non-GC1	2
GCF_010500645	non-GC1	2
GCF_010500665	non-GC1	1512
GCF_010500715	non-GC1	2
GCF_010587005	non-GC1	2
GCF_010587045	non-GC1	132
GCF_010587125	non-GC1	132
GCF_010587545	non-GC1	2
GCF_010587575	non-GC1	2
GCF_010587925	non-GC1	406
GCF_010604415	non-GC1	108
GCF_010604785	non-GC1	2
**GCF_010604795**	**non-GC1**	**2**
GCF_010604855	non-GC1	2
GCF_010604915	non-GC1	2
GCF_010605285	non-GC1	2
GCF_010607455	non-GC1	10
GCF_010607685	non-GC1	2
GCF_010607705	non-GC1	106
GCF_010608825	non-GC1	2
GCF_010608885	non-GC1	2
GCF_010609075	non-GC1	2
GCF_011067065	non-GC1	2
GCF_011601365	non-GC1	149
GCF_011601385	non-GC1	2
GCF_011601425	non-GC1	52
GCF_011601445	non-GC1	2
GCF_011601455	non-GC1	52
GCF_011601475	non-GC1	2
GCF_011601485	non-GC1	2
GCF_011601525	non-GC1	2
GCF_011601545	non-GC1	25
GCF_011601555	non-GC1	2
GCF_011601575	non-GC1	2
GCF_011601585	non-GC1	2
GCF_011601625	non-GC1	2
GCF_011601645	non-GC1	2
GCF_011601665	non-GC1	2
GCF_011601685	non-GC1	2
GCF_011601695	non-GC1	2
GCF_011601725	non-GC1	25
GCF_011601735	non-GC1	2
GCF_011601765	non-GC1	2
GCF_011601785	non-GC1	2
GCF_011601805	non-GC1	2

GCF_011601815	non-GC1	25
GCF_011601845	non-GC1	2
GCF_011601865	non-GC1	2
GCF_011601875	non-GC1	2
GCF_011601885	non-GC1	2
GCF_011601925	non-GC1	2
GCF_011601935	non-GC1	2
GCF_011601965	non-GC1	2
GCF_011601975	non-GC1	2
GCF_011601985	non-GC1	2
GCF_011602045	non-GC1	2
GCF_011602055	non-GC1	2
GCF_011602095	non-GC1	2
GCF_011602125	non-GC1	2
GCF_011602145	non-GC1	2
GCF_011602155	non-GC1	2
GCF_011602165	non-GC1	149
GCF_011602205	non-GC1	2
GCF_011602225	non-GC1	2
GCF_011602235	non-GC1	2
GCF_011602255	non-GC1	2
GCF_011602285	non-GC1	2
GCF_011602305	non-GC1	2
GCF_011602405	non-GC1	2
GCF_011602535	non-GC1	10
GCF_011602545	non-GC1	2
GCF_011602625	non-GC1	2
GCF_011602645	non-GC1	2
GCF_011602655	non-GC1	2
GCF_011602675	non-GC1	2
GCF_011602685	non-GC1	2
GCF_011602725	non-GC1	2
GCF_011602745	non-GC1	2
GCF_011602755	non-GC1	2
GCF_011603245	non-GC1	2
GCF_011603305	non-GC1	2
GCF_011603345	non-GC1	2
GCF_011603355	non-GC1	2
GCF_011603385	non-GC1	25
GCF_011603405	non-GC1	2
GCF_011603415	non-GC1	2
GCF_011603445	non-GC1	2
GCF_011603455	non-GC1	2
GCF_011603475	non-GC1	2
GCF_011603525	non-GC1	2
GCF_011603535	non-GC1	187

GCF_011603545	non-GC1	2
GCF_011603585	non-GC1	25
GCF_011603595	non-GC1	2
GCF_011603625	non-GC1	2
GCF_011603635	non-GC1	2
GCF_011603645	non-GC1	2
GCF_011603685	non-GC1	2
GCF_011603705	non-GC1	2
GCF_011603725	non-GC1	2
GCF_011603735	non-GC1	2
GCF_011603765	non-GC1	2
GCF_011603775	non-GC1	2
GCF_011603805	non-GC1	2
GCF_011603825	non-GC1	2
GCF_011603835	non-GC1	2
GCF_011603845	non-GC1	2
GCF_011603855	non-GC1	2
GCF_011603905	non-GC1	2
GCF_011603945	non-GC1	2
GCF_011603965	non-GC1	2
GCF_011683235	non-GC1	2
GCF_011683255	non-GC1	2
GCF_011683265	non-GC1	2
GCF_011683275	non-GC1	2
GCF_011683305	non-GC1	2
GCF_011683335	non-GC1	2
GCF_011683355	non-GC1	2
GCF_011683365	non-GC1	2
GCF_011683375	non-GC1	2
GCF_011683395	non-GC1	2
GCF_011683425	non-GC1	2
GCF_011683455	non-GC1	2
GCF_011683465	non-GC1	2
GCF_011683475	non-GC1	2
GCF_011683485	non-GC1	2
GCF_011683505	non-GC1	2
GCF_011683555	non-GC1	2
GCF_011683565	non-GC1	2
GCF_011683575	non-GC1	2
GCF_011683605	non-GC1	2
GCF_011683625	non-GC1	2
GCF_011683655	non-GC1	2
GCF_011683665	non-GC1	2
GCF_011745885	non-GC1	2
GCF_011754375	non-GC1	52
GCF_012037635	non-GC1	2

GCF_012037655	non-GC1	49
GCF_012272715	non-GC1	2
GCF_012272735	non-GC1	2
GCF_012272755	non-GC1	2
GCF_012272775	non-GC1	2
GCF_012272795	non-GC1	2
GCF_012276745	non-GC1	79
GCF_012277115	non-GC1	79
GCF_012524085	non-GC1	172
GCF_012524275	non-GC1	25
GCF_012781995	non-GC1	15
GCF_012931605	non-GC1	1472
GCF_012931625	non-GC1	103
GCF_012931645	non-GC1	103
GCF_012931665	non-GC1	107
GCF_012934905	non-GC1	25
GCF_012934925	non-GC1	2
GCF_012934945	non-GC1	2
GCF_012934965	non-GC1	2
GCF_012934985	non-GC1	2
GCF_012935025	non-GC1	2
GCF_012935045	non-GC1	2
GCF_012935065	non-GC1	2
GCF_012935085	non-GC1	10
GCF_012935105	non-GC1	10
GCF_012935125	non-GC1	10
GCF_012935145	non-GC1	2
GCF_012935165	non-GC1	2
GCF_012935185	non-GC1	2
GCF_012952675	non-GC1	406
GCF_012955825	non-GC1	15
GCF_012955845	non-GC1	15
GCF_012956585	non-GC1	15
GCF_012974585	non-GC1	23
GCF_012974845	non-GC1	2
GCF_013140255	non-GC1	2
GCF_013140275	non-GC1	2
GCF_013140295	non-GC1	2
GCF_013140305	non-GC1	2
GCF_013140315	non-GC1	2
GCF_013140355	non-GC1	2
GCF_013140365	non-GC1	2
GCF_013154985	non-GC1	2
GCF_013184005	non-GC1	172
GCF_013185115	non-GC1	636
GCF_013185155	non-GC1	492

GCF_013185245	non-GC1	2
GCF_013185265	non-GC1	2
GCF_013305465	non-GC1	2
GCF_013346525	non-GC1	2
GCF_013346545	non-GC1	2
GCF_013346675	non-GC1	2
GCF_013372085	non-GC1	437
GCF_013375255	non-GC1	2
GCF_013376855	non-GC1	570
GCF_013377175	non-GC1	108
GCF_013387305	non-GC1	2
GCF_013387385	non-GC1	2
GCF_013394265	non-GC1	1093
GCF_013394285	non-GC1	16
GCF_013416255	non-GC1	374
GCF_013416275	non-GC1	239
GCF_013618675	non-GC1	2
GCF_013618685	non-GC1	2
GCF_013618715	non-GC1	2
GCF_013618725	non-GC1	2
GCF_013618745	non-GC1	2
GCF_013618775	non-GC1	2
GCF_013618785	non-GC1	2
GCF_013618815	non-GC1	2
GCF_013618835	non-GC1	2
GCF_013618855	non-GC1	2
GCF_013778355	non-GC1	1479
GCF_013778405	non-GC1	2
GCF_013778425	non-GC1	16
GCF_013778445	non-GC1	16
GCF_013787185	non-GC1	203
GCF_014116765	non-GC1	52
GCF_014116785	non-GC1	52
GCF_014116795	non-GC1	52
GCF_014159355	non-GC1	213
GCF_014169655	non-GC1	1548
GCF_014171935	non-GC1	63
GCF_014218985	non-GC1	52
GCF_014267385	non-GC1	2
GCF_014322245	non-GC1	138
GCF_014334885	non-GC1	103
GCF_014530685	non-GC1	2
GCF_014530705	non-GC1	2
GCF_014530735	non-GC1	2
GCF_014530755	non-GC1	2
GCF_014530785	non-GC1	2

GCF_014530795	non-GC1	2
GCF_014530805	non-GC1	2
GCF_014530815	non-GC1	2
GCF_014530885	non-GC1	664
GCF_014530895	non-GC1	2
GCF_014530925	non-GC1	2
GCF_014530935	non-GC1	2
GCF_014530945	non-GC1	103
GCF_014530985	non-GC1	664
GCF_014531005	non-GC1	158
GCF_014531035	non-GC1	158
GCF_014531045	non-GC1	158
GCF_014531155	non-GC1	158
GCF_014531175	non-GC1	158
GCF_014531215	non-GC1	158
GCF_014531235	non-GC1	15
GCF_014531245	non-GC1	2
GCF_014531275	non-GC1	158
GCF_014531285	non-GC1	158
GCF_014531315	non-GC1	2
GCF_014531345	non-GC1	25
GCF_014531355	non-GC1	85
GCF_014531395	non-GC1	664
GCF_014531415	non-GC1	2
GCF_014531425	non-GC1	1535
GCF_014531435	non-GC1	158
GCF_014531465	non-GC1	664
GCF_014531475	non-GC1	158
GCF_014531515	non-GC1	15
GCF_014531555	non-GC1	25
GCF_014531565	non-GC1	664
GCF_014531585	non-GC1	600
GCF_014531635	non-GC1	2
GCF_014531645	non-GC1	2
GCF_014531655	non-GC1	570
GCF_014559915	non-GC1	106
GCF_014559925	non-GC1	2
GCF_014559955	non-GC1	2
GCF_014559965	non-GC1	2
GCF_014560015	non-GC1	142
GCF_014560035	non-GC1	23
GCF_014560045	non-GC1	2
GCF_014560065	non-GC1	2
GCF_014560115	non-GC1	36
GCF_014560135	non-GC1	49
GCF_014560155	non-GC1	2

GCF_014560175	non-GC1	763
GCF_014560195	non-GC1	1137
GCF_014560205	non-GC1	2
GCF_014560235	non-GC1	2
GCF_014560245	non-GC1	2
GCF_014560275	non-GC1	2
GCF_014560295	non-GC1	2
GCF_014560305	non-GC1	2
GCF_014560315	non-GC1	2
GCF_014560375	non-GC1	2
GCF_014560395	non-GC1	2
GCF_014560405	non-GC1	2
GCF_014560425	non-GC1	2
GCF_014560475	non-GC1	40
GCF_014560495	non-GC1	2
GCF_014560515	non-GC1	2
GCF_014560525	non-GC1	2
GCF_014560535	non-GC1	2
GCF_014560575	non-GC1	2
GCF_014560585	non-GC1	2
GCF_014560615	non-GC1	2
GCF_014560625	non-GC1	2
GCF_014560655	non-GC1	2
GCF_014560665	non-GC1	2
GCF_014560685	non-GC1	2
GCF_014560715	non-GC1	2
GCF_014560735	non-GC1	2
GCF_014560755	non-GC1	2
GCF_014560765	non-GC1	2
GCF_014560775	non-GC1	2
GCF_014560815	non-GC1	2
GCF_014560835	non-GC1	2
GCF_014560855	non-GC1	2
GCF_014560865	non-GC1	2
GCF_014560895	non-GC1	2
GCF_014560905	non-GC1	2
GCF_014560935	non-GC1	2
GCF_014560955	non-GC1	2
GCF_014560965	non-GC1	2
GCF_014560995	non-GC1	2
GCF_014561005	non-GC1	2
GCF_014561035	non-GC1	2
GCF_014561045	non-GC1	2
GCF_014561075	non-GC1	2
GCF_014561085	non-GC1	2
GCF_014561095	non-GC1	2

GCF_014561135	non-GC1	2
GCF_014561145	non-GC1	2
GCF_014561175	non-GC1	2
GCF_014596675	non-GC1	2
GCF_014672735	non-GC1	172
GCF_014672755	non-GC1	52
GCF_014672775	non-GC1	437
GCF_014705785	non-GC1	2
GCF_014705805	non-GC1	2
GCF_014705825	non-GC1	2
GCF_014705845	non-GC1	2
GCF_014705865	non-GC1	2
GCF_014705885	non-GC1	2
GCF_014842895	non-GC1	49
GCF_014874575	non-GC1	126
GCF_014889395	non-GC1	2
GCF_014889435	non-GC1	2
GCF_014889445	non-GC1	2
GCF_014893575	non-GC1	2
GCF_014893595	non-GC1	2
GCF_014893605	non-GC1	2
GCF_014893635	non-GC1	238
GCF_014893645	non-GC1	238
GCF_014893665	non-GC1	46
GCF_014893695	non-GC1	2
GCF_014893705	non-GC1	2
GCF_014893725	non-GC1	2
GCF_014893765	non-GC1	2
GCF_014947085	non-GC1	2
GCF_014947125	non-GC1	2
GCF_014947135	non-GC1	2
GCF_014947145	non-GC1	2
GCF_014947185	non-GC1	2
GCF_014947215	non-GC1	2
GCF_015265765	non-GC1	164
GCF_015265775	non-GC1	79
GCF_015265785	non-GC1	49
GCF_015356135	non-GC1	2
GCF_015356145	non-GC1	2
GCF_015356155	non-GC1	2
GCF_015356165	non-GC1	2
GCF_015536845	non-GC1	15
GCF_015536855	non-GC1	15
GCF_015536865	non-GC1	1448
GCF_015536875	non-GC1	79
GCF_015536925	non-GC1	15

GCF_015536935	non-GC1	79
GCF_015536955	non-GC1	79
GCF_015536965	non-GC1	730
GCF_015537035	non-GC1	25
GCF_015537045	non-GC1	15
GCF_015537085	non-GC1	25
GCF_015537095	non-GC1	79
GCF_015537125	non-GC1	15
GCF_015537145	non-GC1	25
GCF_015537155	non-GC1	79
GCF_015537175	non-GC1	79
GCF_015537245	non-GC1	25
GCF_015537255	non-GC1	15
GCF_015537275	non-GC1	79
GCF_015537295	non-GC1	79
GCF_015537345	non-GC1	15
GCF_015537365	non-GC1	79
GCF_015537385	non-GC1	1447
GCF_015537405	non-GC1	25
GCF_015537415	non-GC1	15
GCF_015537465	non-GC1	79
GCF_015537485	non-GC1	49
GCF_015537525	non-GC1	25
GCF_015537535	non-GC1	15
GCF_015537565	non-GC1	25
GCF_015537605	non-GC1	79
GCF_015537615	non-GC1	79
GCF_015537635	non-GC1	79
GCF_015537665	non-GC1	730
GCF_015537705	non-GC1	79
GCF_015537725	non-GC1	15
GCF_015537745	non-GC1	15
GCF_015537765	non-GC1	163
GCF_015537775	non-GC1	15
GCF_015537825	non-GC1	25
GCF_015537845	non-GC1	730
GCF_015537855	non-GC1	15
GCF_015537995	non-GC1	79
GCF_015538025	non-GC1	25
GCF_015538045	non-GC1	15
GCF_015538085	non-GC1	15
GCF_015538105	non-GC1	79
GCF_015538135	non-GC1	15
GCF_015538155	non-GC1	151
GCF_015538165	non-GC1	79
GCF_015538205	non-GC1	25

GCF_015538225	non-GC1	15
GCF_015538255	non-GC1	79
GCF_015538305	non-GC1	25
GCF_015538325	non-GC1	79
GCF_015538345	non-GC1	15
GCF_015627265	non-GC1	187
GCF_015627285	non-GC1	2
GCF_015627295	non-GC1	187
GCF_015627315	non-GC1	745
GCF_015627365	non-GC1	187
GCF_015627385	non-GC1	745
GCF_015666275	non-GC1	79
GCF_015666365	non-GC1	79
GCF_015666475	non-GC1	79
GCF_015666535	non-GC1	25
GCF_015676635	non-GC1	375
GCF_015687185	non-GC1	2
GCF_015694245	non-GC1	2
GCF_015694505	non-GC1	2
GCF_015694555	non-GC1	2
GCF_015694565	non-GC1	2
GCF_015694625	non-GC1	218
GCF_015694645	non-GC1	2
GCF_015694655	non-GC1	2
GCF_015694675	non-GC1	2
GCF_015708195	non-GC1	2
GCF_015731955	non-GC1	2
GCF_015731965	non-GC1	2
GCF_015731975	non-GC1	2
GCF_015731985	non-GC1	1555
GCF_015732055	non-GC1	1555
GCF_015732075	non-GC1	1555
GCF_015732435	non-GC1	1555
GCF_015870765	non-GC1	570
GCF_015870965	non-GC1	570
GCF_015910475	non-GC1	438
GCF_016054925	non-GC1	848
GCF_016054975	non-GC1	78
GCF_016055015	non-GC1	79
GCF_016055335	non-GC1	79
GCF_016055375	non-GC1	79
GCF_016055475	non-GC1	79
GCF_016055485	non-GC1	79
GCF_016055595	non-GC1	79
GCF_016055685	non-GC1	79
GCF_016055835	non-GC1	79

GCF_016056045	non-GC1	79
GCF_016056095	non-GC1	25
GCF_016056145	non-GC1	79
GCF_016125615	non-GC1	374
GCF_016125625	non-GC1	239
GCF_016125655	non-GC1	374
GCF_016125715	non-GC1	239
GCF_016350005	non-GC1	2
GCF_016350065	non-GC1	2
GCF_016350085	non-GC1	2
GCF_016350105	non-GC1	2
GCF_016505985	non-GC1	911
GCF_016506005	non-GC1	132
GCF_016506045	non-GC1	57
GCF_016506055	non-GC1	2
GCF_016506135	non-GC1	2
GCF_016506195	non-GC1	2
GCF_016506215	non-GC1	2
GCF_016506225	non-GC1	2
GCF_016506265	non-GC1	499
GCF_016506305	non-GC1	2
GCF_016506335	non-GC1	2
GCF_016506385	non-GC1	221
GCF_016506405	non-GC1	2
GCF_016506425	non-GC1	2
GCF_016506435	non-GC1	2
GCF_016506445	non-GC1	150
GCF_016506455	non-GC1	2
GCF_016506505	non-GC1	221
GCF_016506525	non-GC1	46
GCF_016506535	non-GC1	54
GCF_016506545	non-GC1	2
GCF_016506605	non-GC1	2
GCF_016506625	non-GC1	164
GCF_016506645	non-GC1	2
GCF_016506655	non-GC1	2
GCF_016506665	non-GC1	1573
GCF_016506685	non-GC1	2
GCF_016506725	non-GC1	2
GCF_016506735	non-GC1	2
GCF_016506765	non-GC1	46
GCF_016506775	non-GC1	2
GCF_016506795	non-GC1	2
GCF_016506825	non-GC1	32
GCF_016506845	non-GC1	1191
GCF_016506855	non-GC1	2

GCF_016506875	non-GC1	2
GCF_016506895	non-GC1	348
GCF_016506925	non-GC1	2
GCF_016506945	non-GC1	2
GCF_016506955	non-GC1	2
GCF_016506985	non-GC1	406
GCF_016507005	non-GC1	406
GCF_016507015	non-GC1	2
**GCF_016507035**	**non-GC1**	**2**
GCF_016507055	non-GC1	2
GCF_016507085	non-GC1	342
GCF_016508295	non-GC1	2
GCF_016508325	non-GC1	2
GCF_016508385	non-GC1	46
GCF_016508455	non-GC1	1252
GCF_016508505	non-GC1	348
GCF_016508565	non-GC1	348
GCF_016508585	non-GC1	1258
GCF_016508635	non-GC1	1190
GCF_016613495	non-GC1	78
GCF_016613515	non-GC1	78
GCF_016613525	non-GC1	78
GCF_016613575	non-GC1	78
GCF_016613595	non-GC1	78
GCF_016613615	non-GC1	78
GCF_016613625	non-GC1	78
GCF_016613645	non-GC1	78
GCF_016613675	non-GC1	78
GCF_016613695	non-GC1	78
GCF_016613715	non-GC1	78
GCF_016635075	non-GC1	388
GCF_016654095	non-GC1	78
GCF_016654105	non-GC1	78
GCF_016654155	non-GC1	2
GCF_016654165	non-GC1	25
GCF_016654175	non-GC1	78
GCF_016654215	non-GC1	45
GCF_016654225	non-GC1	2
GCF_016654255	non-GC1	15
GCF_016654265	non-GC1	1487
GCF_016654295	non-GC1	911
GCF_016654335	non-GC1	2
GCF_016654345	non-GC1	78
GCF_016654365	non-GC1	45
GCF_016654375	non-GC1	2
GCF_016745455	non-GC1	2

GCF_016745475	non-GC1	2
GCF_016745515	non-GC1	2
GCF_016745555	non-GC1	2
GCF_016745575	non-GC1	2
GCF_016745585	non-GC1	2
GCF_016745615	non-GC1	2
GCF_016745625	non-GC1	2
GCF_016745655	non-GC1	2
GCF_016745675	non-GC1	2
GCF_016745695	non-GC1	2
GCF_016745715	non-GC1	2
GCF_016745735	non-GC1	2
GCF_016745755	non-GC1	2
GCF_016745775	non-GC1	2
GCF_016745795	non-GC1	2
GCF_016745805	non-GC1	2
GCF_016745855	non-GC1	2
GCF_016745915	non-GC1	2
GCF_016745935	non-GC1	2
GCF_016745955	non-GC1	2
GCF_016745975	non-GC1	2
GCF_016745995	non-GC1	2
GCF_016746015	non-GC1	2
GCF_016746035	non-GC1	2
GCF_016746055	non-GC1	2
GCF_016746075	non-GC1	2
GCF_016762815	non-GC1	78
GCF_016762825	non-GC1	78
GCF_016762895	non-GC1	78
GCF_016864295	non-GC1	1077
GCF_016864315	non-GC1	78
GCF_016864915	non-GC1	78
GCF_016865465	non-GC1	571
GCF_016888585	non-GC1	2
GCF_016901075	non-GC1	2
GCF_016901145	non-GC1	2
GCF_016903135	non-GC1	2
GCF_016903215	non-GC1	2
GCF_017090145	non-GC1	52
GCF_017096325	non-GC1	2
GCF_017096345	non-GC1	2
GCF_017096365	non-GC1	2
GCF_017096385	non-GC1	2
GCF_017096405	non-GC1	2
GCF_017096425	non-GC1	2
GCF_017098185	non-GC1	2

GCF_017098205	non-GC1	2
GCF_017098225	non-GC1	2
GCF_017098245	non-GC1	2
GCF_017098265	non-GC1	2
GCF_017168535	non-GC1	374
GCF_017168795	non-GC1	2
GCF_017168905	non-GC1	164
GCF_017169085	non-GC1	2
GCF_017169105	non-GC1	2
GCF_017169125	non-GC1	164
GCF_017169155	non-GC1	2
GCF_017169175	non-GC1	2
GCF_017169215	non-GC1	2
GCF_017169275	non-GC1	2
GCF_017169415	non-GC1	2
GCF_017169435	non-GC1	2
GCF_017169455	non-GC1	2
GCF_017169465	non-GC1	2
GCF_017287975	non-GC1	2
GCF_017292175	non-GC1	85
GCF_017302955	non-GC1	2
GCF_017349005	non-GC1	2
GCF_017349055	non-GC1	25
GCF_017349095	non-GC1	2
GCF_017349115	non-GC1	265
GCF_017349135	non-GC1	2
GCF_017349155	non-GC1	721
GCF_017349175	non-GC1	2
GCF_017349195	non-GC1	2
GCF_017349215	non-GC1	2
GCF_017349235	non-GC1	164
GCF_017349255	non-GC1	2
GCF_017349275	non-GC1	77
GCF_017581665	non-GC1	2
GCF_017581825	non-GC1	52
GCF_017582285	non-GC1	2
GCF_017582345	non-GC1	52
GCF_017582535	non-GC1	52
GCF_017639875	non-GC1	622
GCF_017723975	non-GC1	622
GCF_017724115	non-GC1	622
GCF_017724155	non-GC1	622
GCF_017724195	non-GC1	622
GCF_017724215	non-GC1	622
GCF_017726495	non-GC1	622
GCF_017726555	non-GC1	622

GCF_017726575	non-GC1	374
GCF_017742855	non-GC1	622
GCF_017753625	non-GC1	1554
GCF_017838055	non-GC1	2
GCF_017838085	non-GC1	2
GCF_017838165	non-GC1	2
GCF_017838235	non-GC1	85
GCF_017897325	non-GC1	2
GCF_017897365	non-GC1	2
GCF_017897385	non-GC1	2
GCF_017897405	non-GC1	2
GCF_017897445	non-GC1	78
GCF_017897485	non-GC1	2
GCF_017897505	non-GC1	2
GCF_017897535	non-GC1	2
GCF_017897645	non-GC1	2
GCF_017897655	non-GC1	2
GCF_017897685	non-GC1	2
GCF_017897725	non-GC1	2
GCF_017897745	non-GC1	2
GCF_017897755	non-GC1	2
GCF_017909485	non-GC1	1083
GCF_017909495	non-GC1	1083
GCF_017939285	non-GC1	1230
GCF_017965745	non-GC1	2
GCF_017965785	non-GC1	2
GCF_018069945	non-GC1	2
GCF_018069965	non-GC1	2
GCF_018069985	non-GC1	78
GCF_018069995	non-GC1	78
GCF_018105825	non-GC1	2
GCF_018105875	non-GC1	2
GCF_018105895	non-GC1	2
GCF_018105925	non-GC1	2
GCF_018105945	non-GC1	2
GCF_018105965	non-GC1	25
GCF_018106015	non-GC1	2
GCF_018106035	non-GC1	2
GCF_018106055	non-GC1	2
GCF_018106075	non-GC1	164
GCF_018106095	non-GC1	2
GCF_018106125	non-GC1	2
GCF_018135585	non-GC1	221
GCF_018156045	non-GC1	318
GCF_018156055	non-GC1	109
GCF_018156065	non-GC1	876

GCF_018156145	non-GC1	15
GCF_018156745	non-GC1	79
GCF_018221345	non-GC1	3
GCF_018221365	non-GC1	3
GCF_018221375	non-GC1	2
GCF_018221385	non-GC1	3
GCF_018221425	non-GC1	3
GCF_018361325	non-GC1	107
GCF_018420235	non-GC1	78
GCF_018420315	non-GC1	25
GCF_018420785	non-GC1	78
GCF_018420825	non-GC1	2
GCF_018421845	non-GC1	77
GCF_018422255	non-GC1	636
GCF_018422325	non-GC1	636
GCF_018422715	non-GC1	2
GCF_018422775	non-GC1	2
GCF_018422795	non-GC1	2
GCF_018423485	non-GC1	636
GCF_018440955	non-GC1	2
GCF_018441385	non-GC1	25
GCF_018441465	non-GC1	400
GCF_018441985	non-GC1	2
GCF_018442085	non-GC1	764
GCF_018442105	non-GC1	2
GCF_018442385	non-GC1	2
GCF_018442465	non-GC1	2
GCF_018442635	non-GC1	85
GCF_018443085	non-GC1	2
GCF_018443095	non-GC1	2
GCF_018443625	non-GC1	636
GCF_018443735	non-GC1	2
GCF_018443775	non-GC1	636
GCF_018443825	non-GC1	636
GCF_018443875	non-GC1	636
GCF_018443945	non-GC1	636
GCF_018443975	non-GC1	636
GCF_018444145	non-GC1	2
GCF_018444235	non-GC1	604
GCF_018444305	non-GC1	636
GCF_018444405	non-GC1	636
GCF_018444525	non-GC1	636
GCF_018444555	non-GC1	2
GCF_018445045	non-GC1	2
GCF_018445075	non-GC1	2
GCF_018445085	non-GC1	2

GCF_018445145	non-GC1	2
GCF_018445265	non-GC1	2
GCF_018445285	non-GC1	2
GCF_018445315	non-GC1	2
GCF_018445345	non-GC1	2
GCF_018445375	non-GC1	2
GCF_018446635	non-GC1	664
GCF_018446675	non-GC1	164
GCF_018447265	non-GC1	2
GCF_018454385	non-GC1	2
GCF_018499935	non-GC1	2
GCF_018604365	non-GC1	2
GCF_018678125	non-GC1	370
GCF_018788675	non-GC1	2
GCF_018788695	non-GC1	215
GCF_018788705	non-GC1	2
GCF_018788775	non-GC1	2
GCF_018788795	non-GC1	604
GCF_018788815	non-GC1	2
GCF_018788835	non-GC1	2
GCF_018788855	non-GC1	158
GCF_018788895	non-GC1	2
GCF_018788905	non-GC1	2
GCF_018788965	non-GC1	2
GCF_018789005	non-GC1	78
GCF_018789045	non-GC1	2
GCF_018789075	non-GC1	2
GCF_018789085	non-GC1	2
GCF_018789135	non-GC1	2
GCF_018789185	non-GC1	2
GCF_018789235	non-GC1	636
GCF_018789275	non-GC1	2
GCF_018789295	non-GC1	2
GCF_018789335	non-GC1	2
GCF_018789355	non-GC1	636
GCF_018789395	non-GC1	2
GCF_018789415	non-GC1	2
GCF_018789435	non-GC1	2
GCF_018789475	non-GC1	636
GCF_018789495	non-GC1	85
GCF_018789505	non-GC1	2
GCF_018789525	non-GC1	2
GCF_018789555	non-GC1	2
GCF_018789575	non-GC1	2
GCF_018789595	non-GC1	636
GCF_018789615	non-GC1	636

GCF_018789635	non-GC1	2
GCF_018789655	non-GC1	2
GCF_018791845	non-GC1	636
GCF_018808925	non-GC1	25
GCF_018831125	non-GC1	25
GCF_018863315	non-GC1	428
GCF_018883565	non-GC1	624
GCF_018928075	non-GC1	2
GCF_018928095	non-GC1	2
GCF_018928105	non-GC1	2
GCF_018928135	non-GC1	2
GCF_018928145	non-GC1	492
GCF_018928175	non-GC1	2
GCF_018928195	non-GC1	2
GCF_018928215	non-GC1	2
GCF_018986615	non-GC1	2
GCF_018986625	non-GC1	2
GCF_019076685	non-GC1	142
GCF_900010325	non-GC1	570
GCF_900010465	non-GC1	1550
GCF_900011275	non-GC1	16
GCF_900011285	non-GC1	10
GCF_900011295	non-GC1	52
GCF_900011305	non-GC1	1550
GCF_900088705	non-GC1	1550
GCF_900093475	non-GC1	494
GCF_900117405	non-GC1	2
GCF_900117475	non-GC1	2
GCF_900117485	non-GC1	2
GCF_900117505	non-GC1	2
GCF_900117515	non-GC1	2
GCF_900117535	non-GC1	2
GCF_900117545	non-GC1	2
GCF_900117555	non-GC1	2
GCF_900117565	non-GC1	2
GCF_900117575	non-GC1	2
GCF_900117585	non-GC1	2
GCF_900117595	non-GC1	2
GCF_900117605	non-GC1	2
GCF_900117615	non-GC1	2
GCF_900117635	non-GC1	2
GCF_900117645	non-GC1	2
GCF_900117655	non-GC1	2
GCF_900117665	non-GC1	2
GCF_900117675	non-GC1	2
GCF_900117685	non-GC1	2

GCF_900117695	non-GC1	2
GCF_900117705	non-GC1	2
GCF_900117715	non-GC1	2
GCF_900117725	non-GC1	2
GCF_900117735	non-GC1	2
GCF_900117745	non-GC1	2
GCF_900117755	non-GC1	2
GCF_900117775	non-GC1	2
GCF_900117785	non-GC1	2
GCF_900117795	non-GC1	2
GCF_900117805	non-GC1	2
GCF_900117815	non-GC1	2
GCF_900117825	non-GC1	2
GCF_900117835	non-GC1	2
GCF_900117845	non-GC1	2
GCF_900117855	non-GC1	2
GCF_900117865	non-GC1	2
GCF_900117875	non-GC1	2
GCF_900117885	non-GC1	2
GCF_900117895	non-GC1	2
GCF_900117905	non-GC1	2
GCF_900117925	non-GC1	2
GCF_900117935	non-GC1	2
GCF_900117945	non-GC1	2
GCF_900117965	non-GC1	2
GCF_900117975	non-GC1	2
GCF_900117985	non-GC1	2
GCF_900117995	non-GC1	2
GCF_900118005	non-GC1	2
GCF_900118015	non-GC1	2
GCF_900118025	non-GC1	2
GCF_900118035	non-GC1	2
GCF_900118045	non-GC1	2
GCF_900118055	non-GC1	98
GCF_900118065	non-GC1	2
GCF_900118075	non-GC1	641
GCF_900118085	non-GC1	2
GCF_900118095	non-GC1	2
GCF_900118105	non-GC1	2
GCF_900118115	non-GC1	2
GCF_900118125	non-GC1	2
GCF_900118145	non-GC1	2
GCF_900118155	non-GC1	2
GCF_900119395	non-GC1	2
GCF_900157395	non-GC1	575
GCF_900157405	non-GC1	2

GCF_900161875	non-GC1	78
GCF_900161885	non-GC1	78
GCF_900161895	non-GC1	78
GCF_900161905	non-GC1	78
GCF_900161915	non-GC1	78
GCF_900161925	non-GC1	78
GCF_900161935	non-GC1	78
GCF_900161945	non-GC1	78
GCF_900161955	non-GC1	78
GCF_900161975	non-GC1	78
GCF_900161985	non-GC1	78
GCF_900161995	non-GC1	78
GCF_900162005	non-GC1	78
GCF_900162015	non-GC1	78
GCF_900169915	non-GC1	85
GCF_900175175	non-GC1	85
GCF_900175185	non-GC1	2
GCF_900175195	non-GC1	2
GCF_900176685	non-GC1	85
GCF_900176715	non-GC1	2
GCF_900243925	non-GC1	158
GCF_900406645	non-GC1	499
GCF_900406655	non-GC1	979
GCF_900406665	non-GC1	1233
GCF_900406675	non-GC1	1232
GCF_900406685	non-GC1	2
GCF_900406695	non-GC1	499
GCF_900406705	non-GC1	499
GCF_900406725	non-GC1	625
GCF_900406735	non-GC1	625
GCF_900406775	non-GC1	374
GCF_900444715	non-GC1	2
GCF_900444725	non-GC1	52
GCF_900444735	non-GC1	2
GCF_900444745	non-GC1	374
GCF_900444765	non-GC1	2
GCF_900444785	non-GC1	2
GCF_900444795	non-GC1	2
GCF_900444815	non-GC1	241
GCF_900476545	non-GC1	2
GCF_900476555	non-GC1	2
GCF_900476585	non-GC1	2
GCF_900476615	non-GC1	2
GCF_900476635	non-GC1	2
GCF_900476645	non-GC1	2
GCF_900476655	non-GC1	2

GCF_900476665	non-GC1	25
GCF_900476675	non-GC1	2
GCF_900476695	non-GC1	2
GCF_900476705	non-GC1	25
GCF_900476725	non-GC1	2
GCF_900476735	non-GC1	49
GCF_900476745	non-GC1	2
GCF_900476765	non-GC1	2
GCF_900476795	non-GC1	2
GCF_900476805	non-GC1	2
GCF_900476835	non-GC1	2
GCF_900476855	non-GC1	187
GCF_900476865	non-GC1	2
GCF_900476895	non-GC1	2
GCF_900476905	non-GC1	2
GCF_900476915	non-GC1	2
GCF_900476925	non-GC1	2
GCF_900476935	non-GC1	2
GCF_900476945	non-GC1	2
GCF_900476955	non-GC1	2
GCF_900476975	non-GC1	187
GCF_900476985	non-GC1	2
GCF_900476995	non-GC1	2
GCF_900477005	non-GC1	2
GCF_900477015	non-GC1	2
GCF_900477025	non-GC1	2
GCF_900477035	non-GC1	2
GCF_900477045	non-GC1	2
GCF_900477055	non-GC1	2
GCF_900477065	non-GC1	2
GCF_900477075	non-GC1	2
GCF_900477085	non-GC1	2
GCF_900477095	non-GC1	2
GCF_900477105	non-GC1	2
GCF_900477115	non-GC1	2
GCF_900477125	non-GC1	2
GCF_900477145	non-GC1	2
GCF_900477155	non-GC1	2
GCF_900477165	non-GC1	2
GCF_900477175	non-GC1	2
GCF_900477185	non-GC1	2
GCF_900477195	non-GC1	2
GCF_900477205	non-GC1	2
GCF_900477215	non-GC1	2
GCF_900477225	non-GC1	2
GCF_900477235	non-GC1	2

GCF_900477245	non-GC1	2
GCF_900477255	non-GC1	2
GCF_900477275	non-GC1	2
GCF_900477285	non-GC1	2
GCF_900477295	non-GC1	2
GCF_900477305	non-GC1	2
GCF_900477315	non-GC1	2
GCF_900477325	non-GC1	2
GCF_900477335	non-GC1	2
GCF_900477345	non-GC1	2
GCF_900477355	non-GC1	2
GCF_900477365	non-GC1	2
GCF_900477375	non-GC1	2
GCF_900477385	non-GC1	2
GCF_900477405	non-GC1	2
GCF_900477415	non-GC1	2
GCF_900477465	non-GC1	2
GCF_900477485	non-GC1	2
GCF_900477495	non-GC1	2
GCF_900477505	non-GC1	2
GCF_900477535	non-GC1	2
GCF_900477545	non-GC1	2
GCF_900477555	non-GC1	2
GCF_900477565	non-GC1	2
GCF_900477575	non-GC1	2
GCF_900477585	non-GC1	2
GCF_900477595	non-GC1	2
GCF_900477605	non-GC1	2
GCF_900477615	non-GC1	187
GCF_900477625	non-GC1	187
GCF_900477635	non-GC1	2
GCF_900477645	non-GC1	2
GCF_900477655	non-GC1	2
GCF_900477665	non-GC1	2
GCF_900477675	non-GC1	2
GCF_900477685	non-GC1	2
GCF_900477695	non-GC1	2
GCF_900477705	non-GC1	2
GCF_900477715	non-GC1	187
GCF_900477725	non-GC1	2
GCF_900477735	non-GC1	2
GCF_900477745	non-GC1	2
GCF_900477755	non-GC1	2
GCF_900477765	non-GC1	2
GCF_900477775	non-GC1	2
GCF_900477785	non-GC1	2

GCF_900477795	non-GC1	2
GCF_900477805	non-GC1	2
GCF_900477815	non-GC1	2
**GCF_900477825**	**non-GC1**	**2**
GCF_900477835	non-GC1	2
GCF_900477845	non-GC1	2
GCF_900477855	non-GC1	2
GCF_900477865	non-GC1	2
GCF_900477915	non-GC1	103
GCF_900477935	non-GC1	2
GCF_900477955	non-GC1	2
GCF_900477965	non-GC1	2
GCF_900478005	non-GC1	2
GCF_900478085	non-GC1	2
GCF_900489505	non-GC1	2
GCF_900490075	non-GC1	2
GCF_900490085	non-GC1	2
GCF_900490105	non-GC1	2
GCF_900493395	non-GC1	2
GCF_900493565	non-GC1	2
GCF_900493575	non-GC1	164
GCF_900493585	non-GC1	16
GCF_900493605	non-GC1	1432
GCF_900494395	non-GC1	2
GCF_900494405	non-GC1	2
GCF_900494415	non-GC1	2
GCF_900494425	non-GC1	2
GCF_900494435	non-GC1	1430
GCF_900494455	non-GC1	2
GCF_900494465	non-GC1	2
GCF_900494475	non-GC1	1430
GCF_900494495	non-GC1	2
GCF_900494515	non-GC1	2
GCF_900494555	non-GC1	2
GCF_900494645	non-GC1	215
GCF_900494665	non-GC1	2
GCF_900494695	non-GC1	2
GCF_900494705	non-GC1	1430
GCF_900494765	non-GC1	2
GCF_900494775	non-GC1	1432
GCF_900494795	non-GC1	10
GCF_900494805	non-GC1	2
GCF_900494815	non-GC1	16
GCF_900494825	non-GC1	2
GCF_900494835	non-GC1	2
GCF_900494845	non-GC1	2

GCF_900494875	non-GC1	129
GCF_900494885	non-GC1	2
GCF_900494895	non-GC1	2
GCF_900494905	non-GC1	2
GCF_900494915	non-GC1	2
GCF_900494925	non-GC1	2
GCF_900494935	non-GC1	164
GCF_900494945	non-GC1	2
GCF_900494955	non-GC1	578
GCF_900494965	non-GC1	2
GCF_900494975	non-GC1	25
GCF_900494985	non-GC1	2
GCF_900494995	non-GC1	2
GCF_900495005	non-GC1	1432
GCF_900495035	non-GC1	2
GCF_900495045	non-GC1	1356
GCF_900495055	non-GC1	2
GCF_900495065	non-GC1	2
GCF_900495075	non-GC1	2
GCF_900495085	non-GC1	2
GCF_900495095	non-GC1	2
GCF_900495115	non-GC1	2
GCF_900495125	non-GC1	2
GCF_900495145	non-GC1	2
GCF_900495155	non-GC1	2
GCF_900495165	non-GC1	2
GCF_900495175	non-GC1	2
GCF_900495185	non-GC1	2
GCF_900495205	non-GC1	52
GCF_900495215	non-GC1	2
GCF_900495225	non-GC1	823
GCF_900495235	non-GC1	1431
GCF_900495245	non-GC1	2
GCF_900495255	non-GC1	2
GCF_900495275	non-GC1	2
GCF_900495285	non-GC1	2
GCF_900495295	non-GC1	103
GCF_900495325	non-GC1	2
GCF_900495335	non-GC1	2
GCF_900495345	non-GC1	866
GCF_900495355	non-GC1	374
GCF_900495375	non-GC1	2
GCF_900495385	non-GC1	1153
GCF_900495395	non-GC1	2
GCF_900495405	non-GC1	203
GCF_900495415	non-GC1	653

GCF_900495425	non-GC1	2
GCF_900495445	non-GC1	215
GCF_900495455	non-GC1	2
GCF_900495465	non-GC1	2
GCF_900495485	non-GC1	2
GCF_900495495	non-GC1	164
GCF_900495505	non-GC1	2
GCF_900495515	non-GC1	2
GCF_900495525	non-GC1	2
GCF_900495545	non-GC1	1433
GCF_900495555	non-GC1	2
GCF_900495565	non-GC1	10
GCF_900495575	non-GC1	215
GCF_900495585	non-GC1	2
GCF_900495595	non-GC1	215
GCF_900495605	non-GC1	1057
GCF_900495615	non-GC1	2
GCF_900495635	non-GC1	32
GCF_900495645	non-GC1	193
GCF_900495655	non-GC1	2
GCF_900495665	non-GC1	2
GCF_900495685	non-GC1	2
GCF_900495695	non-GC1	2
GCF_900495705	non-GC1	2
GCF_900495715	non-GC1	2
GCF_900495725	non-GC1	2
GCF_900495735	non-GC1	25
GCF_900495755	non-GC1	2
GCF_900495775	non-GC1	2
GCF_900495785	non-GC1	2
GCF_900495795	non-GC1	164
GCF_900495805	non-GC1	2
GCF_900495815	non-GC1	164
GCF_900495825	non-GC1	2
GCF_900495835	non-GC1	25
GCF_900495845	non-GC1	2
GCF_900495855	non-GC1	2
GCF_900495865	non-GC1	164
GCF_900495875	non-GC1	2
GCF_900495885	non-GC1	2
GCF_900495895	non-GC1	2
GCF_900495905	non-GC1	2
GCF_900495915	non-GC1	2
GCF_900495925	non-GC1	2
GCF_900495965	non-GC1	372
GCF_900495975	non-GC1	939

GCF_900495985	non-GC1	164
GCF_900495995	non-GC1	2
GCF_900496005	non-GC1	2
GCF_900496015	non-GC1	164
GCF_900496025	non-GC1	2
GCF_900496045	non-GC1	578
GCF_900496055	non-GC1	2
GCF_900496075	non-GC1	2
GCF_900496085	non-GC1	2
GCF_900496095	non-GC1	25
GCF_900496125	non-GC1	164
GCF_900496135	non-GC1	16
GCF_900496145	non-GC1	164
GCF_900496155	non-GC1	129
GCF_900496165	non-GC1	164
GCF_900496175	non-GC1	129
GCF_900496185	non-GC1	16
GCF_900496195	non-GC1	215
GCF_900496225	non-GC1	2
GCF_900496245	non-GC1	16
GCF_900496255	non-GC1	2
GCF_900496265	non-GC1	2
GCF_900496285	non-GC1	2
GCF_900496295	non-GC1	16
GCF_900496305	non-GC1	2
GCF_900496315	non-GC1	2
GCF_900496325	non-GC1	25
GCF_900496335	non-GC1	164
GCF_900496345	non-GC1	2
GCF_900496355	non-GC1	2
GCF_900496365	non-GC1	129
GCF_900496375	non-GC1	2
GCF_900496385	non-GC1	2
GCF_900496395	non-GC1	16
GCF_900496405	non-GC1	164
GCF_900496415	non-GC1	374
GCF_900496425	non-GC1	16
GCF_900496435	non-GC1	164
GCF_900496445	non-GC1	2
GCF_900496455	non-GC1	108
GCF_900496465	non-GC1	374
GCF_900496475	non-GC1	129
GCF_900496485	non-GC1	113
GCF_900496495	non-GC1	164
GCF_900496505	non-GC1	164
GCF_900496525	non-GC1	16

GCF_900496535	non-GC1	215
GCF_900496545	non-GC1	16
GCF_900496555	non-GC1	193
GCF_900496565	non-GC1	2
GCF_900496595	non-GC1	215
GCF_900496605	non-GC1	129
GCF_900496615	non-GC1	374
GCF_900496625	non-GC1	215
GCF_900496635	non-GC1	2
GCF_900496645	non-GC1	215
GCF_900496655	non-GC1	2
GCF_900496665	non-GC1	215
GCF_900496695	non-GC1	2
GCF_900496705	non-GC1	215
GCF_900496745	non-GC1	25
GCF_900496755	non-GC1	2
GCF_900496775	non-GC1	2
GCF_900496785	non-GC1	2
GCF_900496795	non-GC1	2
GCF_900496835	non-GC1	2
GCF_900496845	non-GC1	2
GCF_900496855	non-GC1	2
GCF_900496865	non-GC1	215
GCF_900496875	non-GC1	2
GCF_900496905	non-GC1	2
GCF_900496915	non-GC1	2
GCF_900496935	non-GC1	215
GCF_900496945	non-GC1	215
GCF_900496955	non-GC1	2
GCF_900519195	non-GC1	2
GCF_901484905	non-GC1	636
GCF_901484985	non-GC1	2
GCF_901669785	non-GC1	2
GCF_901669965	non-GC1	2
GCF_902386065	non-GC1	499
GCF_902705255	non-GC1	1230
GCF_902705405	non-GC1	46
GCF_902705485	non-GC1	1596
GCF_902705505	non-GC1	1018
GCF_902728005	non-GC1	437
GCF_904884465	non-GC1	2
GCF_904884475	non-GC1	2
GCF_904884485	non-GC1	2
GCF_904884495	non-GC1	78
GCF_904884505	non-GC1	78
GCF_904884515	non-GC1	2

GCF_904884525	non-GC1	164
GCF_904884535	non-GC1	2
GCF_904884545	non-GC1	2
GCF_904884565	non-GC1	2
GCF_904884575	non-GC1	2
GCF_904884585	non-GC1	2
GCF_904884595	non-GC1	2
GCF_904884605	non-GC1	187
GCF_904884615	non-GC1	45
GCF_904884625	non-GC1	164
GCF_904884635	non-GC1	15
GCF_904884645	non-GC1	2
GCF_904884655	non-GC1	2
GCF_904885665	non-GC1	2
GCF_904885675	non-GC1	2
GCF_904885685	non-GC1	2
GCF_904885695	non-GC1	2
GCF_904885705	non-GC1	2
GCF_904885715	non-GC1	2
GCF_904885725	non-GC1	2
GCF_904885735	non-GC1	2
GCF_904885745	non-GC1	2
GCF_904885755	non-GC1	2
GCF_904885765	non-GC1	2
GCF_904885775	non-GC1	2
GCF_904885785	non-GC1	45
GCF_904885805	non-GC1	2
GCF_904885815	non-GC1	1142
GCF_904885825	non-GC1	2
GCF_904885845	non-GC1	570
GCF_904885855	non-GC1	2
GCF_904886845	non-GC1	2
GCF_904886865	non-GC1	2
GCF_904886875	non-GC1	2
GCF_904886885	non-GC1	2
GCF_904886915	non-GC1	2
GCF_904886925	non-GC1	2
GCF_904886935	non-GC1	2
GCF_904886945	non-GC1	2
GCF_904886955	non-GC1	604
GCF_904886965	non-GC1	2
GCF_904886975	non-GC1	2
GCF_904886985	non-GC1	2
GCF_904886995	non-GC1	2
GCF_904887005	non-GC1	2
GCF_904887035	non-GC1	2

GCF_904887515	non-GC1	2
GCF_904887525	non-GC1	400
GCF_904887535	non-GC1	400
GCF_904887555	non-GC1	2
GCF_904887565	non-GC1	400
GCF_904887575	non-GC1	2
GCF_904887585	non-GC1	2
GCF_904887595	non-GC1	499
GCF_904887605	non-GC1	2
GCF_904887625	non-GC1	2
GCF_904887635	non-GC1	78
GCF_904887645	non-GC1	2
GCF_904887655	non-GC1	2
GCF_904887665	non-GC1	15
GCF_904887675	non-GC1	78
GCF_904890265	non-GC1	571
GCF_904890275	non-GC1	2
GCF_904890285	non-GC1	2
