# Supplementary material for: Identification of a Specific Biomarker of Acinetobacter baumannii Global Clone 1 by Machine Learning and PCR Related to Metabolic Fitness of ESKAPE Pathogens
Source: mSystems. 2023 May 15;8(3):e00734-22. doi: 10.1128/msystems.00734-22 (PMC10308912; doi:10.1128/msystems.00734-22)
Supplement: TABLE S3 [file msystems.00734-22-s0005.pdf]

Table S3.

Unitig ID	Sequence	Length	Match Location	Gene Name	Gene Product
U1	TTATTCATAGCCTCCTGGATGCGGTAAATCACGGCAAAC ATCAAAAATATGGACTAATGCGGGTAAAATAGCGGCCAT TGATTCGCTTGCTCCACCACGGCTTCCCGGTAACGTCACT ACTAACGAACGGTCAATAAACCCTGCTACTCCGCGTGAC ATAGCAGCATATGGCGTGCGTTTTTGTCCAAATGAGCGA GCAGCTTCCATTAAGCCATCTAATTTACGTTCTAAAAGAG GCTCTAGCGTATCAACTGTAATGTCACGCTTACCAATTCC TGTCCCACCCACGGTCATAATGCAAGCATATGATTTGCTC AACTCAAGCACTAAGTTTTTCAAAGTATCCGCTTCATCTG GCAAAATTTGG	367	ABAYE1552: 558..924	*moaCB*	bifunctional protein [Includes: molybdenum cofactor biosynthesis protein C; molybdenum cofactor biosynthesis protein B]
U2	ACCGGCAAAAATTGCAACCCCAATAAAACCGTTCACCCA ACCGTTCATAAACTTTTGCATA	61	ABAYE1557: 1..61	-	putative permease (drug/metabolite transporter)
U3	ACGTAAGCCATATTGAGAGGCTGCAAGTTCGACACGAGC AACATCACCCAATCGGGTAACC	61	ABAYE1173: 777..837	-	putative Multidrug/solvent efflux pump membrane transporter
U4	AAACACTGGGAAATATACTTGAAAAATATCAACAACCCC GAACGTATGCGGGTTGCTCAGT	61	ABAYE3468: 844..904	-	conserved hypothetical protein; putative exported protein
U5	GTATATCTGGCACAAAGCAAAAGGCTTTCATGCCTTACA ACATCAACAAACCCGTAAA	58	ABAYE1549: 957..1014	*moaA*	molybdopterin biosynthesis, protein A
U6	CATAACCCCTAAAGTACGATGGCTTGATACGGCATCCCA CATTGTTAAGCTA	52	ABAYE1636: 975..1026	*cydB*	cytochrome d terminal oxidase polypeptide subunit II
U7	GAACTATGGCAATACCGATCAGTAAAGATAGAAGTACCT GCCAG	44	ABAYE2207: 192..235	-	putative permease
U8	TCCTTTAGAAAATTTGGTGGGACAAGAAGGCGAAGGTTA TA	41	ABAYE1412: 651..691	-	putative acyl-CoA dehydrogenase protein (acdB-like)
U9	TCATGTCGAGCTAAGCCAGTACCAGCAAAAGCTGAAG	37	ABAYE1194: 1095..1131	-	putative two-component system sensor histidine kinase

U10	CCCCCCGAACTACATCAATATGATCATATAAGGCC	35	ABAYE1494: 441..475	-	putative outer membrane porin, receptor for Fe(III)-coprogen, Fe(III)-ferrioxamine B and Fe(III)-rhodotrulic acid uptake (FhuE)
U11	TCCAGCACCAAATGTAAGACCTGTTGCCCCCC	32	ABAYE1494: 470..501	-	putative outer membrane porin, receptor for Fe(III)-coprogen, Fe(III)-ferrioxamine B and Fe(III)-rhodotrulic acid uptake (FhuE)
U12	TATTTTTCCACCGTGATTACGGAGTTTATCC	31	ABAYE2635: 1323..1353	*hcaG*	chlorogenate esterase
U13	TTCAACAGCACAAACCAATATTGGCTATACCCGTATTGTT GCGCCAACTGATGGTACGGTT	61	ABAYE3247: 522..582	-	putative secretion protein (HlyD family)
U14	GAAAATGCCTTAACACCACTAGGTGAGCAATTAGGTGCT GATTTA	45	ABAYE1991: 286..330	-	conserved hypothetical protein; putative exported protein
U15	AATAAAAGCTTGCTCACGTACAAATGAATTGGCGCCA	37	ABAYE2677: 501..537	-	putative FAD-dependent monooxygenase
U16	CTTCTTCACGGTCCATACCGATTAAATAAATATTTAAAGT AACTTCAATACTAAAACCTGT	61	ABAYE3144: 277..337	*ohr*	organic hydroperoxide resistance protein
U17	CCAGCGAATACCAAAATTGAAAAGAAGTCAATTAAGTTA ATTAAAGGAACAAAGTAAAGGT	61	ABAYE3148: 2300..2360	-	conserved hypothetical protein; putative membrane protein
U18	GGATATTTCCTAAAATATCTTCTACATCTATAGAATTACT ATTGTCTGAGTCGCCATAGTT	61	ABAYE3068: 2728..2788	-	putative outermembrane protein exposed to the bacterial surface
U19	AAATAGCATTCAAGAGTTAGCCGAAAAAATTGGGCTAGA TGCCAAAACCTTATGCCATACC	61	ABAYE3092: 969..1029	-	conserved hypothetical protein
U20	AATGTGACAAAACTCTACGTTTTCACAAACACCGCCACT GCCATTATCAAATATACCGTTA	61	ABAYE3022: 147..207	-	conserved hypothetical protein; putative exported protein
U21	ATTACATTTATTTAGCTTATCAATACCACTGTCTGTCAGT GTAATTAAAATACGGCGG	58	ABAYE2626: 288..345	*hcaR*	Repressor protein of the Hydroxycinnamate (hca) catabolic genes
U22	ACCATAAAGTGCCTGATAGAGATGCTCTAAAGTTAAACC ATG	42	ABAYE1461: 739..780	-	putative transcriptional regulator
U23	AGCCACTAAAACAGGGTTCATTGGCATGAGC	31	ABAYE2632: 384..414	*hcaE*	porin

U24	TGTTTTCTCAACTGTCACTAAAACTCGCCCCGGTTTTTTTATTGCGAGACCTTGATACCCC	61	ABAYE3069: 759..819	-	conserved hypothetical protein
U25	GGTTGCTTTTTTTGCCTTAATTTTCCCACTCGCAAAAGGGGCGCACTGGACTGCT	55	ABAYE3148: 1437..1491	-	conserved hypothetical protein; putative membrane protein
U26	AAAATGTCGGCTTAATTCCTGAAATAAAAAGGAGCTT	37	REGION:AYE(CU459141.1.1):1976606..1976642	-	-
U27	AGAATGTTTGTCTCAATTACCATTTGTTTGGCTTGT	36	ABAYE1194: 957..992	-	putative two-component system sensor histidine kinase
U28	TAATTTAAAGCCAGTTTCTTTAATTGCATTGTCT	34	ABAYE2167: 282..315	*metQ*	D-methionine transport protein (ABC superfamily, peri_bind)
U29	GCAAAAGCTGAAGATTTCTATAGAAGATACGGG	33	ABAYE1194: 1119..1151	-	putative two-component system sensor histidine kinase
U30	ATCAAGCTCCATGCCTTGATTCGTTTTAAACGC	33	ABAYE2673: 283..315	-	putative amino acid transport protein (LysE family)
U31	TATGTCATTTTTGCTTCGGCACTGGTTGGACTCG	34	ABAYE1932: 1300..1333	-	putative Chromate transporter
U32	ACGTAAAATCGTTCTAAAACCCACATGGGTA	31	ABAYE2816: 969..999	-	conserved hypothetical protein; putative exported protein
U33	AGTAATGTCTTGGCAAGTGAGTATGCTAATGTGCGCTGGCGCAGTCTCGCAGTGAG	56	ABAYE2622: 385..440	*vanK*	vanillate transporter (MFS superfamily)
U34	AACGGCGACCATATTGCGACGGCTATTCATGATTTTGAA	39	ABAYE0696: 121..159	-	putative protease
U35	TCCATCTGTCGGTTATATCATGAACTATGGCTTACAACAGGTCATGTCATCAGGTACAGGA	61	ABAYE1198: 1812..1872	*mrcB*	bifunctional protein [Includes penicillin-insensitive transglycosylase; penicillin-sensitive transpeptidase] (penicillin-binding protein 1B)

U36	GAGAGAACTACCATCTGGGCAGTCAGAGAAAAATTGACGTTCTTTACCTGCAATTGATTTATCAATAATTGGCTTATTATTAAT	84	ABAYE3308: 367..450	-	putative phosphatase; alkaline phosphatase
U37	CTCTTCGAAGTCCTGCATCAAAACGCCCTTGTACAATATCAACAAGACCATAATCAGATAT	61	ABAYE1813: 379..439	-	putative transcriptional regulator (LysR family)
U38	GCTGAGGAGTAATTTTTAGAGGACCCACCAGCCAAAAATTATCGACTTG	49	ABAYE2122: 481..529	*bla*$_{OXA\text{-}69}$	Carbapenem-hydrolyzing oxacillinase OXA-69 (Beta-lactamase OXA-69)
U39	GTCTCCACGCTTTGATTTTTAGATGATGATAATAATCTAAAATA	44	ABAYE0847: 6..49	-	putative Oxidoreductase, short chain dehydrogenase/reductase family
U40	AGACTAATAAATCTAAATAGCTCCTAAAAAACC	33	REGION:AYE(CU459141.1.1):2195471..2195503	-	-
U41	CAAAGTACTTCAATTCCTTATCGAGTAACACT	32	ABAYE2115: 22..53	-	conserved hypothetical protein; putative OsmC-like protein
U42	AACTGCTGCACTTTCTGTTTATAACAGTGGG	31	REGION:AB0057(CP001182.2):1246522..1246552	-	-
U43	AAAAGCGAAAACAGGTCGTACCCCGATTTTGGCGACTATTTTAGTTGGGGATGATGGTGCATCTGCAACTTATGTACGTATGAAAGGTAATGCCTGCCGCCGCGTAGGTATGGATTCATTAAAAATTGAATTACCACAAGAAACGACAACAGAACAATTATTAGCTGAAATCGAAAAGCTTAATGCCAATCCAGATGTTCAC	202	ABAYE0812: 75..276	*folD*	bifunctional protein [Includes: 5, 10-methylene-tetrahydrofolate dehydrogenase; 5, 10-methylene-tetrahydrofolate cyclohydrolase]
U44	ACGTCACTTCGATGCTAGCAAGTTCATTTGTAAAATGAAATAAATTTTCTGTTTGAATCAGTGCTTAAGCATTTCTG	77	REGION:AYE(CU459141.1.1):2661771..2661847	-	-

U45	AGTCCAAATGGCGTTTTGCATCGCCAAATAAAATCGCGCCT	41	ABAYE3223: 144..184	*dusA*	tRNA-dihydrouridine synthase A
U46	TGAGCCACATCGGTAATCGGATGACTTTTAGGAGAA	36	ABAYE2253: 438..473	-	putative amino acid transport protein (ABC superfamily, peri_bind)
U47	TTCCCCTGAAATAATCATTTTTCTTAGTTTAAG	33	ABAYE2625: 25..57	*vanR*	transcriptional regulator for ferulate or vanillate catabolism (GntR family)
U48	ATTACATGGCAGTTTACGTTTGATGCGTGAATGTCCGGAAGTTCAACATTTATATCAAAAC	61	ABAYE2962: 861..921	-	putative Lipase
U49	TTCAAAGTAAAGTACGCAGTCCACCATATGCTCTAAAACACGCGGACCTGCCAGTGCACCT	61	ABAYE0856: 666..726	*radA*	DNA repair protein
U50	TATTCATAACTCACTCGCTGTATTTAACCCCGCACCCGTGACCTAATA	48	REGION:AB0057(CP001182.2):1264958..1265005	-	-
U51	AATACACCAATACGGTTTTCATCAACATAAG	31	ABAYE1812: 302..332	-	conserved hypothetical protein; putative alpha/beta-hydrolase domain
U52	AACTGCTGCCGATTATCAACATAATATCCAT	31	ABAYE3021: 1359..1389	-	conserved hypothetical protein; putative large exoproteins involved in heme utilization or adhesion
U53	CCAGATGTCGAGCTGCGGATAAAGGTCAGTCCCTAAGCCTTCTACATGCACTAAAGTTTTG	61	ABAYE3426: 1209..1269	*ubiB*	2-octaprenylphenol hydroxylase of ubiquinone biosynthetic pathway
U54	CAAAATATCTAAACCAGCAACTATCTCAGGCTTTAAGCTAGAAGCATCGGCT	52	ABAYE3357: 342..393	*leuB*	3-isopropylmalate dehydrogenase
U55	TAAGCCACAAGCTTAGGCATGAAAAGGGAGCTAAATCATG	40	REGION:AYE(CU459141.1):1768118..1768157	-	-

U56	TTTAGATACACCTTTAGCAAAAATTGGTGGTAAAGGGTT GTTCGTTAAAGAATTGGAAGCA	61	ABAYE3508: 165..225	*hemC*	porphobilinogen deaminase (PBG) (Hydroxymethylbilane synthase) (HMBS) (Pre-uroporphyrinogen synthase)
U57	CCAACTAACTCAACACCTTGGTTTTTTAACCAGTT	35	ABAYE1693: 67..101	-	conserved hypothetical protein
U58	CAACTCACATAAAGAAAATAATCAATCACGAGTA	34	ABAYE1804: 351..384	-	hypothetical protein; putative Guanosine polyphosphate pyrophosphohydrolase/synthetase domain
U59	ACAACTCACTAAACCTTATTATTTTTTATTGAA	33	REGION:AYE(CU459141.1):2952946..2952978	-	-
U60	GAGGTTCCGACTGCAATATGCATGATTACCACGGGATCA TAGTGCAACTGGGTAAAAACGA	61	ABAYE3014: 101..161	-	conserved hypothetical protein; putative membrane protein
U61	AAAACCATACTCAGATGCGGTATTCATCACCGCTAAAAG TGA	42	ABAYE1910: 979..1020	-	putative D-beta-hydroxybutyrate permease
U62	TGTAGAAGATGGATTGAAAAAAATTCATCCATAATTCAT TTAATATTTAGAAAGTCTCCTC	61	REGION:AYE(CU459141.1.1):2561893..2561953	-	-
U63	AGTATGGCATGCCAACAGTTTAGGTAACTACTCATTCTTT GATAAGTCACAATCTGACTTT	61	ABAYE1718: 453..513	*catA*	catechol 1, 2-dioxygenase
U64	AAAATGCTTTAATTTCTGCTAAAGAAAAGCCAACTTG	37	ABAYE1734: 184..220	-	putative transcriptional regulator (MerR family)
U65	CATATTTGGCAAAGCTATTTGGACTGGAGTCTA	33	ABAYE1739: 262..294	-	putative transcriptional regulator (TetR family)
U66	CCCCGTACTGATTACCAGTTCAATGTCGGGA	31	ABAYE2245: 354..384	-	putative transcriptional regulator (LysR family)

U67	GAACTAGTAAGGCTGATATTCCGCCCCCTAGCATTGCCC CTACCGTATAGCCACAAGAAAT	61	ABAYE2465: 448..508	-	putative Permease of the major facilitator superfamily
U68	ACAAGCTAAGGCAGGTAAGGGCCAATTGTTGCCAAAAG GCATTAC	45	ABAYE1802: 630..674	-	putative Acid phosphatase
U69	CTCGTCACGCGACAAATCCATATTACGGCGACCAAAGCC T	40	ABAYE2441: 207..246	-	putative lactam utilization protein
U70	CCCAACACCAATATTGGTCTGGTTTAAACGC	31	ABAYE2439: 732..762	-	putative allophanate hydrolase subunit 1 and 2
U71	ACTAATAAGTAAGATTCAATAAGTACAGTGG	31	REGION:AY E(CU459141. 1):1875609..1 875639	-	-
U72	CAAAATGTACAGTCATGCCCGGTAAAAGTAGTGC	34	ABAYE2439: 568..601	-	putative allophanate hydrolase subunit 1 and 2
U73	TAGATTATGATAGTAAGGATGGAGTTATAGCATTAGTCT TTTATGAACCTGCACAAGTT	59	AB57_1176:1 49..207	-	hypothetical protein
U74	ATTAAAAACGAAAAATTGATTTTGTGACTCATTTCACAGT GCTGGCAA	48	REGION:AY E(CU459141. 1.1):3519890. .3519937	-	-
U75	TTTATCACCTAATTTTTCAATAGCGCTTGGTGATGGCCCT AC	42	ABAYE2438: 313..354	*bccA*	Acetyl-/propionyl-coenzyme A carboxylase alpha chain [Includes: Biotin carboxylase ; Biotin carboxyl carrier protein (BCCP)]
U76	TTCGTATCGGCACCAACCAATATGCACTTGGAGATTAAG CTTTTGATCTAGAGAGCTAATTTGCCAAAGCTCATCATTT ACTTTTTCAAATAAAACATCAGTCAGTGGAAATAAAACG CCGTTAATCCATAAGCAATTTTCATTACCAAAACTTTCAT TAACACCA	166	ABAYE0610: 690..855	-	conserved hypothetical protein
U77	ATATAATGTTGTACGTGAATGGTTGACGGTACAGATTCA AAACGCCCGTAGCGAGGCTTTCCATTTTCTTGAATTAAAT	82	ABAYE0610: 2..83	-	conserved hypothetical protein

	CCA				
U78	TTTGTCAAAAAAATAAAAATATCTTTTTTATTTCTAATTTAAACTTCATTATTT	54	REGION:AYE(CU459141.1.1):1873701..1873754	-	-
U79	TAAAAAAGATATTTTTATTTTTTTGACAAAATTCT	35	REGION:AYE(CU459141.1.1):1873725..1873759	-	-
U80	ATCAAGAATAAGCAAGTCATATTGATCTCTTTTTAGCATTTCCAAACCTTGAAGTCCTAAATGTACGCACTCGGCTTGGTATCCGGATTCTTTTAACCCTTTGACCAAATAAGTGGCGATTTTGATCTCGTCTTCAATAATAAGAATACGCATTTTGTAGAGACTCATTAGAAAAATGACAACTTTGTAATGTGATTGTCATGCT	205	REGION:AB0057(CP001182.2):2650869..2651073	-	-
U81	AACTCATTCTATTAACGTAAATCAGCTACAGAAACGGTTAAACCTCCCAAATTCACCTTTA	61	ABAYE1341:582..642	-	putative heavy metal sensor kinase
U82	TGTAAAATTGATGTTTTACCTGACCCAGAGACACCAAATAGCCCAATAACAGAAGCATCTG	61	ABAYE1892:65..125	*modC*	molybdate transport protein (ABC superfamily, atp_bind)
U83	GTCAACCATATTCCAACCTTAACTGGCGGTGTAGGCCAAAGC	42	REGION:AYE(CU459141.1):1958857..1958898	-	-
U84	TAAAATGCGAGCGCGGTTTTCTAAAGAACCCCCC	34	ABAYE1731:573..606	-	putative NADH-flavin oxidoreductase/NADH oxidase
U85	AAATACTTGATCATTTTCTACAAATAAATCGCGATTGGTGACCAGTGGCACATCCATTTCA	61	ABAYE1376:834..894	-	conserved hypothetical protein
U86	GGGAATCAAAAGCTGAAGCATTAAAAGAGCCGGAT	35	ABAYE1336:914..948	*pqiB*	paraquat-inducible protein
U87	GAGCCAAAACTTCCCCTAGACTTGAATAAACA	32	REGION:AB0057(CP0011	-	-

			82.2):1250433..1250464		
U88	CCGTCATGACTACTCCTAATTTAGTAGTGACGATGAGCTCCAGCAAATGCTGAAAAAGAGTCTAACTATGTGGCAATGACAGCAACAGATTTCAAAATGGAA	102	REGION:AYE(CU459141.1.1):1781435..1781536	-	-
U89	TTCTCTGTAGTAAGTAAGAATGTTTGAGTTACTGGATCTAACGTAAAGAAAGCTACACCGT	61	ABAYE0899:500..560	*fbp*	fructose-1, 6-bisphosphatase
U90	TCCAATCTCAAGAACTAAAAAATAGCGATAGAGAAAAAATAAGT	44	AB57_0738:275..318	-	aminodeoxychorismate synthase component I
U91	ATTGAAAATTGAAATAAAAACAGACTCATACAATTTAAAT	40	REGION:AYE(CU459141.1.1):1536664..1536703	-	-
U92	AAGAATTTCTGCAGTGCTGTCACTACTTCCATCATTTACAGCAATGACTTCAAAATTCGGA	61	ABAYE1396:222..282	-	Biofilm synthesis N-glycosyltransferase (PgaC-like)
U93	AAAAAAATAACCAATCAAAATACATAAAAAT	31	REGION:AYE(CU459141.1.1):2182767..2182797	-	-
U94	AGGTGTTACGGTTTATAGCTTTGCACAACTAGCAAATGCAGCAACGTTAAATGTAAAACCA	61	ABAYE2829:21..81	-	Aldose 1-epimerase precursor (Mutarotase)
U95	GCGGAATCAAAATTGCAGCAGCAGCTACAGTAGCACC	37	ABAYE1850:142..178	-	hypothetical protein; putative phage-related exported protein
U96	GAAATGGACATGGTCTGTAAGCAATTATCGTCACCTGATGCTAATGGGGTGCAGTCATGTCTTCAATGGG	70	ABAYE1851:40..109	-	hypothetical protein
U97	GAAAATGCTCCTTGTGTTGCACCGTATGTAAATAAAGAAAGTCCAGC	47	ABAYE1848:76..122	-	conserved hypothetical protein; putative Phage-related protein
U98	ATTAACTATGAGTTTAGCTAGTCTTCTTTCTAAAGTATCAGAAACAATT	49	REGION:AB0057(CP001182.2):	-	-

			2107318..2107366		
U99	TAAAAGCTGAAGTAATAGCACCAACAGCCAACACAGCAGGCTTAAAGAAA	50	ABAYE1849:1368..1417	-	hypothetical protein
U100	GTGATAAGCCATATATGCAGCAGCAGCTGCGGTAGTAGTCGAATAA	46	AB57_2066:510..555	-	hypothetical protein
