# Supplementary material for: Identification of a Specific Biomarker of Acinetobacter baumannii Global Clone 1 by Machine Learning and PCR Related to Metabolic Fitness of ESKAPE Pathogens
Source: mSystems. 2023 May 15;8(3):e00734-22. doi: 10.1128/msystems.00734-22 (PMC10308912; doi:10.1128/msystems.00734-22)
Supplement: TABLE S4 [file msystems.00734-22-s0006.pdf]

Table S4.

	Dataset 1		Dataset 2			
Unitig ID	Number of GC1 genomes matching the unitig	Number of non-GC1 genomes matching the unitig	Number of GC1 genomes matching the unitig	Number of non-GC1 genomes matching the unitig	P-value (proportion of GC1 genome matches in Dataset 1 not differs from proportion of GC1 matches in Dataset 2)	P-value (proportion of non-GC1 genome matches in Dataset 1 not differs from proportion of non-GC1 matches in Dataset 2)
U1	200/200	0/300	312/312	0/4487	p = 1	p = 1
U2	200/200	0/300	311/312	0/4487	p = 1	p = 1
U3	200/200	0/300	311/312	3/4487	p = 1	p = 1
U4	200/200	0/300	310/312	43/4487	p = 0.523	p = 0.1106
U5	200/200	0/300	311/312	0/4487	p = 1	p = 1
U6	200/200	0/300	306/312	1/4487	p = 0.0862	p = 1
U7	200/200	0/300	309/312	2/4487	p = 0.2845	p = 1
U8	200/200	0/300	312/312	1/4487	p = 1	p = 1
U9	200/200	0/300	309/312	5/4487	p = 0.2845	p = 1
U10	200/200	0/300	311/312	0/4487	p = 1	p = 1
U11	200/200	0/300	311/312	0/4487	p = 1	p = 1
U12	200/200	0/300	309/312	0/4487	p = 0.2845	p = 1
U13	200/200	1/300	306/312	46/4487	p = 0.162	p = 0.3653
U14	200/200	1/300	312/312	13/4487	p = 1	p = 0.5964
U15	200/200	1/300	312/312	3/4487	p = 1	p = 0.2281
U16	200/200	2/300	312/312	48/4487	p = 1	p = 0.7685
U17	200/200	2/300	312/312	50/4487	p = 1	p = 0.7707
U18	200/200	2/300	312/312	56/4487	p = 1	p = 0.5831
U19	200/200	2/300	306/312	51/4487	p = 0.162	p = 0.7722

U20	200/200	2/300	306/312	51/4487	p = 0.162	p = 0.7722
U21	200/200	2/300	310/312	20/4487	p = 0.523	p = 0.6453
U22	200/200	2/300	311/312	19/4487	p = 1	p = 0.3826
U23	200/200	2/300	310/312	25/4487	p = 0.523	p = 0.6852
U24	200/200	3/300	312/312	51/4487	p = 1	p = 1
U25	200/200	3/300	312/312	64/4487	p = 1	p = 0.7982
U26	200/200	3/300	311/312	45/4487	p = 1	p = 1
U27	200/200	4/300	309/312	26/4487	p = 0.2845	p = 0.115
U28	200/200	4/300	310/312	25/4487	p = 0.523	p = 0.1046
U29	200/200	4/300	309/312	26/4487	p = 0.2845	p = 0.115
U30	200/200	4/300	312/312	22/4487	p = 1	p = 0.07602
U31	200/200	6/300	312/312	90/4487	p = 1	p = 1
U32	200/200	6/300	309/312	55/4487	p = 0.2845	p = 0.2774
U33	200/200	7/300	310/312	129/4487	p = 0.523	p = 0.7203
U34	200/200	7/300	309/312	50/4487	p = 0.2845	p = 0.08792
U35	200/200	9/300	312/312	175/4487	p = 1	p = 0.535
U36	199/200	0/300	312/312	42/4487	p = 0.3906	p = 0.1101
U37	199/200	0/300	302/312	5/4487	p = 0.09716	p = 1
U38	199/200	0/300	310/312	0/4487	p = 1	p = 1
U39	199/200	0/300	309/312	0/4487	p = 1	p = 1
U40	199/200	0/300	310/312	11/4487	p = 1	p = 1
U41	199/200	0/300	311/312	27/4487	p = 1	p = 0.411
U42	199/200	0/300	308/312	0/4487	p = 0.653	p = 1
U43	199/200	1/300	309/312	3/4487	p = 1	p = 0.2281
U44	199/200	1/300	307/312	1/4487	p = 0.4118	p = 0.1214
U45	199/200	1/300	312/312	53/4487	p = 1	p = 0.2581
U46	199/200	1/300	303/312	12/4487	p = 0.09715	p = 0.5693
U47	199/200	1/300	309/312	15/4487	p = 1	p = 1
U48	199/200	2/300	307/312	51/4487	p = 0.4118	p = 0.7722
U49	199/200	2/300	308/312	9/4487	p = 0.653	p = 0.5831

U50	199/200	2/300	309/312	5/4487	p = 1	p = 0.06666
U51	199/200	3/300	302/312	44/4487	p = 0.09716	p = 1
U52	199/200	3/300	307/312	57/4487	p = 0.4118	p = 1
U53	198/200	0/300	304/312	51/4487	p = 0.329	p = 0.07431
U54	198/200	0/300	304/312	42/4487	p = 0.329	p = 0.1101
U55	198/200	0/300	303/312	0/4487	p = 0.2152	p = 1
U56	198/200	1/300	299/312	46/4487	p = 0.05668	p = 0.3653
U57	198/200	1/300	300/312	2/4487	p = 0.09179	p = 0.1765
U58	198/200	1/300	299/312	30/4487	p = 0.05668	p = 0.7195
U59	198/200	13/300	310/312	240/4487	p = 0.6457	p = 0.5072
U60	198/200	2/300	305/312	56/4487	p = 0.4932	p = 0.5831
U61	198/200	4/300	310/312	34/4487	p = 0.6457	p = 0.09512
U62	197/200	0/300	307/312	8/4487	p = 1	p = 1
U63	197/200	0/300	301/312	0/4487	p = 0.2663	p = 1
U64	197/200	0/300	299/312	2/4487	p = 0.119	p = 1
U65	197/200	0/300	296/312	12/4487	p = 0.05226	p = 1
U66	197/200	0/300	299/312	0/4487	p = 0.119	p = 1
U67	197/200	1/300	309/312	2/4487	p = 0.6828	p = 0.1765
U68	197/200	1/300	296/312	5/4487	p = 0.05226	p = 0.3219
U69	197/200	3/300	307/312	5/4487	p = 1	p = 0.01077
U70	197/200	3/300	307/312	11/4487	p = 1	p = 0.05298
U71	197/200	3/300	296/312	46/4487	p = 0.05226	p = 1
U72	197/200	9/300	307/312	67/4487	p = 1	p = 0.05354
U73	196/200	0/300	306/312	0/4487	p = 0.7731	p = 1
U74	196/200	0/300	305/312	43/4487	p = 1	p = 0.1106
U75	196/200	0/300	306/312	0/4487	p = 0.7731	p = 1
U76	195/200	0/300	292/312	48/4487	p = 0.05736	p = 0.07245
U77	195/200	0/300	292/312	48/4487	p = 0.05736	p = 0.07245
U78	194/200	0/300	290/312	5/4487	p = 0.07112	p = 1
U79	194/200	0/300	290/312	5/4487	p = 0.07112	p = 1

U80	193/200	0/300	301/312	2/4487	p = 1	p = 1
U81	193/200	3/300	301/312	11/4487	p = 1	p = 0.0534
U82	192/200	0/300	298/312	3/4487	p = 1	p = 1
U83	192/200	0/300	303/312	5/4487	p = 0.6143	p = 1
U84	192/200	0/300	287/312	3/4487	p = 0.09572	p = 1
U85	191/200	0/300	307/312	0/4487	p = 0.0568	p = 1
U86	191/200	0/300	291/312	1/4487	p = 0.3392	p = 1
U87	190/200	0/300	295/312	3/4487	p = 1	p = 1
U88	189/200	0/300	283/312	0/4487	p = 0.1312	p = 1
U89	185/200	0/300	283/312	1/4487	p = 0.5216	p = 1
U90	185/200	3/300	290/312	51/4487	p = 0.8624	p = 1
U91	184/200	1/300	290/312	3/4487	p = 0.731	p = 0.2281
U92	183/200	0/300	297/312	0/4487	p = 0.0962	p = 1
U93	181/200	6/300	287/312	142/4487	p = 0.6284	p = 0.3046
U94	178/200	2/300	283/312	11/4487	p = 0.5476	p = 0.194
U95	169/200	4/300	252/312	24/4487	p = 0.2896	p = 0.09456
U96	168/200	4/300	251/312	26/4487	p = 0.348	p = 0.115
U97	165/200	4/300	245/312	19/4487	p = 0.3079	p = 0.05257
U98	160/200	2/300	228/312	21/4487	p = 0.09049	p = 0.6524
U99	158/200	4/300	243/312	23/4487	p = 0.8262	p = 0.08504
U100	151/200	0/300	247/312	2/4487	p = 0.3852	p = 1
