# Supplementary material for: Identification of a Specific Biomarker of Acinetobacter baumannii Global Clone 1 by Machine Learning and PCR Related to Metabolic Fitness of ESKAPE Pathogens
Source: mSystems. 2023 May 15;8(3):e00734-22. doi: 10.1128/msystems.00734-22 (PMC10308912; doi:10.1128/msystems.00734-22)
Supplement: TABLE S5 [file msystems.00734-22-s0007.pdf]

Table S5.

Rule ID	Rule	Length	Match Location	Gene Name	Gene Product
R127	Presence(AAAAAAGCATGTTTGAAACATGCTTTTTTATTTTATGGCGTTAAACCAACAGGATTGCGATACCAGCTCTGAATTAGCAAAGCCGCGGCAAAACTATCGGCTGACAACTTCTTGGCACGGCCTTGTT)	127	AYE(CU459141.1):REGION: 3509175..3509301	-	putative Holliday junction resolvase
R125	Absence(AATGATTAACAGTACAGGGAAACTAGCAATGAGAAGTTGCATCAAAATGCCTTGACGTTGTGGTGCTGTGCCCTCTACAACAACATTTTGCTTGTTTAAGCTTGGCATAAGTTCAGTGTCTTCAA)	125	ACICU_02924:125..249	-	ATP-dependent_Zn_protease
R123	Absence(AATGATTAACAGTACAGGGAAACTAGCAATGAGAAGTTGCATCAAAATGCCTTGACGTTGTGGTGCTGTGCCCTCTACAACAACATTTTGCTTGTTTAAGCTTGGCATAAGTTCAGTGTCTT	123	ACICU_02924:127..249	-	ATP-dependent_Zn_protease

	C)				
R121	Absence(AATGATTAACAGTACAGGGAAACTAGCAATGAGAAGTTGCATCAAAATGCCTTGACGTTGTGGTGCTGTGCCCTCTACAACAACATTTTGCTTGTTTAAGCTTGGCATAAGTTCAGTGTCT)	121	ACICU_02924:129..249	-	ATP-dependent_Zn_protease
R119	Absence(AATGATTAACAGTACAGGGAAACTAGCAATGAGAAGTTGCATCAAAATGCCTTGACGTTGTGGTGCTGTGCCCTCTACAACAACATTTTGCTTGTTTAAGCTTGGCATAAGTTCAGTGT)	119	ACICU_02924:131..249	-	ATP-dependent_Zn_protease

R117	Absence(AACAGTACAGGGAAACTAGCAATGAGAAGTTGCATCAAAATGCCTTGACGTTGTGGTGCTGTGCCCTCTACAACAACATTTTGCTTGTTTAAGCTTGGCATAAGTTCAGTGTCTTCA)	117	ACICU_02924:126..242	-	ATP-dependent_Zn_protease
R115	Absence(AACAGTACAGGGAAACTAGCAATGAGAAGTTGCATCAAAATGCCTTGACGTTGTGGTGCTGTGCCCTCTACAACAACATTTTGCTTGTTTAAGCTTGGCATAAGTTCAGTGTCTT)	115	ACICU_02924:128..242	-	ATP-dependent_Zn_protease
R113	Absence(AACAGTACAGGGAAACTAGCAATGAGAAGTTGCATCAAAATGCCTTGACGTTGTGGTGCTGTGCCCTCTACAACAACATTTTGCTTGTTTAAGCTTGGCATAAGTTCAGTGTC)	113	ACICU_02924:130..242	-	ATP-dependent_Zn_protease

R111	Absence(AAGACACTGAACTTATGCCAAGCTTAAACAAGCAAAATGTTGTTGTAGAGGGCACAGCACCACAACGTCAAGGCATTTTGATGCAACTTCTCATTGCTAGTTTCCCTGTAC)	111	ACICU_02924:128..238	-	ATP-dependent_Zn_protease
R109	Absence(AAGACACTGAACTTATGCCAAGCTTAAACAAGCAAAATGTTGTTGTAGAGGGCACAGCACCACAACGTCAAGGCATTTTGATGCAACTTCTCATTGCTAGTTTCCCTGT)	109	ACICU_02924:128..236	-	ATP-dependent_Zn_protease
R107	Absence(AACAGTACAGGGAAACTAGCAATGAGAAGTTGCATCAAAATGCCTTGACGTTGTGGTGCTGTGCCCTCTACAACAACATTTTGCTTGTTTAAGCTTGGCATAAGTTC)	107	ACICU_02924:136..242	-	ATP-dependent_Zn_protease
R105	Absence(AACAGTACAGGGAAACTAGCAATGAGAAGTTGCATCAAAATGCCTTGACGTTGTGGTGCTGTGCCCTCTACAACAACATTTTGCTTGTTTAAG	105	ACICU_02924:138..242	-	ATP-dependent_Zn_protease

	CTTGGCATAAGT)				
R103	Absence(AACAGTACAGGGAAACTAGCAATGAGAAGTTGCATCAAAATGCCTTGACGTTGTGGTGCTGTGCCCTCTACAACAACATTTTGCTTGTTTAAGCTTGGCATAA)	103	ACICU_02924:140..242	-	ATP-dependent_Zn_protease
R101	Absence(AAACTAGCAATGAGAAGTTGCATCAAAATGCCTTGACGTTGTGGTGCTGTGCCCTCTACAACAACATTTTGCTTGTTTAAGCTTGGCATAAGTTCAGTGTC)	101	ACICU_02924:130..230	-	ATP-dependent_Zn_protease
R99	Absence(AAACTAGCAATGAGAAGTTGCATCAAAATGCCTTGACGTTGTGGTGCTGTGCCCTCTACAACAACATTTTGCTTGTTTAAGCTTGGCATAAGTTCAGTG)	99	ACICU_02924:132..230	-	ATP-dependent_Zn_protease

R97	Absence(AATGAGAAGTTGCATCAAAATGCCTTGACGTTGTGGTGCTGTGCCCTCTACAACAACATTTTGCTTGTTTAAGCTTGGCATAAGTTCAGTGTCTTCA)	97	ACICU_02924:126..222	-	ATP-dependent_Zn_protease
R95	Absence(AAACAAGCAAAATGTTGTTGTAGAGGGCACAGCACCACAACGTCAAGGCATTTTGATGCAACTTCTCATTGCTAGTTTCCCTGTACTGTTAATCA)	95	ACICU_02924:153..247	-	ATP-dependent_Zn_protease
R93	Absence(AAGATCATTATTTATACACGGGTATTTAGTTACAAAATAATGGGCATCACGGAGAAACACCCCGCGCTCATTAACTTTTGGCTTGAACTCTAG)	93	ACICU(CP000863.1) REGION: 2455717..2455809	-	-
R91	Absence(ATTAACTTATACCATTCATACCCCGCAACTCCTGCTGCTACAATCATAAGTACCAACATTGGATAATGTGATTGCGTAGCAAACATACAGC)	91	ACICU_02095:50..140	-	CDP-diglyceride_synthetase

R89	Absence(AACTTATGCCAAGCTTAAACAAGCAAAATGTTGTTGTAGAGGGCACAGCACCACAACGTCAAGGCATTTTGATGCAACTTCTCATTGCT)	89	ACICU_02924:137..225	-	ATP-dependent_Zn_protease
R87	Absence(AACTTATACCATTCATACCCCGCAACTCCTGCTGCTACAATCATAAGTACCAACATTGGATAATGTGATTGCGTAGCAAACATACAG)	87	ACICU_02095:51..137	-	CDP-diglyceride_synthetase
R85	Absence(ACTTATACCATTCATACCCCGCAACTCCTGCTGCTACAATCATAAGTACCAACATTGGATAATGTGATTGCGTAGCAAACATACA)	85	ACICU_02095:52..136	-	CDP-diglyceride_synthetase
R83	Absence(AAACAAGCAAAATGTTGTTGTAGAGGGCACAGCACCACAACGTCAAGGCATTTTGATGCAACTTCTCATTGCTAGTTTCCCTG)	83	ACICU_02924:153..235	-	ATP-dependent_Zn_protease

R81	Absence(AAAATGCCTTGACGTTGTGGTGCTGTGCCCTCTACAACAACATTTTGCTTGTTTAAGCTTGGCATAAGTTCAGTGTCTTCA)	81	ACICU_02924:126..206	-	ATP-dependent_Zn_protease
R79	Absence(AAAATGCCTTGACGTTGTGGTGCTGTGCCCTCTACAACAACATTTTGCTTGTTTAAGCTTGGCATAAGTTCAGTGTCTT)	79	ACICU_02924:128..206	-	ATP-dependent_Zn_protease
R77	Absence(AAAATGCCTTGACGTTGTGGTGCTGTGCCCTCTACAACAACATTTTGCTTGTTTAAGCTTGGCATAAGTTCAGTGTC)	77	ACICU_02924:130..206	-	ATP-dependent_Zn_protease
R75	Absence(AACTTATACCATTCATACCCCGCAACTCCTGCTGCTACAATCATAAGTACCAACATTGGATAATGTGATTGCGTA)	75	ACICU_02095:63..137	-	CDP-diglyceride_synthetase
R73	Absence(ACAATGGCATCAACTTTTTTAAGCACACGTGTTCCCATCGGCAGCCATGTATATAAACCAGATGCCAATTTAC)	73	ACICU_03067:98..170	-	Prolyl-tRNA_synthetase

R71	Absence(AAATGTTTTTTGAGTAACCAAGAAATAGTTAAAAATAAAGAAATATACAGTGTTGGGGTTTACCCCTAGGC)	71	ACICU(CP000863.1) REGION: 1676569..1676639	-	-
R69	Absence(AAAATGCCTTGACGTTGTGGTGCTGTGCCCTCTACAACAACATTTTGCTTGTTTAAGCTTGGCATAAGT)	69	ACICU_02924:138..206	-	ATP-dependent_Zn_protease
R67	Presence(AAAACTATCGGCTGACAACTTCTTGGCACGGCCTTGTTCTTGATAAAAACCAAGTTCTTCTCTTGCC)	67	ABAYE3455:318..384	-	putative Holliday junction resolvase
R65	Presence(ACAACTTCTTGGCACGGCCTTGTTCTTGATAAAAACCAAGTTCTTCTCTTGCCTCACGAGTGGTG)	65	ABAYE3455:306..370	-	putative Holliday junction resolvase
R63	Absence(AAAATGCCTTGACGTTGTGGTGCTGTGCCCTCTACAACAACATTTTGCTTGTTTAAGCTTGGC)	63	ACICU_02924:144..206	-	ATP-dependent_Zn_protease
R61	Absence(AAAATAATGGGCATCACGGAGAAACACCCCGCGCTCATTAACTTTTGGCTTGAACTCTAGT)	61	ACICU(CP000863.1) REGION: 2455716..2455776	-	-

R59	Absence(AAAATAATGGGCATCACGGAGAAACACCCCGCGCTCATTAACTTTTGGCTTGAACTCTA)	59	ACICU(CP000863.1) REGION: 2455718..2455776	-	-
R57	Absence(AAAAGTTATTTATACCGATATGCAAAAGTGAATCCAGCTATGTCTAAAAATACTGCT)	57	ACICU_01506:142..198	-	Aspartate/tyrosine/aromatic_aminotransferase
R55	Absence(AACTTATACCATTCATACCCCGCAACTCCTGCTGCTACAATCATAAGTACCAACA)	55	ACICU_02095:83..137	-	CDP-diglyceride_synthetase
R53	Absence(AAAATAATGGGCATCACGGAGAAACACCCCGCGCTCATTAACTTTTGGCTTGA)	53	ACICU(CP000863.1) REGION: 2455724..2455776	-	-
R51	Presence(AACTTGGTTTTTATCAAGAACAAGGCCGTGCCAAGAAGTTGTCAGCCGATA)	51	ABAYE3455:329..379	-	putative Holliday junction resolvase
R49	Absence(AAAAGTTATTTATACCGATATGCAAAAGTGAATCCAGCTATGTCTAAAA)	49	ACICU_01506:142..190	-	Aspartate/tyrosine/aromatic_aminotransferase
R47	Presence(AAAGAAGGAAGCCATAACACCTTGCTTTAAACCAAAGTCCTGATCAG)	47	ABAYE2468:605..651	-	putative lipid A biosynthesis acyltransferase

R45	Presence(AAAGAAGGAAGCCATAACACCTTGCTTTAAACCAAAGTCCTGATC)	45	ABAYE2468:607..651	-	putative lipid A biosynthesis acyltransferase
R43	Absence(AAAAATGCATCTTAAAACATTAAGCTTCGGTTTAGCAGTAGCA)	43	ACICU(CP000863.1) REGION: 1679383..1679425	-	-
R41	Presence(AAAACTATCGGCTGACAACTTCTTGGCACGGCCTTGTTCTT)	41	ABAYE3455:344..384	-	putative Holliday junction resolvase
R39	Absence(AAAAATAAAGAAATATACAGTGTTGGGGTTTACCCCTAG)	39	ACICU(CP000863.1) REGION: 1676599..1676637	-	-
R37	Presence(AAGAACAAGGCCGTGCCAAGAAGTTGTCAGCCGATAG)	37	ABAYE3455:344..380	-	putative Holliday junction resolvase
R35	Absence(AAAAATGCATCTTAAAACATTAAGCTTCGGTTTAG)	35	ACICU(CP000863.1) REGION: 2455725..2455776	-	-
R33	Presence(ACCCGTTTTATTCCGGGTATTGCGTTTAACTAT)	33	ABAYE2213:1093..1125	-	conserved hypothetical protein; putative exported protein
R31	Presence(TACCAATACGACCGGCCGCTAAATTAGATAA)	31	ABAYE1412:700..730	-	putative acyl-CoA dehydrogenase protein (acdB-like)
