# Supplementary material for: Identification of a Specific Biomarker of Acinetobacter baumannii Global Clone 1 by Machine Learning and PCR Related to Metabolic Fitness of ESKAPE Pathogens
Source: mSystems. 2023 May 15;8(3):e00734-22. doi: 10.1128/msystems.00734-22 (PMC10308912; doi:10.1128/msystems.00734-22)
Supplement: TABLE S6 [file msystems.00734-22-s0008.pdf]

Table S6.

	Dataset 1		Dataset 2			
Rule ID	Number of GC1 genomes matching the rule	Number of non-GC1 genomes matching the rule	Number of GC1 genomes matching the rule	Number of non-GC1 genomes matching the rule	P-value (proportion of GC1 genome matches in Dataset 1 not differs from proportion of GC1 matches in Dataset 2)	P-value (proportion of non-GC1 genome matches in Dataset 1 not differs from proportion of non-GC1 matches in Dataset 2)
R127	200/200	4/300	310/312	166/4487	p = 0.523	p = 0.03405
R125	200/200	1/300	310/312	13/4487	p = 0.523	p = 0.5964
R123	200/200	1/300	310/312	13/4487	p = 0.523	p = 0.5964
R121	200/200	1/300	310/312	13/4487	p = 0.523	p = 0.5964
R119	200/200	1/300	310/312	13/4487	p = 0.523	p = 0.5964
R117	200/200	1/300	310/312	12/4487	p = 0.523	p = 0.5693
R115	200/200	1/300	310/312	12/4487	p = 0.523	p = 0.5693
R113	200/200	1/300	310/312	12/4487	p = 0.523	p = 0.5693
R111	200/200	1/300	310/312	12/4487	p = 0.523	p = 0.5693
R109	200/200	1/300	310/312	12/4487	p = 0.523	p = 0.5693
R107	200/200	1/300	310/312	12/4487	p = 0.523	p = 0.5693
R105	200/200	1/300	310/312	12/4487	p = 0.523	p = 0.5693
R103	200/200	1/300	310/312	12/4487	p = 0.523	p = 0.5693
R101	200/200	1/300	310/312	12/4487	p = 0.523	p = 0.5693
R99	200/200	1/300	310/312	12/4487	p = 0.523	p = 0.5693
R97	200/200	1/300	310/312	12/4487	p = 0.523	p = 0.5693
R95	200/200	1/300	310/312	13/4487	p = 0.523	p = 0.5964

R93	200/200	3/300	312/312	66/4487	p = 1	p = 0.8001
R91	200/200	3/300	312/312	28/4487	p = 1	p = 0.4407
R89	200/200	1/300	310/312	12/4487	p = 0.523	p = 0.5693
R87	200/200	3/300	312/312	28/4487	p = 1	p = 0.4407
R85	200/200	3/300	312/312	28/4487	p = 1	p = 0.4407
R83	200/200	1/300	310/312	12/4487	p = 0.523	p = 0.5693
R81	200/200	1/300	310/312	12/4487	p = 0.523	p = 0.5693
R79	200/200	1/300	310/312	12/4487	p = 0.523	p = 0.5693
R77	200/200	1/300	310/312	12/4487	p = 0.523	p = 0.5693
R75	200/200	3/300	312/312	28/4487	p = 1	p = 0.4407
R73	200/200	1/300	309/312	107/4487	p = 0.2845	p = 0.01413
R71	199/200	2/300	310/312	44/4487	p = 1	p = 1
R69	200/200	1/300	310/312	12/4487	p = 0.523	p = 0.5693
R67	200/200	3/300	310/312	121/4487	p = 0.523	p = 0.088
R65	200/200	3/300	310/312	121/4487	p = 0.523	p = 0.088
R63	200/200	1/300	310/312	12/4487	p = 0.523	p = 0.5693
R61	200/200	2/300	312/312	26/4487	p = 1	p = 0.6941
R59	200/200	2/300	312/312	26/4487	p = 1	p = 0.6941
R57	200/200	2/300	312/312	14/4487	p = 1	p = 0.2651
R55	200/200	2/300	312/312	27/4487	p = 1	p = 0.703
R53	200/200	2/300	312/312	26/4487	p = 1	p = 0.6941
R51	200/200	3/300	311/312	121/4487	p = 1	p = 0.088
R49	200/200	1/300	312/312	13/4487	p = 1	p = 0.5964
R47	198/200	4/300	308/312	65/4487	p = 1	p = 1
R45	198/200	4/300	309/312	65/4487	p = 1	p = 1
R43	199/200	2/300	311/312	62/4487	p = 1	p = 0.4354
R41	200/200	4/300	311/312	180/4487	p = 1	p = 0.01842
R39	199/200	1/300	310/312	24/4487	p = 1	p =1
R37	200/200	4/300	311/312	180/4487	p = 1	p = 0.01842

R35	199/200	1/300	311/312	31/4487	p = 1	p = 0.7196
R33	200/200	4/300	305/312	40/4487	p = 0.04632	p = 0.3537
R31	200/200	2/300	312/312	42/4487	p = 1	p = 0.703
