# Supplementary figures and images for: Identification of a Specific Biomarker of Acinetobacter baumannii Global Clone 1 by Machine Learning and PCR Related to Metabolic Fitness of ESKAPE Pathogens
Source: mSystems. 2023 May 15;8(3):e00734-22. doi: 10.1128/msystems.00734-22 (PMC10308912; doi:10.1128/msystems.00734-22)

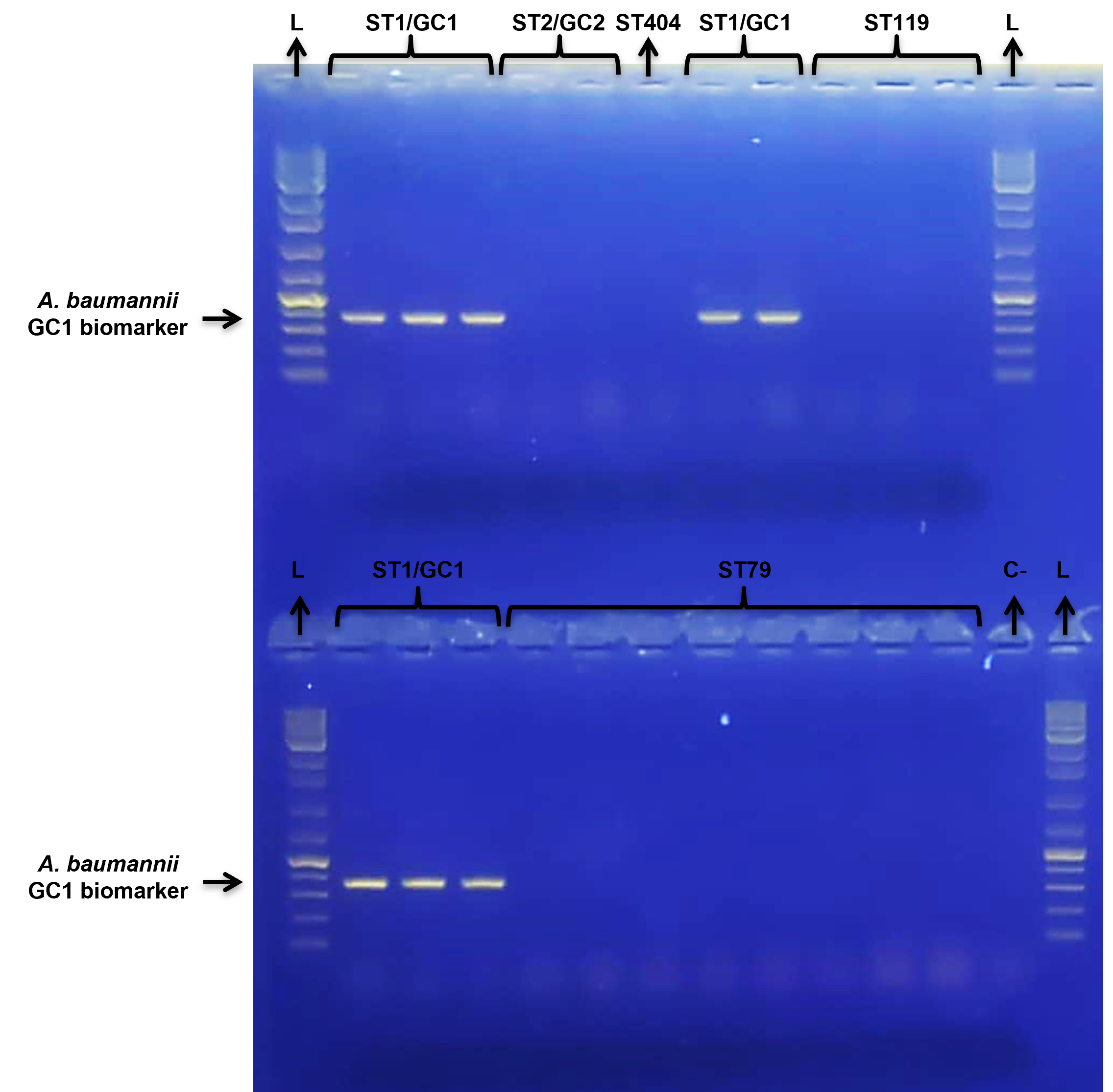

Supplement: FIG S1 [file msystems.00734-22-s0001.tif]

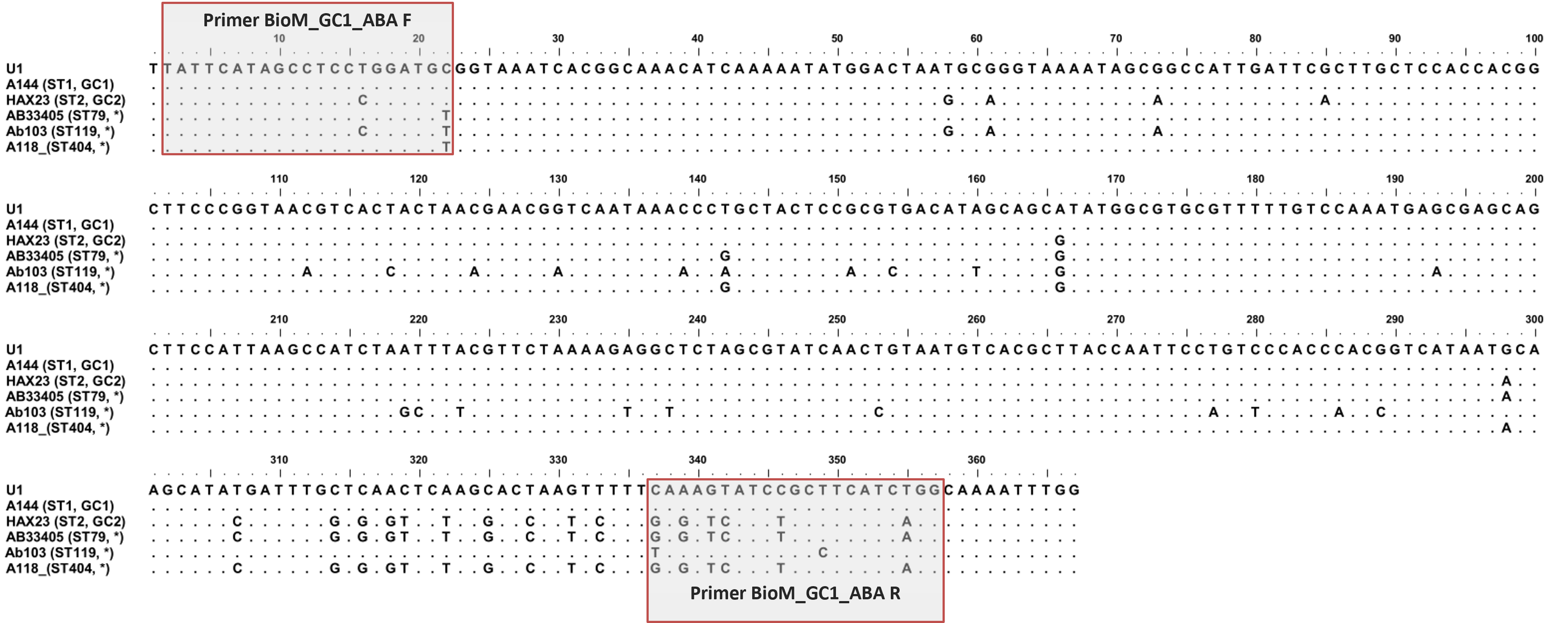

Supplement: FIG S2 [file msystems.00734-22-s0002.tif]
