# Supplementary material for: Identification of a Specific Biomarker of Acinetobacter baumannii Global Clone 1 by Machine Learning and PCR Related to Metabolic Fitness of ESKAPE Pathogens
Source: mSystems. 2023 May 15;8(3):e00734-22. doi: 10.1128/msystems.00734-22 (PMC10308912; doi:10.1128/msystems.00734-22)
Supplement: TABLE S7 [file msystems.00734-22-s0009.pdf]

Table S7.

										Number of isolates typified by MLST		
**Rule ID**	**Sensitivity**	**Specificity**	**Precision**	**Accuracy**	**F1-score**	**True Positives**	**True Negatives**	**False Positives**	**False Negatives**	**GC1**	**non-GC1**	**Correlation of MLST typing to the model prediction**
R31	1.00	0.98	0.97	0.99	0.99	72	93	2	0	72	95	$p < 2.2e^{-16}$
R33	1.00	0.96	0.95	0.98	0.97	72	91	4	0	72	95	$p < 2.2e^{-16}$
R35	0.99	0.99	0.99	0.99	0.99	71	94	1	1	72	95	$p < 2.2e^{-16}$
R37	1.00	0.96	0.95	0.98	0.97	72	91	4	0	72	95	$p < 2.2e^{-16}$
R39	0.99	0.99	0.99	0.99	0.99	71	94	1	1	72	95	$p < 2.2e^{-16}$
R41	1.00	0.96	0.95	0.98	0.97	72	91	4	0	72	95	$p < 2.2e^{-16}$
R43	0.99	0.98	0.97	0.98	0.98	71	93	2	1	72	95	$p < 2.2e^{-16}$
R45	0.97	0.96	0.95	0.96	0.96	70	91	4	2	72	95	$p < 2.2e^{-16}$
R47	0.97	0.96	0.95	0.96	0.96	70	91	4	2	72	95	$p < 2.2e^{-16}$
R49	1.00	0.99	0.99	0.99	0.99	72	94	1	0	72	95	$p < 2.2e^{-16}$
R51	1.00	0.97	0.96	0.98	0.98	72	92	3	0	72	95	$p < 2.2e^{-16}$
R53	1.00	0.98	0.97	0.99	0.99	72	93	2	0	72	95	$p < 2.2e^{-16}$
R55	1.00	0.98	0.97	0.99	0.99	72	93	2	0	72	95	$p < 2.2e^{-16}$
R57	1.00	0.98	0.97	0.99	0.99	72	93	2	0	72	95	$p < 2.2e^{-16}$
R59	1.00	0.98	0.97	0.99	0.99	72	93	2	0	72	95	$p < 2.2e^{-16}$
R61	1.00	0.98	0.97	0.99	0.99	72	93	2	0	72	95	$p < 2.2e^{-16}$
R63	1.00	0.99	0.99	0.99	0.99	72	94	1	0	72	95	$p < 2.2e^{-16}$

R65	1.00	0.97	0.96	0.98	0.98	72	92	3	0	72	95	$p < 2.2e^{-16}$
R67	1.00	0.97	0.96	0.98	0.98	72	92	3	0	72	95	$p < 2.2e^{-16}$
R69	1.00	0.99	0.99	0.99	0.99	72	94	1	0	72	95	$p < 2.2e^{-16}$
R71	0.99	0.98	0.97	0.98	0.98	71	93	2	1	72	95	$p < 2.2e^{-16}$
R73	1.00	0.99	0.99	0.99	0.99	72	94	1	0	72	95	$p < 2.2e^{-16}$
R75	1.00	0.97	0.96	0.98	0.98	72	92	3	0	72	95	$p < 2.2e^{-16}$
R77	1.00	0.99	0.99	0.99	0.99	72	94	1	0	72	95	$p < 2.2e^{-16}$
R79	1.00	0.99	0.99	0.99	0.99	72	94	1	0	72	95	$p < 2.2e^{-16}$
R81	1.00	0.99	0.99	0.99	0.99	72	94	1	0	72	95	$p < 2.2e^{-16}$
R83	1.00	0.99	0.99	0.99	0.99	72	94	1	0	72	95	$p < 2.2e^{-16}$
R85	1.00	0.97	0.96	0.98	0.98	72	92	3	0	72	95	$p < 2.2e^{-16}$
R87	1.00	0.97	0.96	0.98	0.98	72	92	3	0	72	95	$p < 2.2e^{-16}$
R89	1.00	0.99	0.99	0.99	0.99	72	94	1	0	72	95	$p < 2.2e^{-16}$
R91	1.00	0.97	0.96	0.98	0.98	72	92	3	0	72	95	$p < 2.2e^{-16}$
R93	1.00	0.97	0.96	0.98	0.98	72	92	3	0	72	95	$p < 2.2e^{-16}$
R95	1.00	0.99	0.99	0.99	0.99	72	94	1	0	72	95	$p < 2.2e^{-16}$
R97	1.00	0.99	0.99	0.99	0.99	72	94	1	0	72	95	$p < 2.2e^{-16}$
R99	1.00	0.99	0.99	0.99	0.99	72	94	1	0	72	95	$p < 2.2e^{-16}$
R101	1.00	0.99	0.99	0.99	0.99	72	94	1	0	72	95	$p < 2.2e^{-16}$
R103	1.00	0.99	0.99	0.99	0.99	72	94	1	0	72	95	$p < 2.2e^{-16}$
R105	1.00	0.99	0.99	0.99	0.99	72	94	1	0	72	95	$p < 2.2e^{-16}$
R107	1.00	0.99	0.99	0.99	0.99	72	94	1	0	72	95	$p < 2.2e^{-16}$
R109	1.00	0.99	0.99	0.99	0.99	72	94	1	0	72	95	$p < 2.2e^{-16}$
R111	1.00	0.99	0.99	0.99	0.99	72	94	1	0	72	95	$p < 2.2e^{-16}$
R113	1.00	0.99	0.99	0.99	0.99	72	94	1	0	72	95	$p < 2.2e^{-16}$

R115	1.00	0.99	0.99	0.99	0.99	72	94	1	0	72	95	$p < 2.2e^{-16}$
R117	1.00	0.99	0.99	0.99	0.99	72	94	1	0	72	95	$p < 2.2e^{-16}$
R119	1.00	0.99	0.99	0.99	0.99	72	94	1	0	72	95	$p < 2.2e^{-16}$
R121	1.00	0.99	0.99	0.99	0.99	72	94	1	0	72	95	$p < 2.2e^{-16}$
R123	1.00	0.99	0.99	0.99	0.99	72	94	1	0	72	95	$p < 2.2e^{-16}$
R125	1.00	0.99	0.99	0.99	0.99	72	94	1	0	72	95	$p < 2.2e^{-16}$
R127	1.00	0.96	0.95	0.98	0.97	72	91	4	0	72	95	$p < 2.2e^{-16}$
**Mean**	1.00	0.98	0.97	0.99	0.99							
**Std. Deviation**	0.01	0.01	0.01	0.01	0.01							
